# Computational and experimental analysis of short peptide motifs for enzyme inhibition

**DOI:** 10.1371/journal.pone.0182847

**Published:** 2017-08-15

**Authors:** Jinglin Fu, Luca Larini, Anthony J. Cooper, John W. Whittaker, Azka Ahmed, Junhao Dong, Minyoung Lee, Ting Zhang

**Affiliations:** 1 Department of Chemistry, Rutgers University-Camden, Camden, New Jersey, United States of America; 2 Center for Computational and Integrative Biology, Rutgers University-Camden, Camden, New Jersey, United States of America; 3 Department of Physics, Rutgers University-Camden, Camden, New Jersey, United States of America; nanyang technological university, SINGAPORE

## Abstract

The metabolism of living systems involves many enzymes that play key roles as catalysts and are essential to biological function. Searching ligands with the ability to modulate enzyme activities is central to diagnosis and therapeutics. Peptides represent a promising class of potential enzyme modulators due to the large chemical diversity, and well-established methods for library synthesis. Peptides and their derivatives are found to play critical roles in modulating enzymes and mediating cellular uptakes, which are increasingly valuable in therapeutics. We present a methodology that uses molecular dynamics (MD) and point-variant screening to identify short peptide motifs that are critical for inhibiting β-galactosidase (β-Gal). MD was used to simulate the conformations of peptides and to suggest short motifs that were most populated in simulated conformations. The function of the simulated motifs was further validated by the experimental point-variant screening as critical segments for inhibiting the enzyme. Based on the validated motifs, we eventually identified a 7-mer short peptide for inhibiting an enzyme with low μM IC_50_. The advantage of our methodology is the relatively simplified simulation that is informative enough to identify the critical sequence of a peptide inhibitor, with a precision comparable to truncation and alanine scanning experiments. Our combined experimental and computational approach does not rely on a detailed understanding of mechanistic and structural details. The MD simulation suggests the populated motifs that are consistent with the results of the experimental alanine and truncation scanning. This approach appears to be applicable to both natural and artificial peptides. With more discovered short motifs in the future, they could be exploited for modulating biocatalysis, and developing new medicine.

## Introduction

Cellular functions highly rely on enzymes to make molecules and derive energy that are vital to metabolism and reproduction of living systems.[1, 2] Regulation of enzyme activity is central to therapeutics and drug discovery.[3, 4] High-throughput screening or selection of a vast molecule library is widely used to identify ligands that are able to bind to proteins and modulate their functions, including in vitro mRNA display,[5] phage display,[6] bead-based library screening,[7] protein directed evolution,[8] aptamer selection[9] and fragment-based design of small molecules.[10] These approaches generally require either multiple selection cycles over large chemical libraries (10^6^ or more) or the structural information of proteins, which extend the completion time and increase the cost. Recent developments of microarray technology have allowed the screening of small molecules, peptides, proteins and nucleic acids for identifying ligands that can bind to a protein target. [11–13] Peptides represent a promising class of potential enzyme modulators[14] due to the large chemical diversity[15], and well-established methods for library synthesis.[16] Peptides and their derivatives are found to play critical roles in modulating enzymes and mediating cellular uptakes, which are increasingly valuable in therapeutics.[17] In this work, we presented a methodology that combined the molecular dynamic (MD) simulations and point-variant screening to identify short peptide motifs for inhibiting enzymes.

## Results

**Fig 1:**
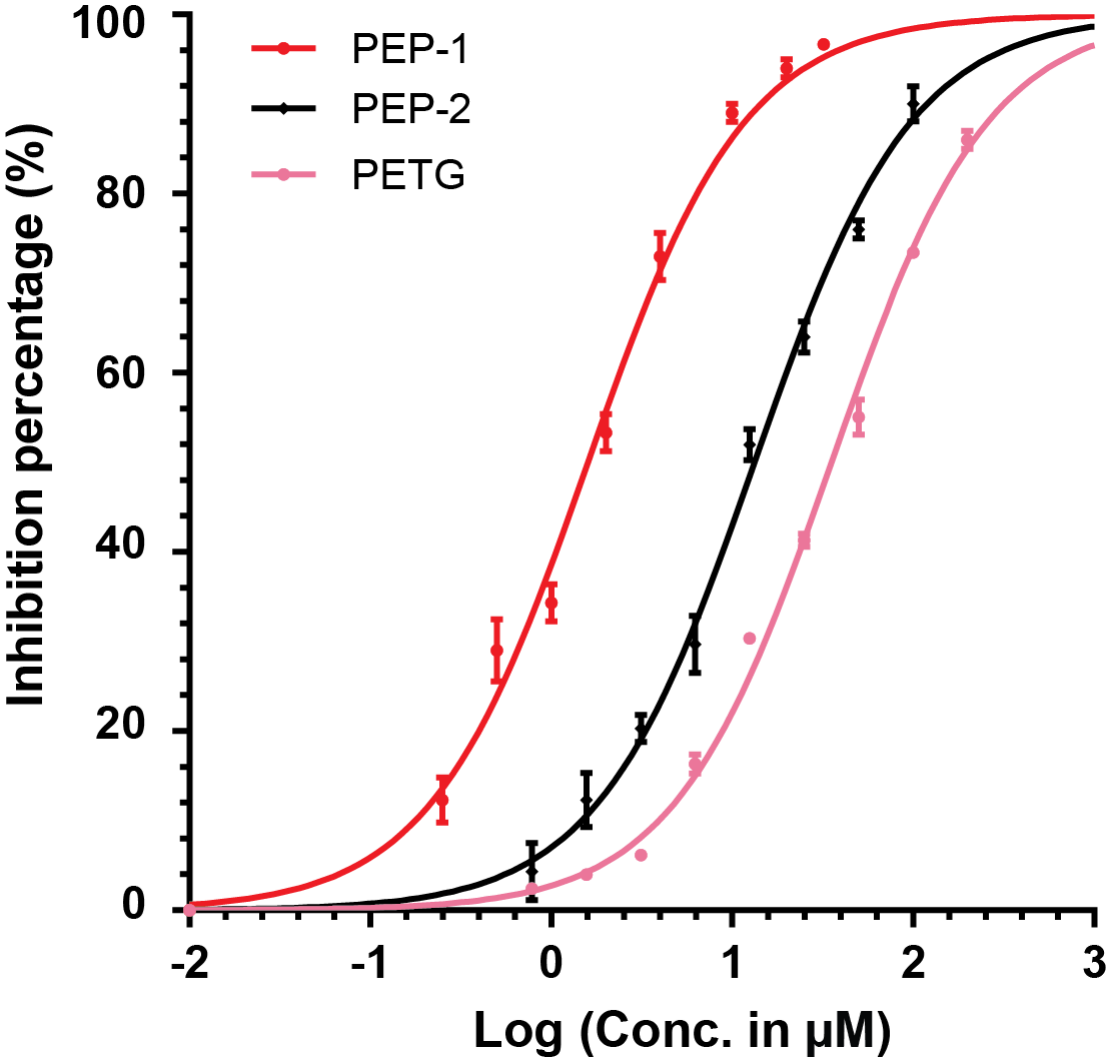
Selected lead peptides of PEP-1 and PEP-2 for inhibiting β-Gal. The IC_50_ of peptide inhibition is measured at a substrate concentration of 100 μM RBG (resorufin β-D-galactopyranoside) and β-Gal concentration of 150 μg/L, 25°C. PETG is a known competitive inhibitor of β-Gal. All tests included three replicates. Error bar: range of data.

The functional motifs were predicted based on the simulated conformations of lead peptides. Two 20-mer lead peptides (PEP-1: RVFKRYKRWLHVSRYYFGSC; PEP-2: PASMFSYFKKQGYYYKLGSC) were previously selected from a microarray of 10,000 peptides for inhibiting β-Gal with IC_50_ values ~ 1.6 μM and 13 μM (Fig 1A).[13] Both of the two peptides were stronger inhibitors than phenylethyl β-D-thiogalactoside (PETG, IC_50_ ~ 35 μM), a known competitive inhibitor of β-Gal.[18]

**Fig 2:**
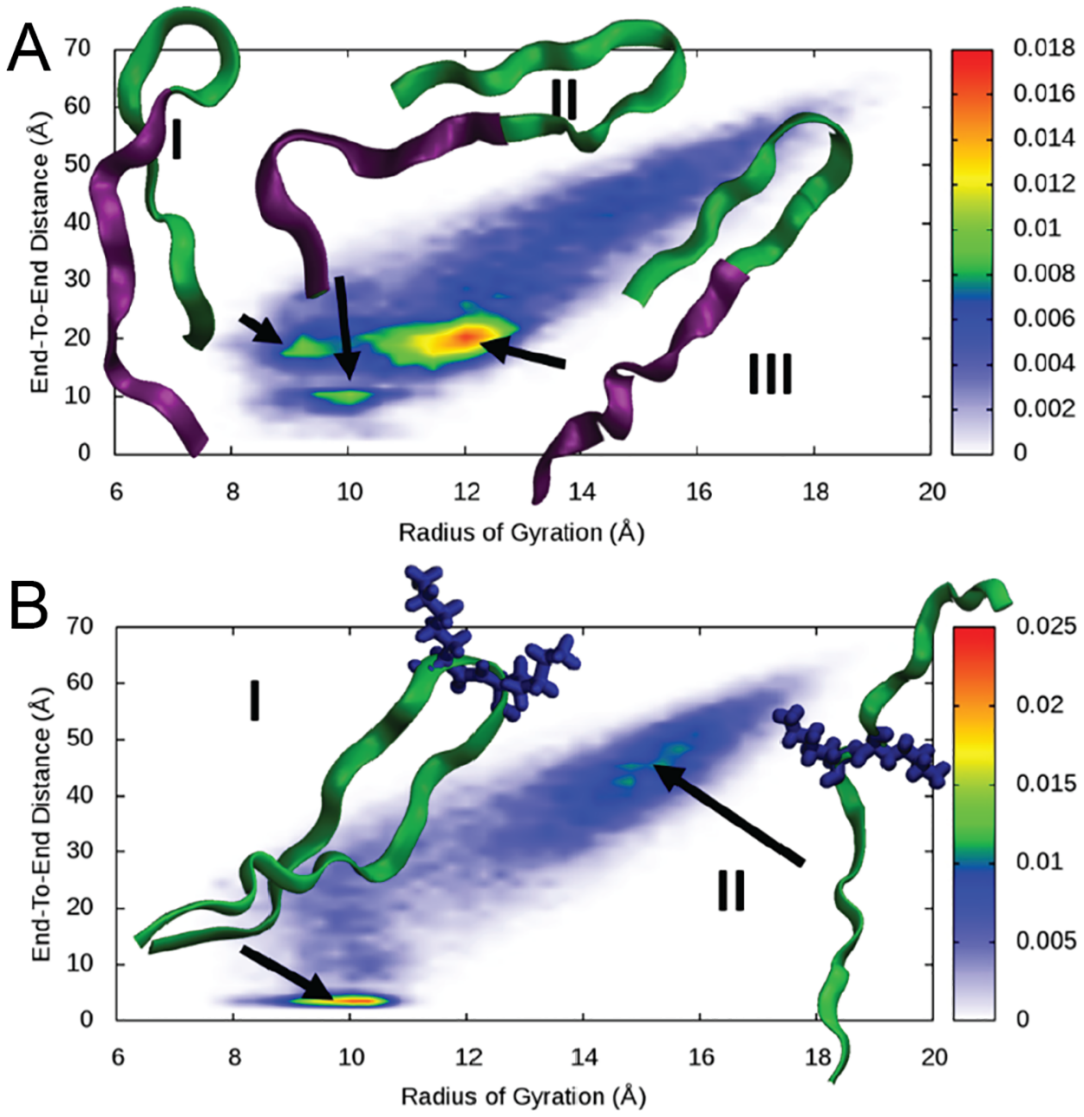
Normalized distribution of the end-to-end and radius of gyration plot for PEP-1 (A) and PEP-2(B). PEP-1 populates three major clusters characterized by β-hairpin conformations. The N-terminus (RVFKRYKRW, labelled in purple) is usually fully exposed to the solvent. PEP-2 conformations are dominated by the repulsion of the two central lysines. The most stable conformations are characterized by the two lysines pointing in opposite direction (side chains reported in blue) in order to minimize their repulsion.

To analyse the critical motifs for inhibiting enzyme, MD was used to simulate the conformations of two selected peptides in aqueous solution. As shown in Fig 2A, PEP-1 showed three most populated conformations, all with highly positively charged N-terminus fragments (labelled with purple) that were fully exposed to the solvent. The C-terminus of PEP-1 (labelled with green) adopted a β-hairpin conformation that was rich in hydrophobic moieties, which induced its compact structure. In contrast, the abundance of positively charged residues in N-terminus fragments created a repulsive force that extended the linear structure. In these populated conformations, an interesting exposable N-terminal motif (RVFKRYKRW) was observed, which were suggested to be responsible for inhibiting β-Gal. As shown in Fig 2B, PEP-2 was mainly populated by two conformations: conformation I tended to adopt a β-hairpin while conformation II tended to adopt an extended structure. The transition between two conformations happened via a two-lysine portion (KK) in the middle of the sequence. The two lysine residues pointed to the opposite direction that constrained the adopted conformations of PEP-2. It was expected that the removal of these two lysines would affect the adopted conformations of PEP-2 for inhibiting β-Gal. For both peptides, C-terminal “GSC” link was used to couple peptides onto microarray, and it was assumed not to affect the function of peptides.[13, 19]

Since β-Gal was a negatively charged enzyme (pI ~ 4.7) at neutral pH condition, we suggested that positively charged residues of peptides (e.g. lysine, K; or arginine, R) would be more critical for interacting with the enzyme via charge interaction. Based on the sequences of two peptides, N-terminal motif of PEP-1 (RVFKRYKRW) contained most of the positively charged residues. Thus, it was predicted to be critical for inhibiting β-Gal. Further MD modelling of PEP-1 truncation sequences suggested that the activity of the peptides were correlated with the population of the linearly structured N-terminal motif in the simulated conformations (S1 Fig). PEP-2 was divided into two motifs by KK residues: N-terminal PASMFSYF and C-terminal KKQGYYYKL. C-terminal motif of PEP-2 was suggested to be more critical for inhibiting enzyme due to the rich K residues.

**Fig 3:**
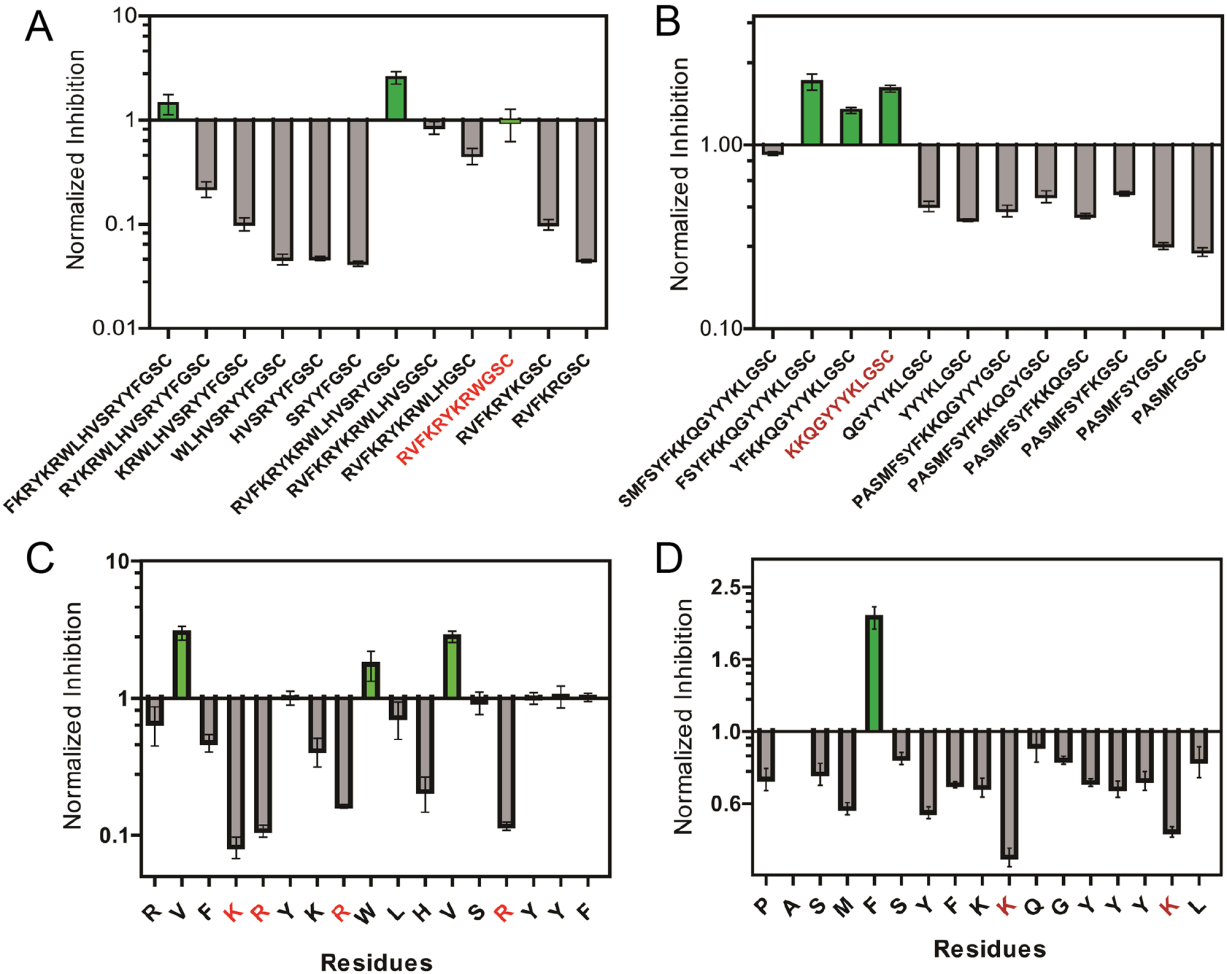
Sequential truncation and alanine scan of PEP-1 and PEP-2 for inhibiting β-Gal activity. (A) Truncation scans of PEP-1 and (B) PEP-2 for inhibiting β-Gal. (C) Alanine scans of PEP-1 and (D) PEP-2 for inhibiting β-Gal. All inhibitions of alanine-substituted peptides and truncated peptides were normalized to the inhibition percentage values of PEP-1 at 10 μM and PEP-2 at 50 μM, respectively. All tests included three replicates. Error bar: range of data.

To validate the above modelling results, we performed a set of truncation and alanine-scan analysis. The truncation analysis was performed by sequentially removing two residues each time from either N-terminus or C-terminus. The C-terminus GSC linker was used to anchor the peptide on the microarray surface, and thus it was kept the same for all truncation sequences. As shown in Fig 3A, the truncation analysis of PEP-1 revealed a 12-mer “RVFKRYKRWGSC” (“GSC” is a linker) with similar normalized inhibition as the 20-mer PEP-1. This short truncation sequence was exactly the same N-terminus motif predicted by the MD simulation. As shown in Fig 3B, the truncation analysis of “PEP-2” revealed a shorter C-terminus motif of “KKQGYYYKLGSC” that inhibited β-Gal. Further removal of “KK” resulted in a significant decrease of the normalized inhibition. This result was consistent with our modelled “KK” pair for inhibiting β-Gal. Next, alanine scanning was used to examine the dependence of the inhibition on the specific amino acid residues for PEP-1 and PEP-2. As shown in Fig 3C, an alanine scan of PEP-1 revealed that positively-charged residues at positions 4 (K), 5 (R), 8 (R) and 14 (R) played critical roles for inhibiting β-Gal activity. Substitutions of these residues with an alanine significantly decreased the ability of the peptide to inhibit the enzyme by 5–10 fold. As shown in Fig 3D, the similar effect was also observed for PEP-2 that positively-charged lysine residues at positions 10 (K) and 16 (K) were most important for inhibiting β-Gal activity. Since β-Gal is a low pI (~ 4.7) protein with a negatively charged surface at neutral pH, this enzyme could bind strongly to a variety of positively charged surfaces and peptides.[20, 21] The alanine scan results were consistent with these reports. Detailed inhibition kinetics for truncation and alanine analysis are shown in S2–S5 Figs and S1–S4 Tables.

**Fig 4:**
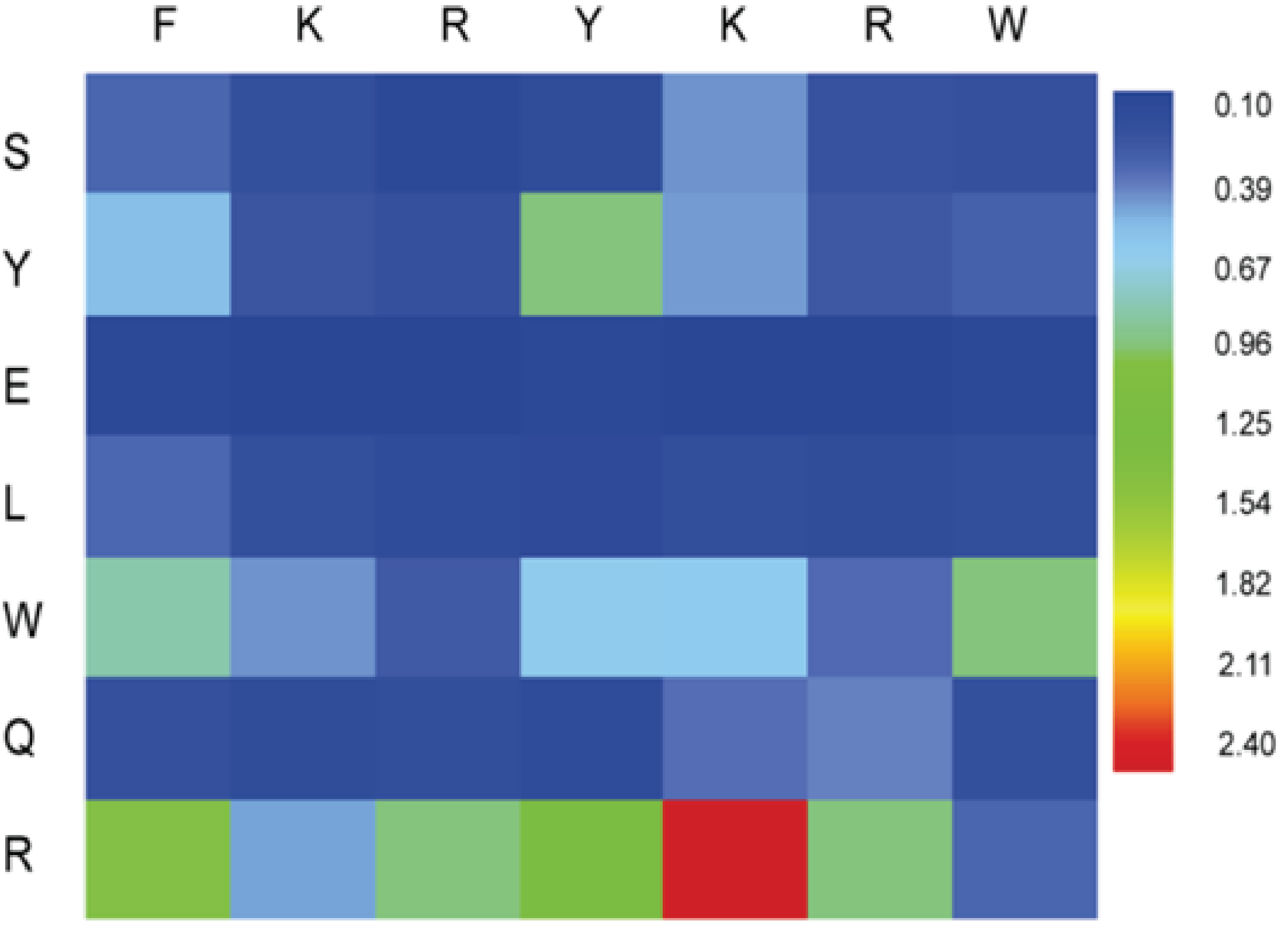
Point-variant screening of nPEP-1 “FKRYKRWGSC”. The point-variant screening is performed at each of the 7 N-terminal positions with a substitution set of S, Y, E, L, W, Q, and R. The inhibition of β-Gal by variants was normalized to the inhibition percentage value of the nPEP-1 at 20 μM.

In the truncation analysis of PEP-1 (Fig 3A), the removal of N-terminus “RV” residues did not significantly affect the inhibition of β-Gal. Based on this, the 12-mer motif of “RVFKRYKRWGSC” was further minimized to a 10-mer motif of “FKRYKRWGSC” (nPEP-1) that was still able to inhibit β-Gal with a IC_50_ ~ 4 μM (S6 Fig). As shown in Fig 4, the point-variant screening[22, 23] was applied to this new peptide where a library of 49 single-point variants was synthesized that contained all substitutions on each of the seven positions (FKRYKRW) with the amino acid set {S, Y, E, L, W, Q and R}. The small amino acid set represented different properties of amino acids: serine (S) and glutamine (Q) were selected for their polar uncharged side chain; tyrosine (Y) and tryptophan (W) were selected for their aromatic side chain; glutamate (E) was selected for its negative charge; arginine (R) was selected for its positive charge; leucine (L), tryptophan (W) and glutamine (Q) could also span the hydropath range.[22, 24] The small set reduced the cost of the substitutions with all 20 natural standard amino acids. Comparing these single-point variants with nPEP-1 for inhibiting β-Gal, most of the mutations did not enhance the enzyme inhibition, except for the replacement of 5K with an arginine (R). This variant showed a stronger inhibition of β-Gal than nPEP-1, with a IC_50_ ~ 2.5 μM (S6 Fig). For such a short peptide of 7-mer variable region, it is difficult to improve the peptide activity with further sequence optimization. Thus, we ended the optimization of short peptides with the 5K(R) substitution. Detailed inhibition kinetics for point variants can be found in S7 Fig and S5 Table.

## Discussion

In summary, we described a method that combined MD simulations and experimental optimization to rapidly identify short peptide motifs for inhibiting enzymes. For proof of concept, we have focused on the peptides for inhibiting β-gal. Lead peptides can be rapidly selected from a microarray of a relatively small library of peptides. MD simulation of the lead peptide suggested that a 7-mer linear short motif was critical for peptide activity. This motif was presented in the most populated conformations. Consistent with this modelling result, the same motif was also identified by the alanine and truncation scanning of the lead peptide, and was validated by inhibiting enzyme at low μM IC_50_ value. The point-variant screening showed a 5K(R) substitution further increased the inhibition ability of the short motif. Comparing with other methods of ligand-protein interactions, our presented approach does not require the detailed structural information of the enzyme target and the ligand-protein complexes, thereby reducing the selection complication, cost and time. The identified short peptide motifs can be easily synthesized in large scale with high purity. Although the presented approach appears to be applicable to both natural and artificial peptides, further studies are still required to validate this methodology with other peptides and enzymes. With more discovered short motifs, they could be exploited for modulating biocatalysis, and developing new medicine.

## Materials and methods

### Enzyme inhibition assays

Solution-based enzyme assays were performed on SpectraMax M5 96 well plate readers (Molecular Device, Sunnyvale, CA) as described previously.[13] Briefly, peptides were first incubated with enzyme for 20 minutes, then the substrate solution was added into the wells to measure the enzyme activity, including at least three replicates per peptide. The β-gal catalyzed hydrolysis of RBG was fluorescently monitored at 590 nm (resorufin) with the excitation at 540 nm. The velocity of the reaction was determined by the initial velocity of the linear reaction. The IC_50_ of each inhibitor was determined by fitting the concentration vs. inhibition curve to the function ‘Fit LogIC50’ as defined in the program GraphPad Prism 7 using the fitting equation “Y = Bottom+(Top-Bottom)/(1+10^(X-LogIC50))”. The “Bottom” term was constrained to 1, which represents the maximal inhibition of 100%. The “Top” term was constrained to 0, which represents the minimal inhibition of 0%.

The enzyme inhibition was also quantitatively characterized using the inhibition percentage that was calculated using the following equation:

$$\text{Inhibition Percentage }=\;\left({\text{Activity}}_{\text{uninhibited}}-{\text{ Activity}}_{\text{inhibited}}\right)/{\text{ Activity}}_{\text{uninhibited}}\times \;100\%$$

### Molecular dynamics

The simulations were performed using the AMBER 14 package[18] with the AMBER96 force field[19] in an implicit solvent model. As an implicit solvent model we chose the IGB = 5 set of parameters associated with gbsa = 1.[20] Simulations were performed in pure water. A cutoff of 20Å was used for the implicit solvent model, the dispersion forces and the electrostatics. The temperature was kept constant using a Langevin bath with a characteristic time of 1 ps^-1^. In order to increase the sampling of the conformations we have applied Replica Exchange Molecular Dynamics (REMD).[21] Exchanges are attempted every 3 ps and we use 8 temperatures (282, 300, 325, 353, 383, 415, 448, 484 K). The equations of motion were integrated using a time step of 1 fs. The bonds between hydrogen atoms and heavy atoms were kept constant using the SHAKE algorithm.[22] Simulations were 600 ns long and we analyzed only the final 300 ns.

## Supporting information

S1 File A detailed description of the methodology used. (PDF)

S1 Fig MD simulation of the PEP-1 truncated sequences. (PDF)

S2 Fig Raw data of truncation analysis for PEP-1. (PDF)

S3 Fig Raw data of truncation analysis for PEP-2. (PDF)

S4 Fig Raw data pf alanine scan for PEP-1. (PDF)

S5 Fig Raw data pf alanine scan for PEP-2. (PDF)

S6 Fig Determination of IC_50_. (PDF)

S7 Fig Point variant screening. (PDF)

S1 Table Truncation library for PEP-1. (PDF)

S2 Table Truncation library for PEP-2. (PDF)

S3 Table Alanine scan library for PEP-1. (PDF)

S4 Table Alanine scan library for PEP-2. (PDF)

S5 Table Point variant library. (PDF)
