# Supplementary material for: Computational and experimental analysis of short peptide motifs for enzyme inhibition
Source: PLoS One. 2017 Aug 15;12(8):e0182847. doi: 10.1371/journal.pone.0182847 (PMC5557489; doi:10.1371/journal.pone.0182847)
Supplement: S1 File — (PDF) [file pone.0182847.s001.pdf]

Materials

Fluorescein di-β-D-galactopyranoside (FDG), resorufin β-D-galactopyranoside (RBG) and Alexa Fluor 647 (Alexa 647) were purchased from Invitrogen (Eugene, OR). Phenylethyl β-D-thiogalactoside (PETG), β-galactosidase (β-Gal), phosphate buffered saline (PBS) and tris buffered saline (TBS) were obtained from Sigma (St. Louis, MO). A 4 mg/mL stock solution of β-Gal was prepared in 10 mM potassium phosphate buffer with 0.1 mM $MgCl_2$ at pH 7.4. A 2 mg/mL stock solution of peptide was first prepared in pure water, and then diluted in phosphate buffer to the desired concentration. Custom peptides were purchased from Sigma.
