# Supplementary material for: Computational and experimental analysis of short peptide motifs for enzyme inhibition
Source: PLoS One. 2017 Aug 15;12(8):e0182847. doi: 10.1371/journal.pone.0182847 (PMC5557489; doi:10.1371/journal.pone.0182847)
Supplement: S1 Fig — (PDF) [file pone.0182847.s002.pdf]

S1 Fig. MD simulation of the PEP-1 truncated sequences.

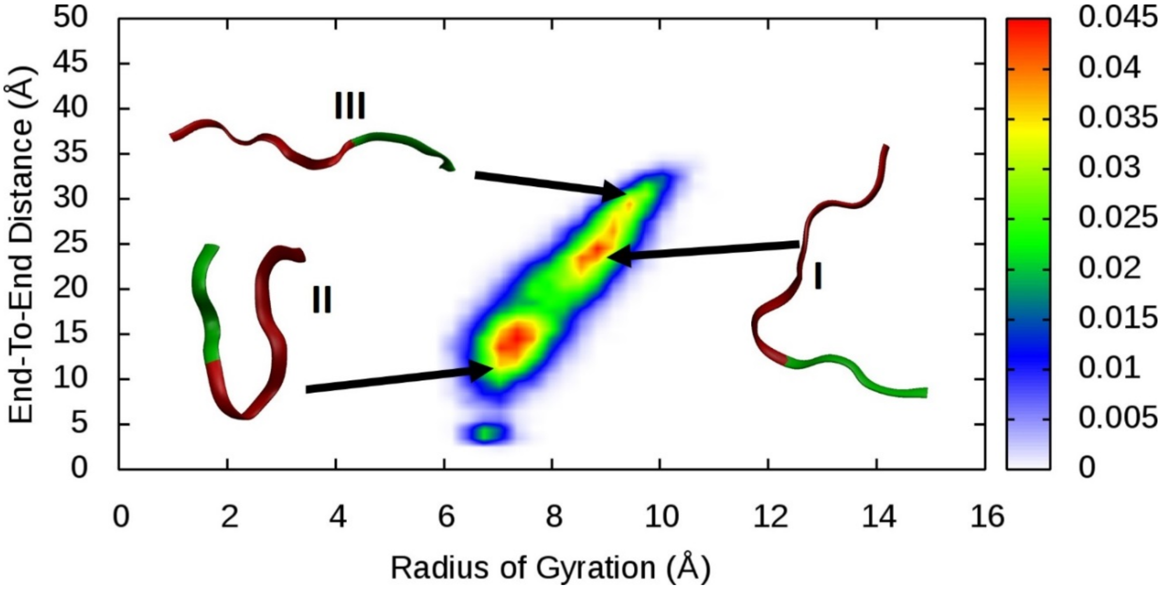

Figure S1-A. MD simulation of the truncated PEP-1: FKRYKRWGSC (10-mer). The section highlighted in red color corresponds to the FKRYKRW motif. The section highlighted in green color corresponds to "GSC". The three most abundant clusters have been shown as well (I: 51.6%, II: 22.9%, III: 11.8%)

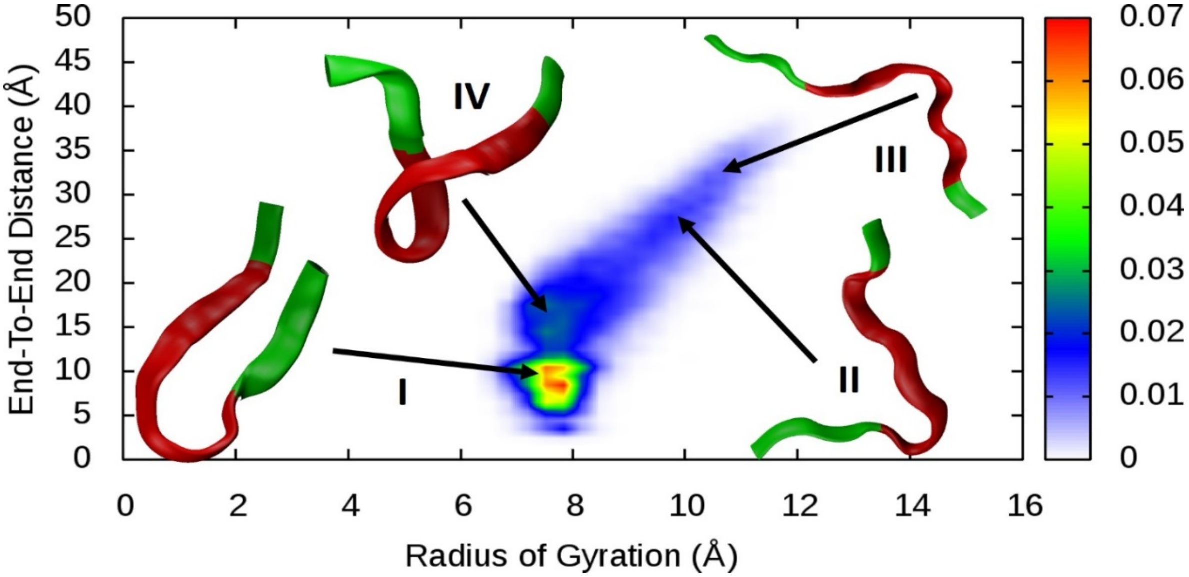

Figure S1-B. MD simulation of the truncated PEP-1: RVFKRYKRWGSC (12-mer). The section highlighted in red color corresponds to the FKRYKRW motif. The most abundant clusters have been shown as well (I: 30.5%, II: 13.1%, III: 8.8%, IV: 8.5%)

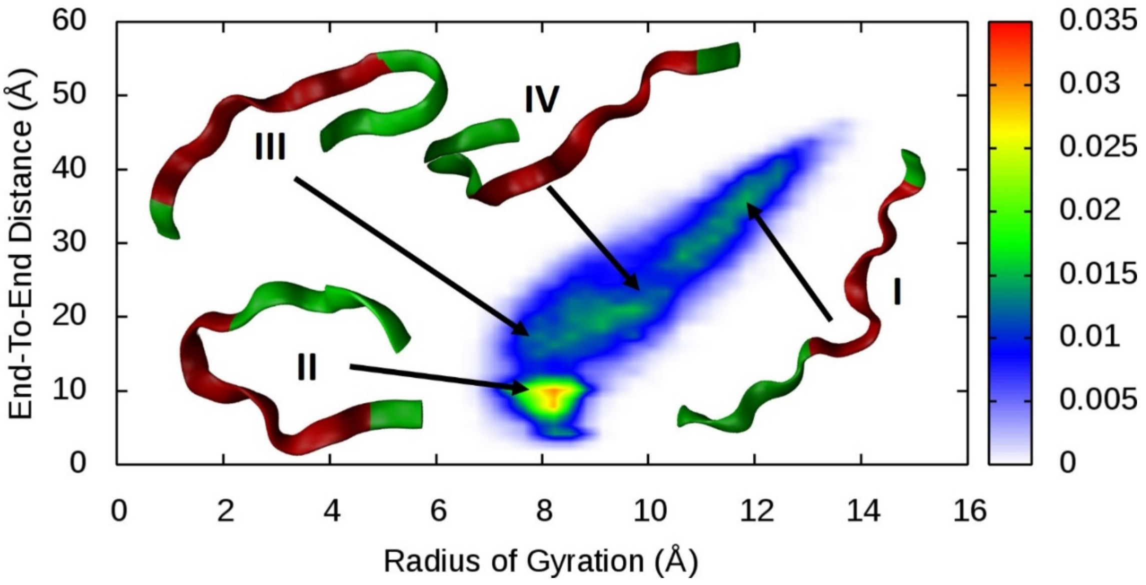

Figure S1-C. MD simulation of the truncated PEP-1: RVFKRYKRWLHGSC (14-mer). The section highlighted in red color corresponds to the FKRYKRW motif. The most abundant clusters have been shown as well (I: 30.9%, II: 21.5%, III: 11.1%, IV: 6%)

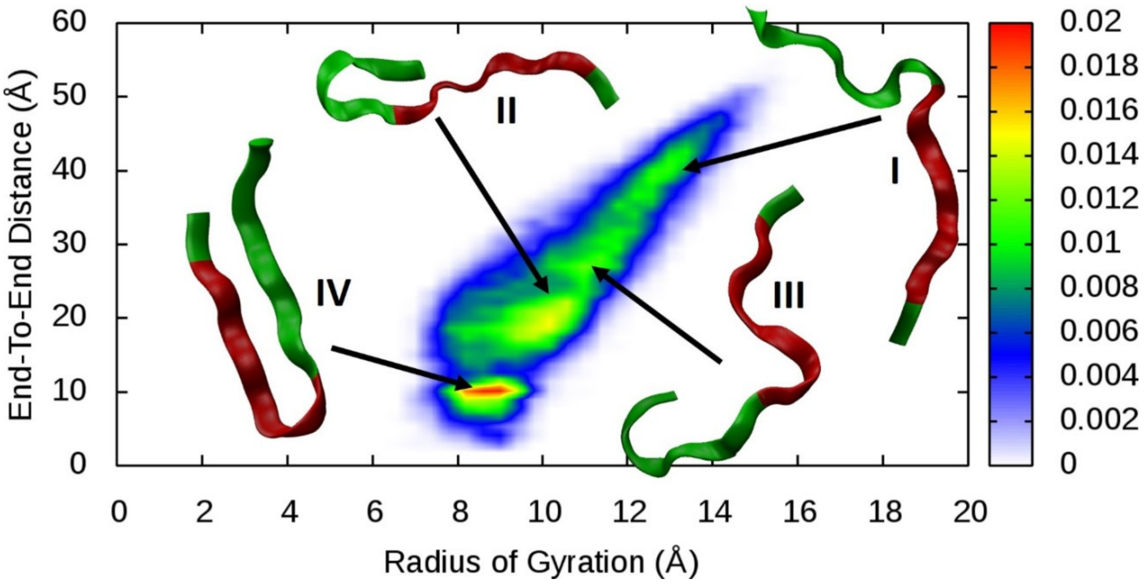

Figure S1-D. MD simulation of the truncated PEP-1: RVFKRYKRWLHVSGSC (16-mer). The section highlighted in red color corresponds to the FKRYKRW motif. The most abundant clusters have been shown as well (I: 6.3%, II: 10.0%, III: 6.4%, IV: 16.2%)

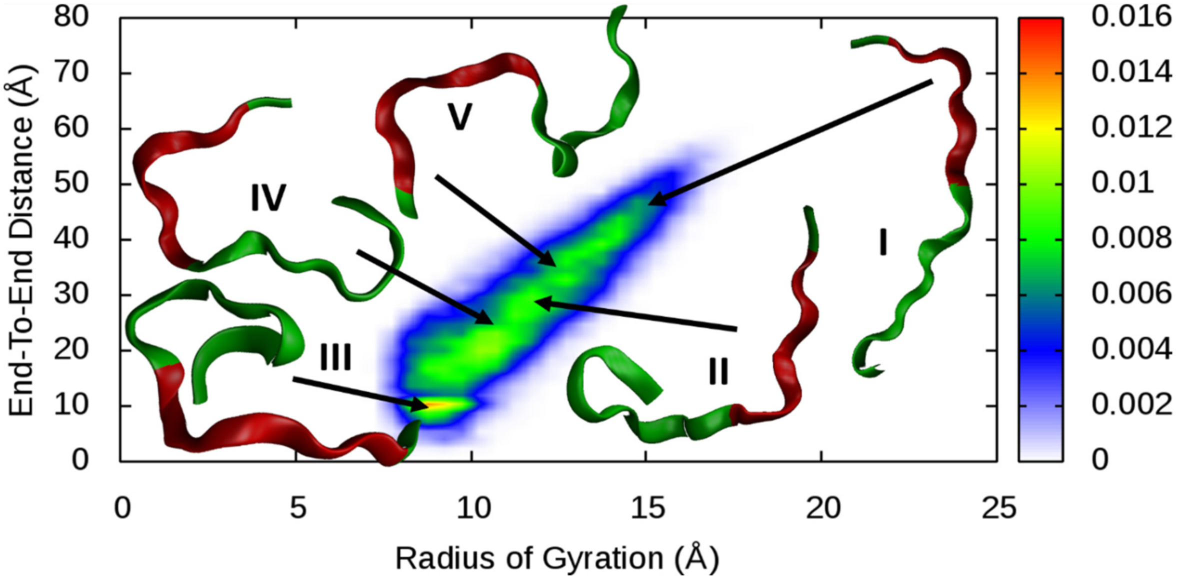

Figure S1-E. MD simulation of the truncated PEP-1: RVFKRYKRWLHVSRYGSC (18-mer). The section highlighted in red color corresponds to the FKRYKRW motif. The five most abundant clusters have been shown as well (I: 27.5%, II: 15.6%, III: 10.2%, IV:8.4%, V:6.9%)

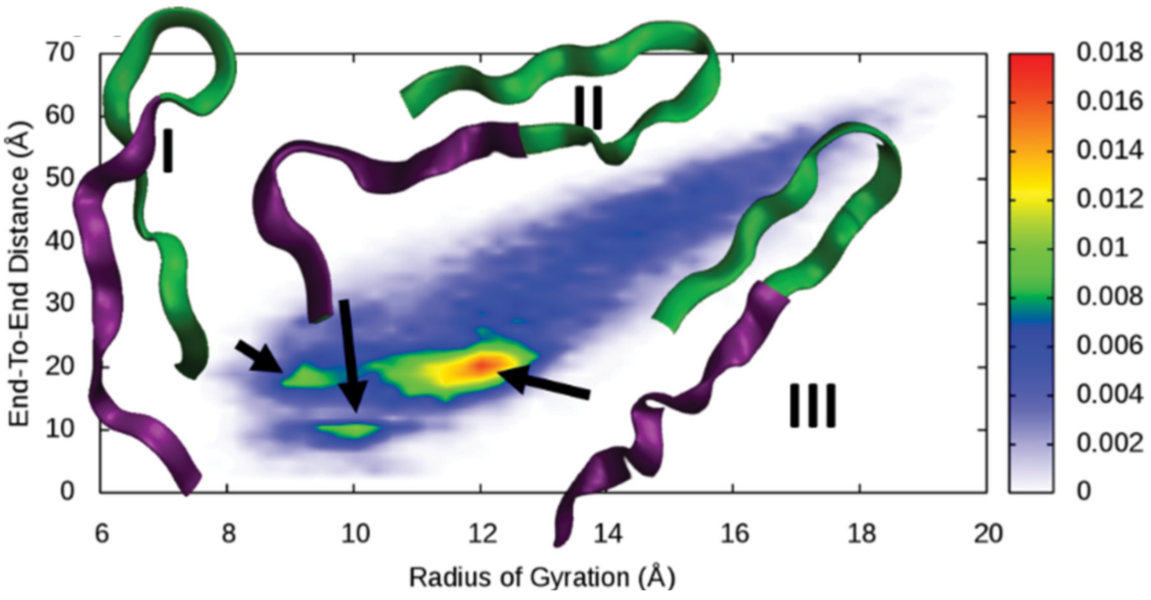

Figure S1-F. MD simulation of PEP-1: RVFKRYKRWLHVSRYGSC (20-mer). The section highlighted in purple color corresponds to the FKRYKRW motif. The three most abundant clusters have been shown as well (I: 7.5%, II: 5.6%, III: 26.9%).
