# Supplementary material for: Computational and experimental analysis of short peptide motifs for enzyme inhibition
Source: PLoS One. 2017 Aug 15;12(8):e0182847. doi: 10.1371/journal.pone.0182847 (PMC5557489; doi:10.1371/journal.pone.0182847)
Supplement: S2 Fig — (PDF) [file pone.0182847.s003.pdf]

S2 Fig. Truncation analysis of PEP-1. 10 µM peptide was incubated with 300 µg/L β-Gal and 100 µM RBG in 10 mM potassium phosphate buffer with 0.1 mM $MgCl_2$ (pH 7.4), 25 °C.

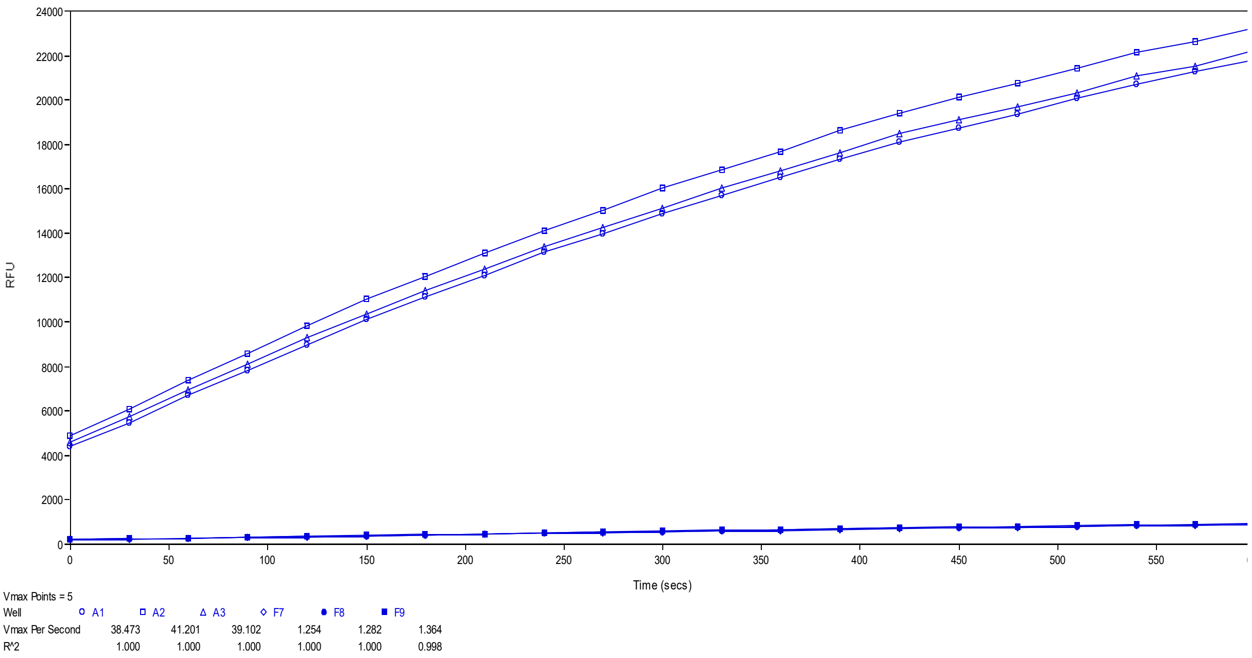

Uninhibited β-Gal activity (top curves) and inhibited β-Gal with 10 µM PEP-1 (bottom curves).

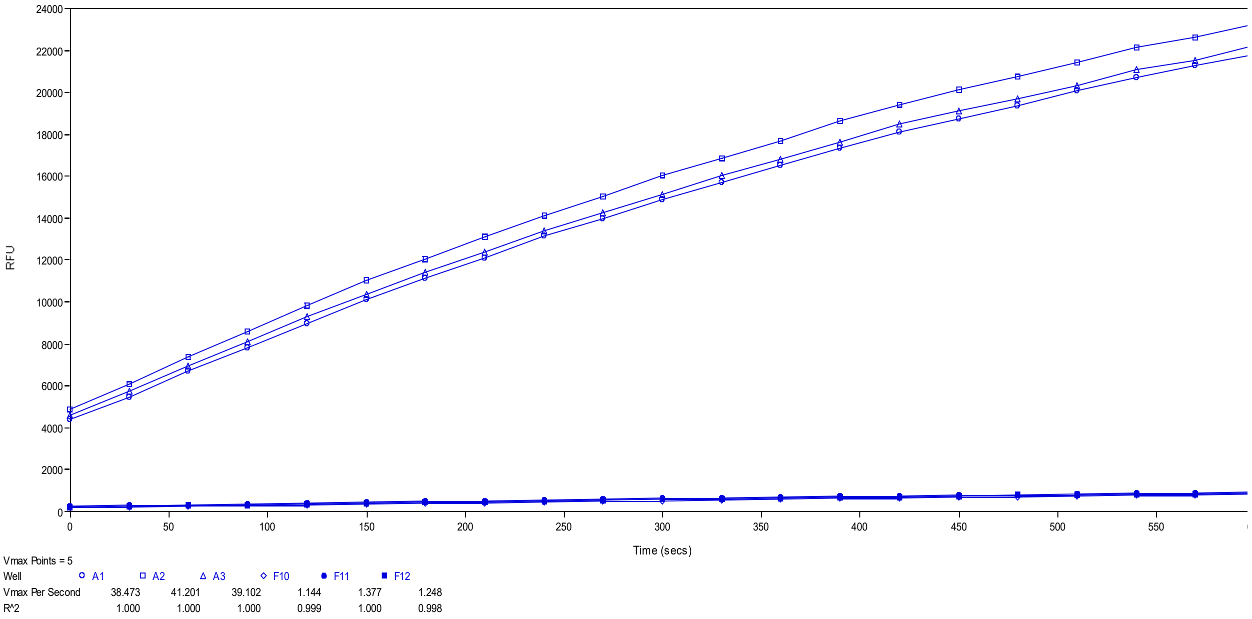

Uninhibited β-Gal activity (top curves) and inhibited β-Gal with 10 µM tPEP-1-1(bottom curves).

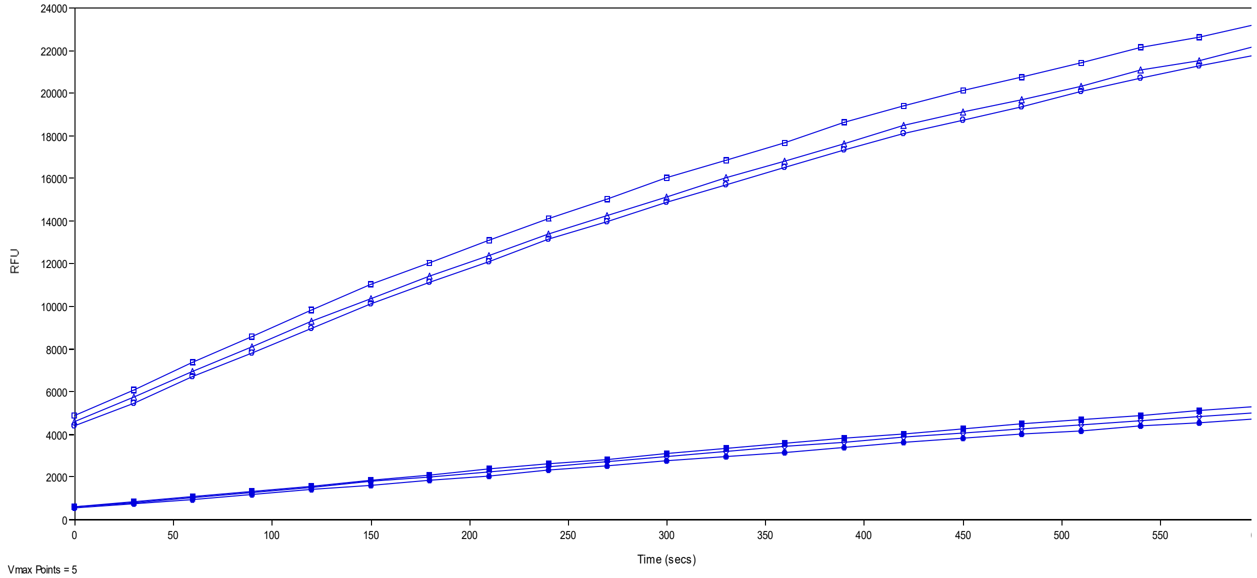

Vmax Points = 5

Well	A1	A2	A3	G4	G5	G6
Vmax Per Second	38.473	41.201	39.102	8.301	7.561	8.722
R^2	1.000	1.000	1.000	0.999	1.000	0.999

Uninhibited β-Gal activity (top curves) and inhibited β-Gal with 10 μM tPEP-1-2 (bottom curves).

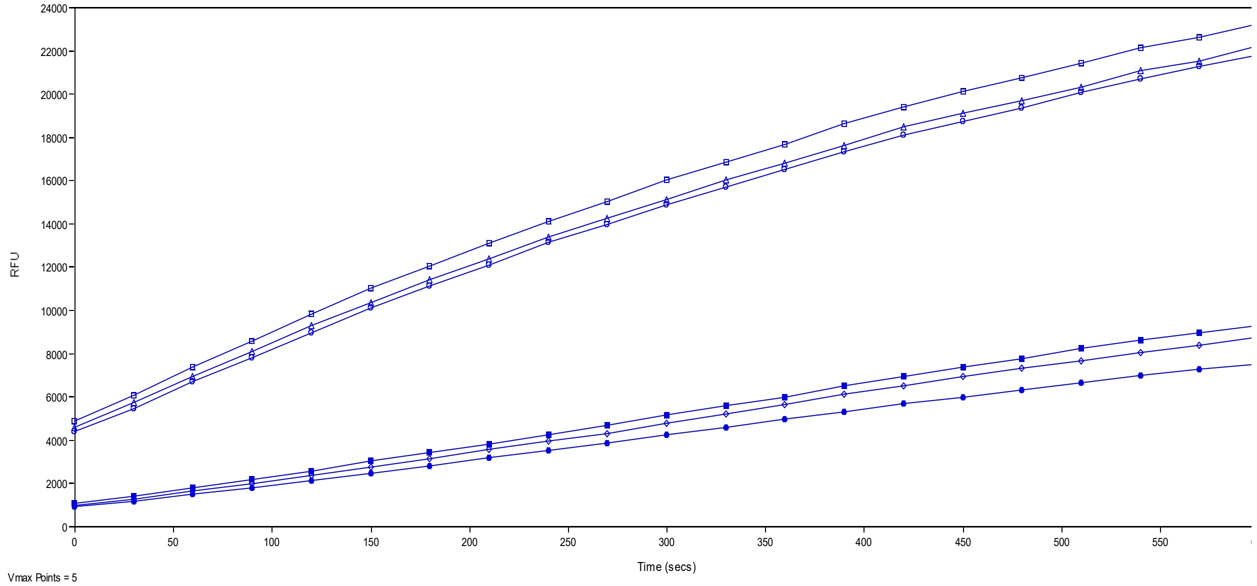

Vmax Points = 5

Well	A1	A2	A3	G7	G8	G9
Vmax Per Second	38.473	41.201	39.102	14.731	12.042	15.131
R^2	1.000	1.000	1.000	1.000	1.000	0.999

Uninhibited β-Gal activity (top curves) and inhibited β-Gal with 10 μM tPEP-1-3 (bottom curves).

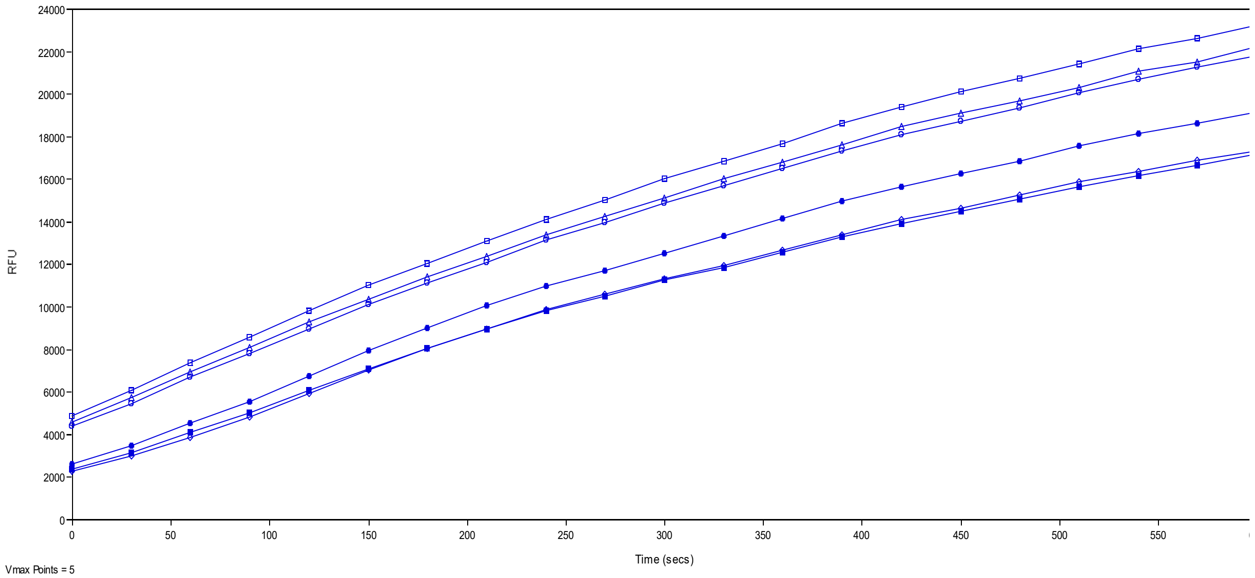

Vmax Points = 5

Well	A1	A2	A3	G10	G11	G12
Vmax Per Second	38.473	41.201	39.102	35.300	37.784	33.436
R^2	1.000	1.000	1.000	0.999	0.999	1.000

Uninhibited β-Gal activity (top curves) and inhibited β-Gal with 10 μM tPEP-1-4 (bottom curves).

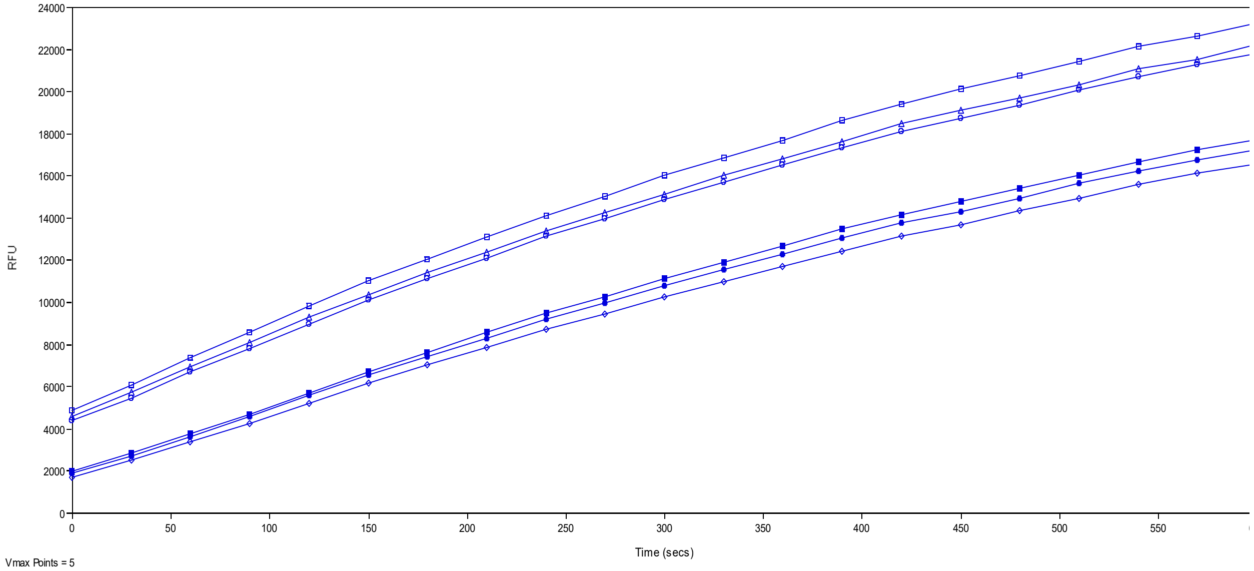

Vmax Points = 5

Well	A1	A2	A3	H4	H5	H6
Vmax Per Second	38.473	41.201	39.102	30.758	32.028	32.469
R^2	1.000	1.000	1.000	0.999	1.000	1.000

Uninhibited β-Gal activity (top curves) and inhibited β-Gal with 10 μM tPEP-1-5 (bottom curves).

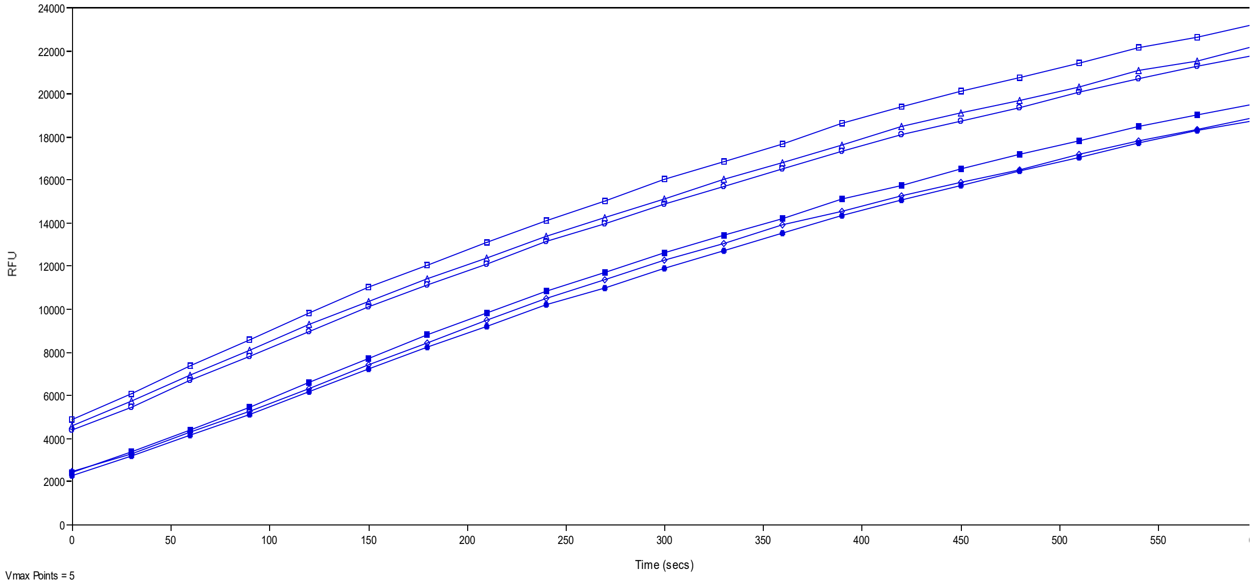

Vmax Points = 5

Well	A1	A2	A3	H7	H8	H9
Vmax Per Second	38.473	41.201	39.102	35.266	34.188	37.024
R^2	1.000	1.000	1.000	1.000	1.000	1.000

Uninhibited β-Gal activity (top curves) and inhibited β-Gal with 10 μM tPEP-1-6(bottom curves).

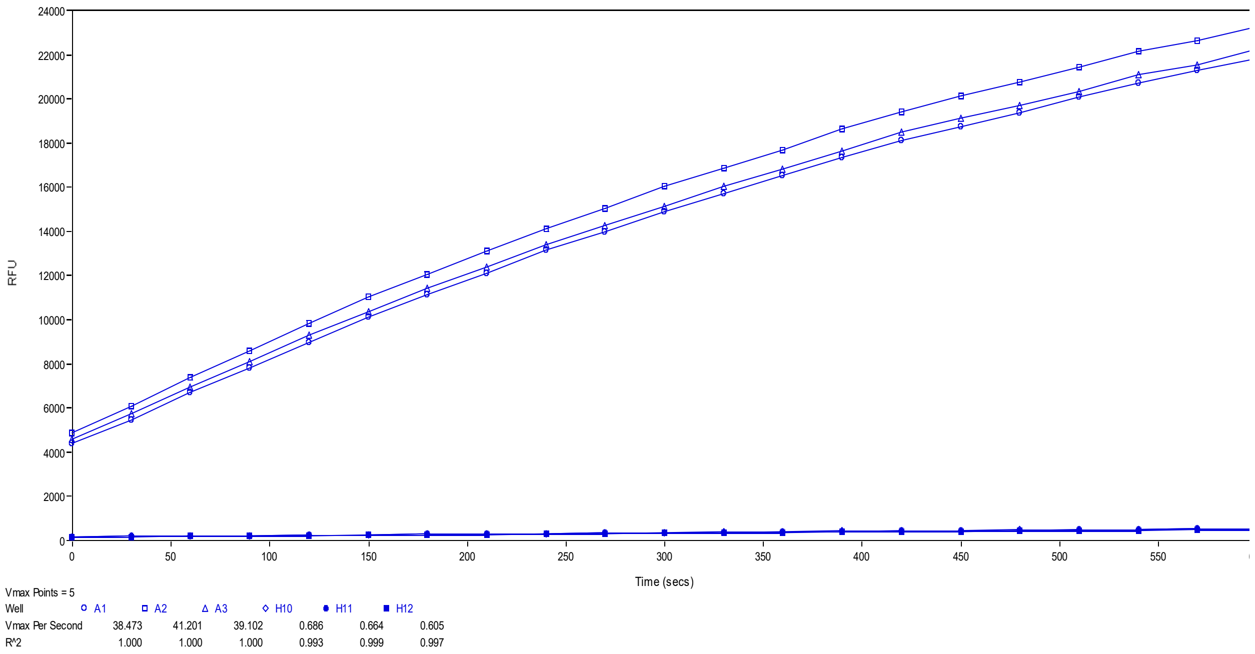

Uninhibited β-Gal activity (top curves) and inhibited β-Gal with 10 μM tPEP-1-7(bottom curves).

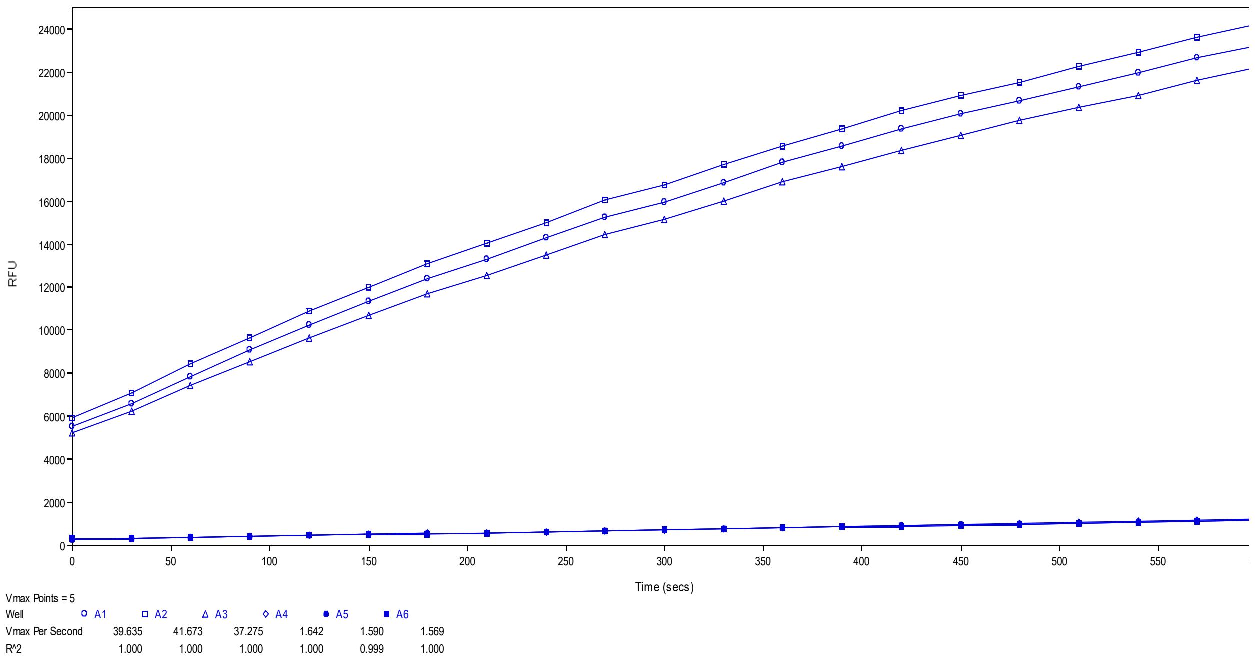

Uninhibited β-Gal activity (top curves) and inhibited β-Gal with 10 μM tPEP-1-8(bottom curves).

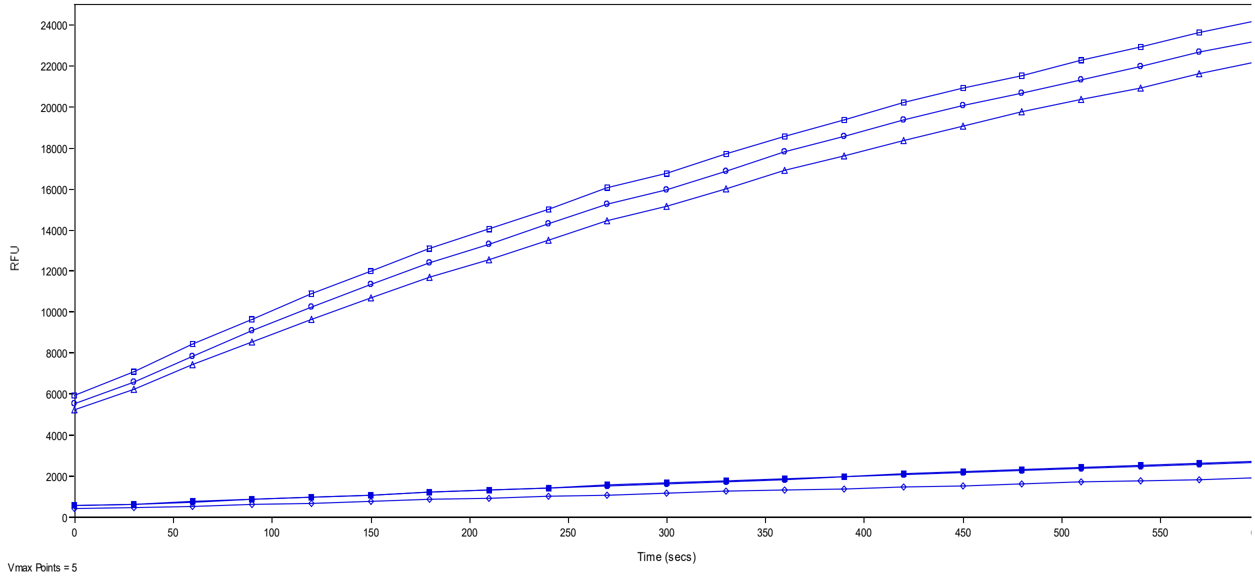

Vmax Points = 5

Well	A1	A2	A3	A7	A8	A9
Vmax Per Second	39.635	41.673	37.275	2.672	3.857	3.951
R^2	1.000	1.000	1.000	0.999	1.000	1.000

Uninhibited β-Gal activity (top curves) and inhibited β-Gal with 10 μM tPEP-1-9 (bottom curves).

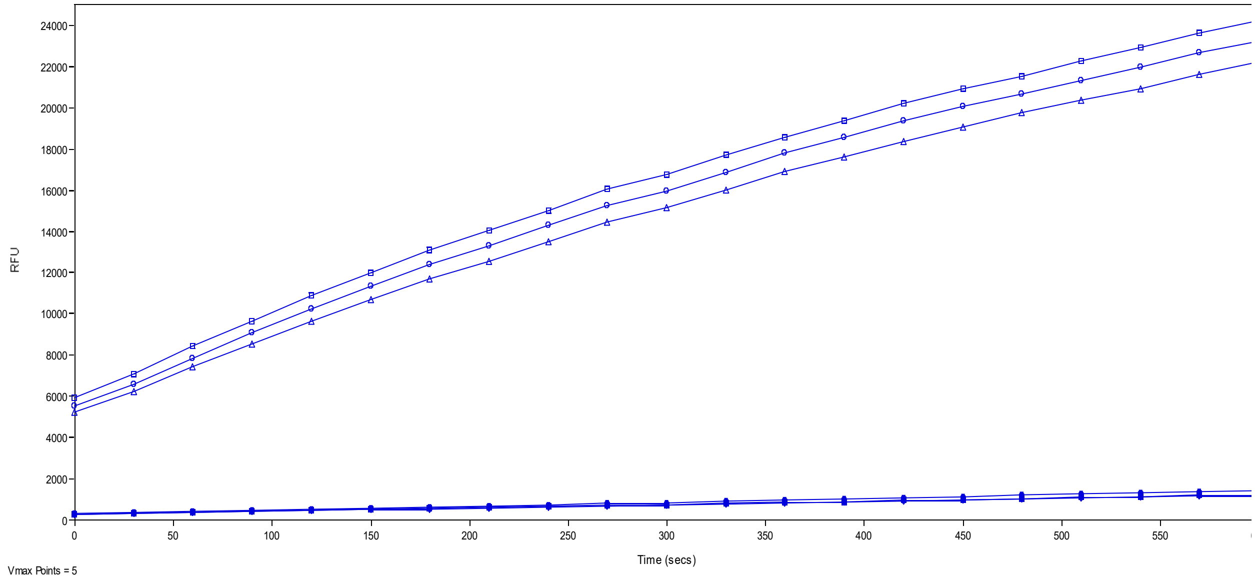

Vmax Points = 5

Well	A1	A2	A3	B4	B5	B6
Vmax Per Second	39.635	41.673	37.275	1.709	2.030	1.756
R^2	1.000	1.000	1.000	1.000	0.998	0.998

Uninhibited β-Gal activity (top curves) and inhibited β-Gal with 10 μM tPEP-1-10 (bottom curves).

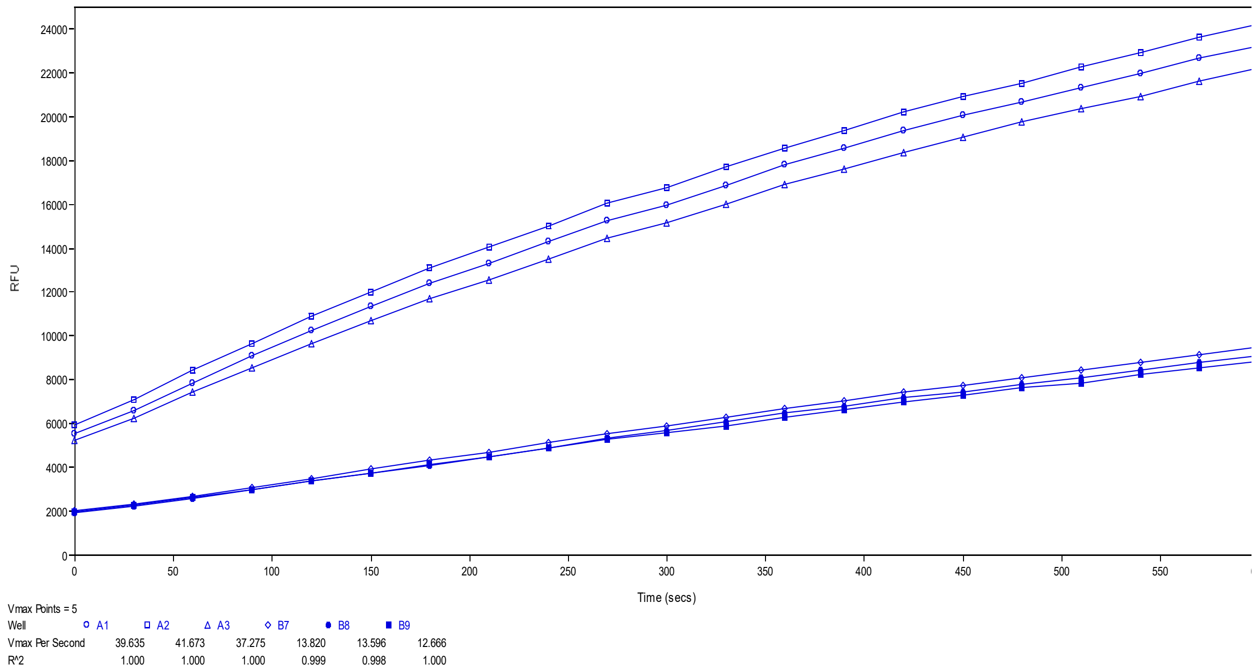

Uninhibited β-Gal activity (top curves) and inhibited β-Gal with 10 μM tPEP-1-11 (bottom curves).

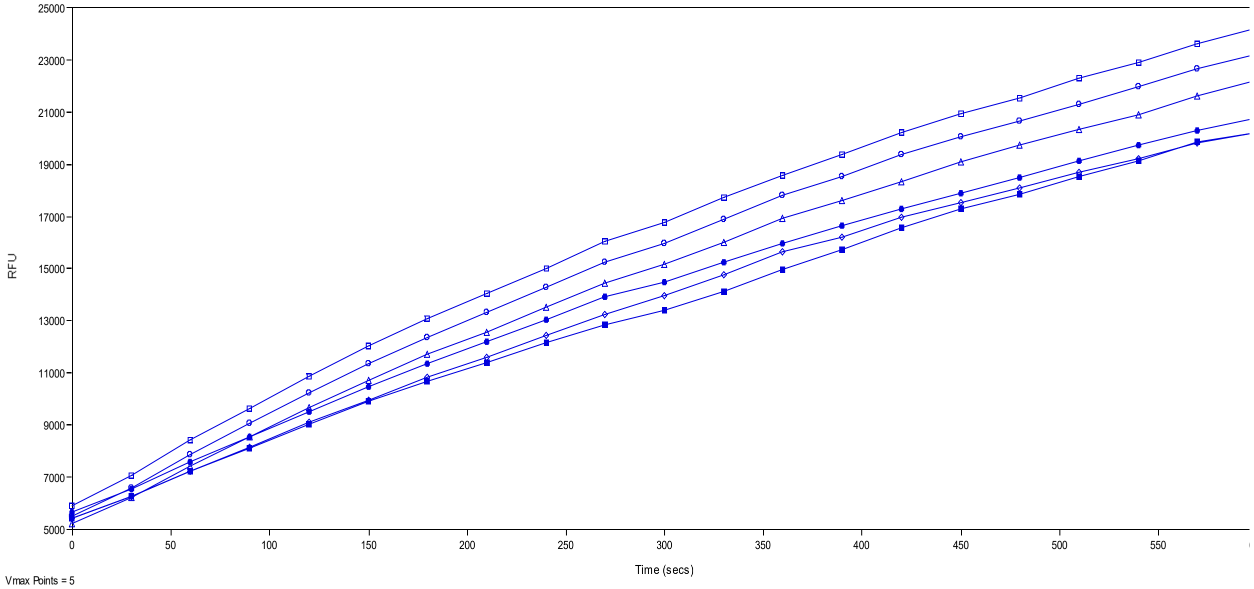

Vmax Points = 5

Well	A1	A2	A3	B10	B11	B12
Vmax Per Second	39.635	41.673	37.275	30.999	32.490	30.420
R^2	1.000	1.000	1.000	1.000	1.000	1.000

Uninhibited β-Gal activity (top curves) and inhibited β-Gal with 10 μM tPEP-1-12 (bottom curves)
