# Supplementary material for: Computational and experimental analysis of short peptide motifs for enzyme inhibition
Source: PLoS One. 2017 Aug 15;12(8):e0182847. doi: 10.1371/journal.pone.0182847 (PMC5557489; doi:10.1371/journal.pone.0182847)
Supplement: S3 Fig — (PDF) [file pone.0182847.s004.pdf]

S3 Fig. Truncation analysis of PEP-2. 50 µM peptide was incubated with 300 µg/L β-Gal and 100 µM RBG in 10 mM potassium phosphate buffer with 0.1 mM $MgCl_2$ (pH 7.4), 25 °C.

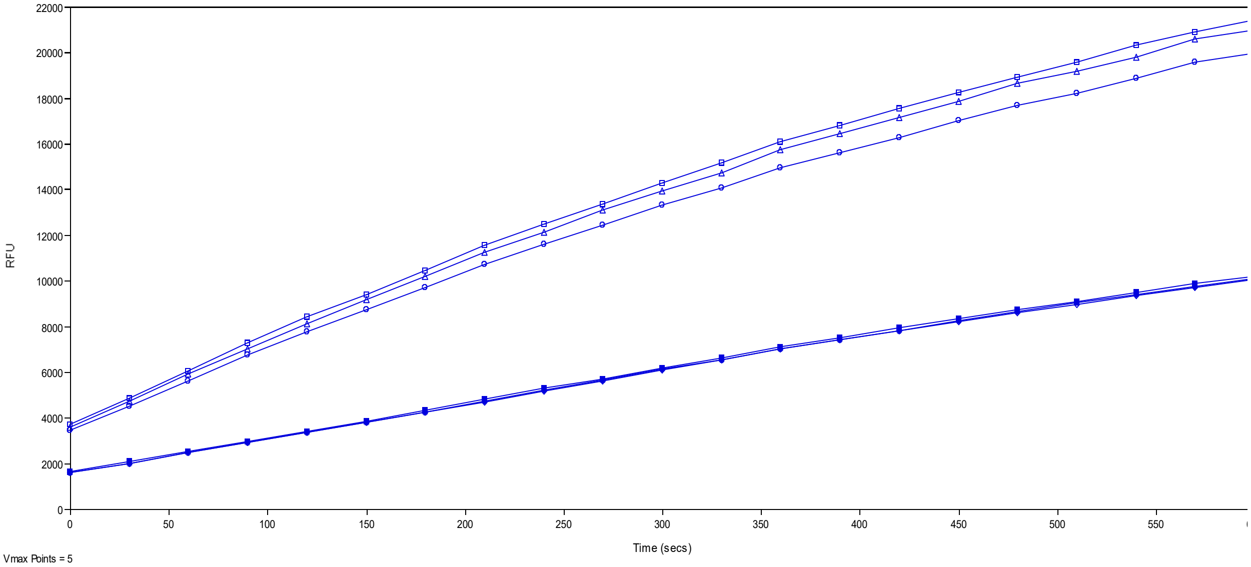

Vmax Points = 5						
Well	A1	A2	A3	A7	A8	A9
Vmax Per Second	36.244	39.508	37.810	15.532	15.493	15.588
R^2	1.000	1.000	1.000	1.000	1.000	1.000

Uninhibited β-Gal activity (top curves) and inhibited β-Gal with 50 µM tPEP-2-1 (bottom curves).

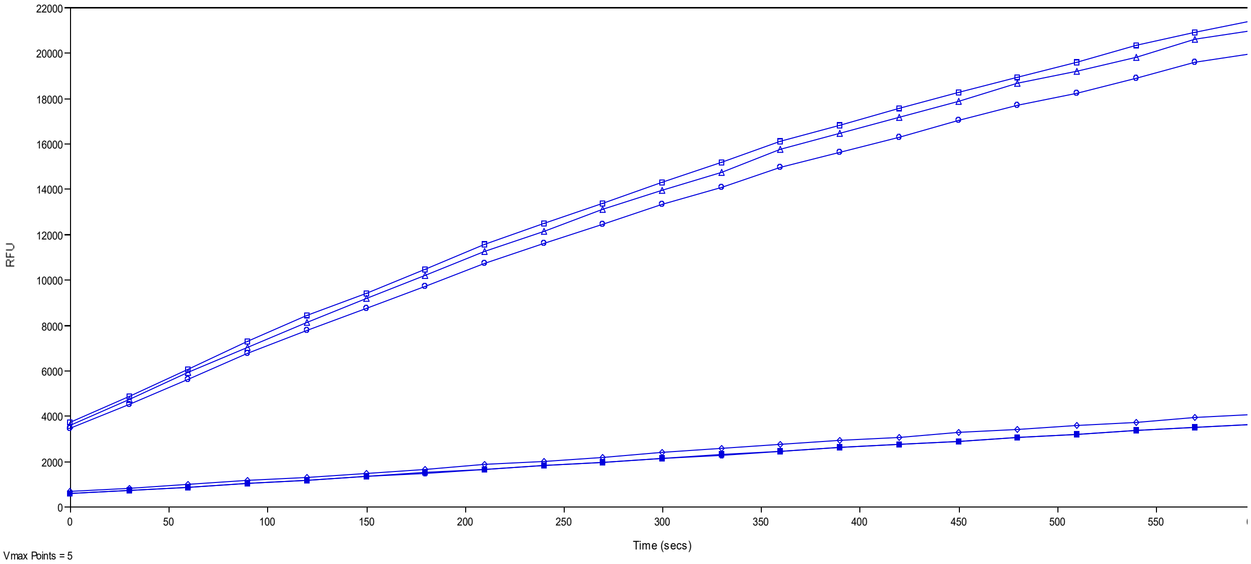

Vmax Points = 5						
Well	A1	A2	A3	A10	A11	A12
Vmax Per Second	36.244	39.508	37.810	6.095	5.449	5.374
R^2	1.000	1.000	1.000	1.000	1.000	1.000

Uninhibited β-Gal activity (top curves) and inhibited β-Gal with 50 µM tPEP-2-2 (bottom curves).

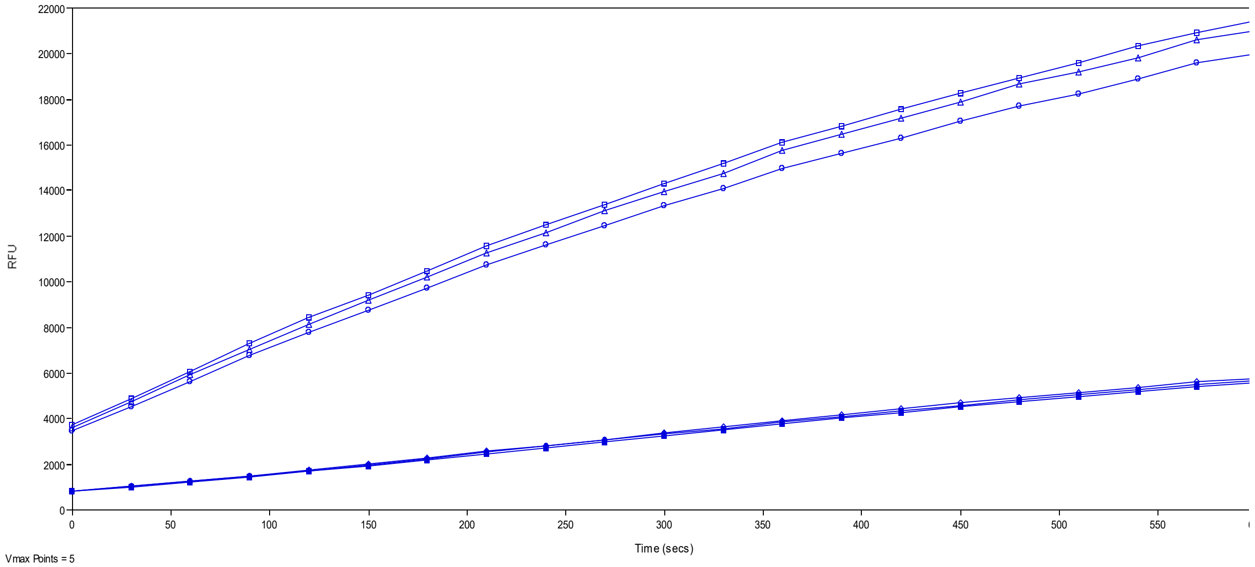

Vmax Points = 5

Well	A1	A2	A3	B4	B5	B6
Vmax Per Second	36.244	39.508	37.810	9.124	9.044	8.813
R^2	1.000	1.000	1.000	0.999	1.000	1.000

Uninhibited β-Gal activity (top curves) and inhibited β-Gal with 50 µM tPEP-2-3 (bottom curves).

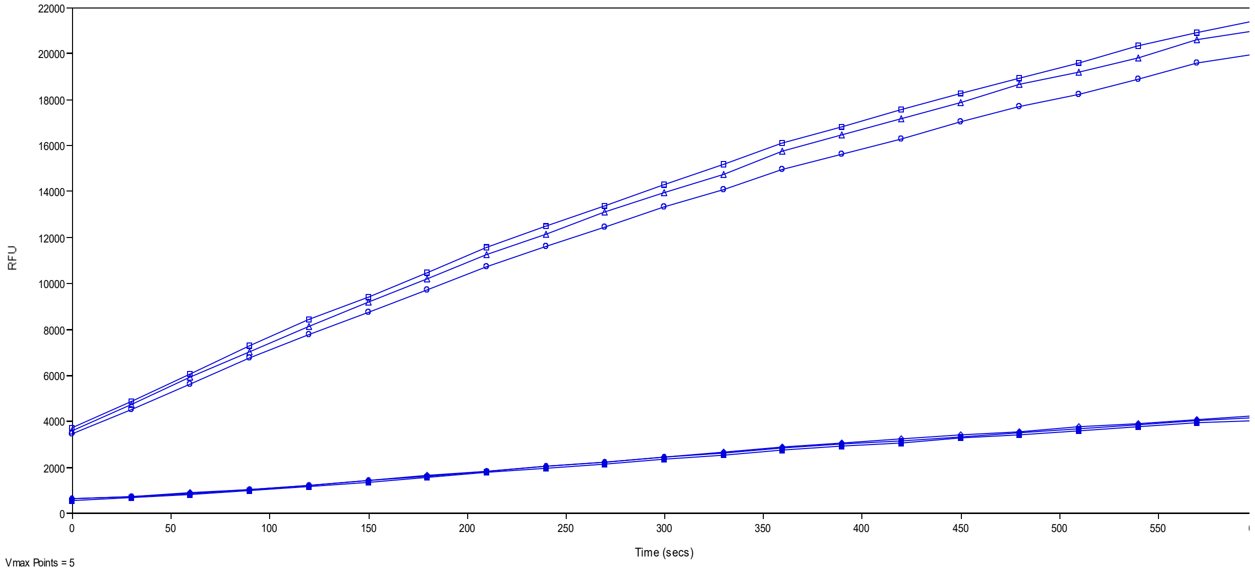

Vmax Points = 5

Well	A1	A2	A3	B7	B8	B9
Vmax Per Second	36.244	39.508	37.810	6.945	6.837	6.854
R^2	1.000	1.000	1.000	1.000	1.000	0.999

Uninhibited β-Gal activity (top curves) and inhibited β-Gal with 50 µM tPEP-2-4 (bottom curves).

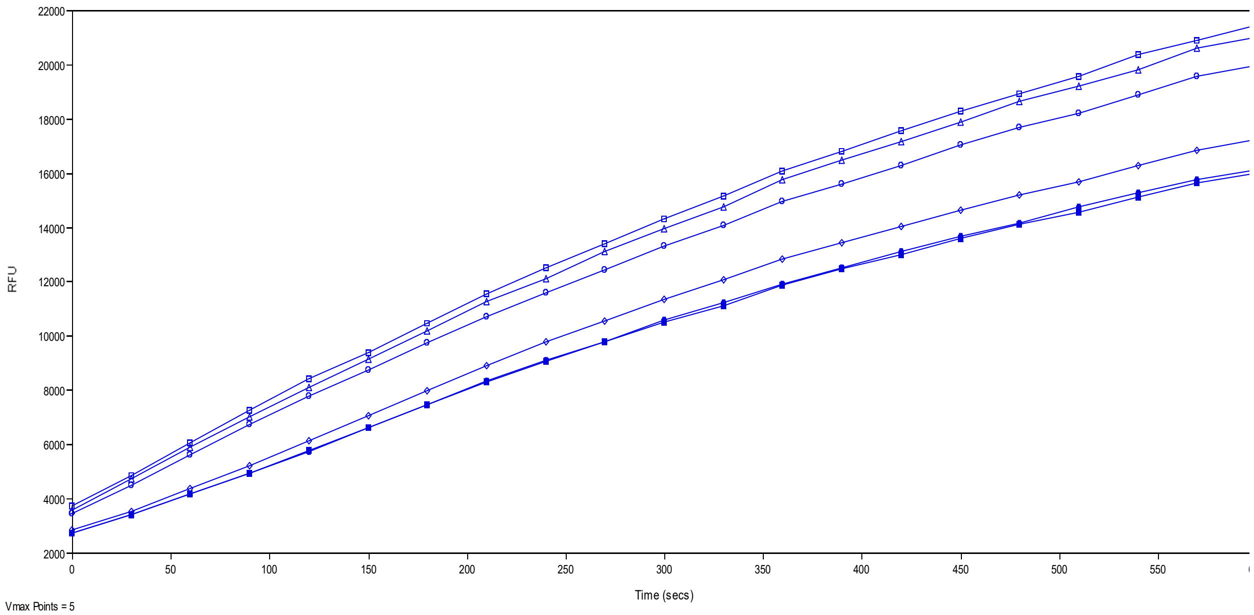

Vmax Points = 5

Well	A1	A2	A3	B10	B11	B12
Vmax Per Second	36.244	39.508	37.810	30.873	28.225	28.204
R^2	1.000	1.000	1.000	1.000	1.000	1.000

Uninhibited β-Gal activity (top curves) and inhibited β-Gal with 50 µM tPEP-2-5 (bottom curves).

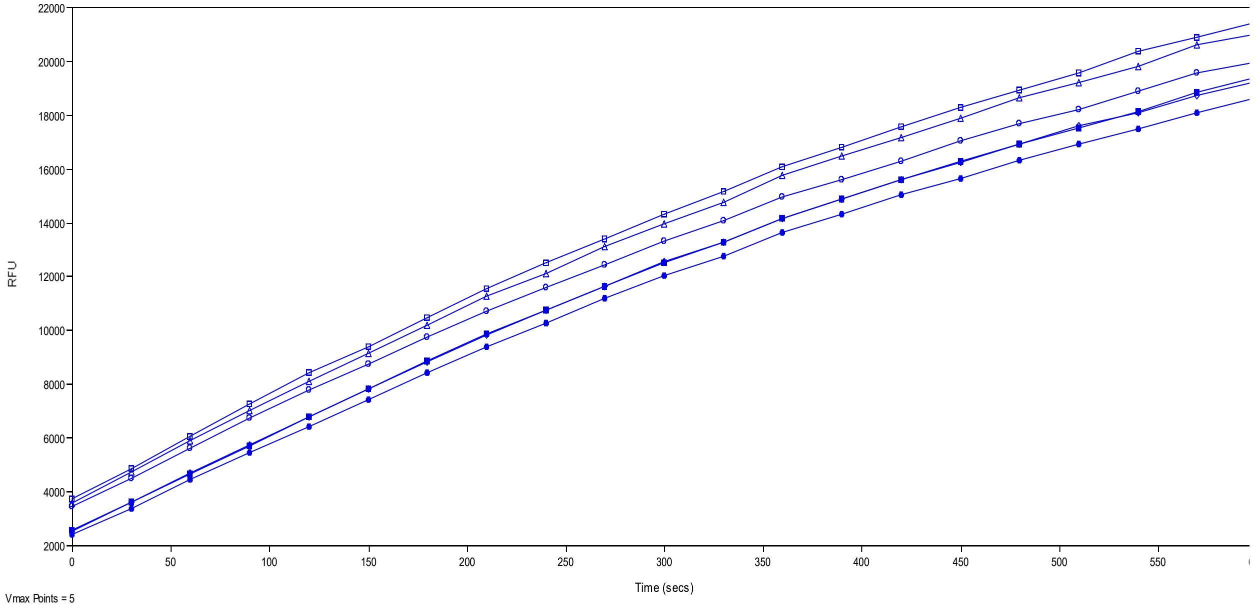

Vmax Points = 5

Well	A1	A2	A3	C4	C5	C6
Vmax Per Second	36.244	39.508	37.810	35.397	33.695	35.081
R^2	1.000	1.000	1.000	1.000	1.000	1.000

Uninhibited β-Gal activity (top curves) and inhibited β-Gal with 50 μM tPEP-2-6 (bottom curves).

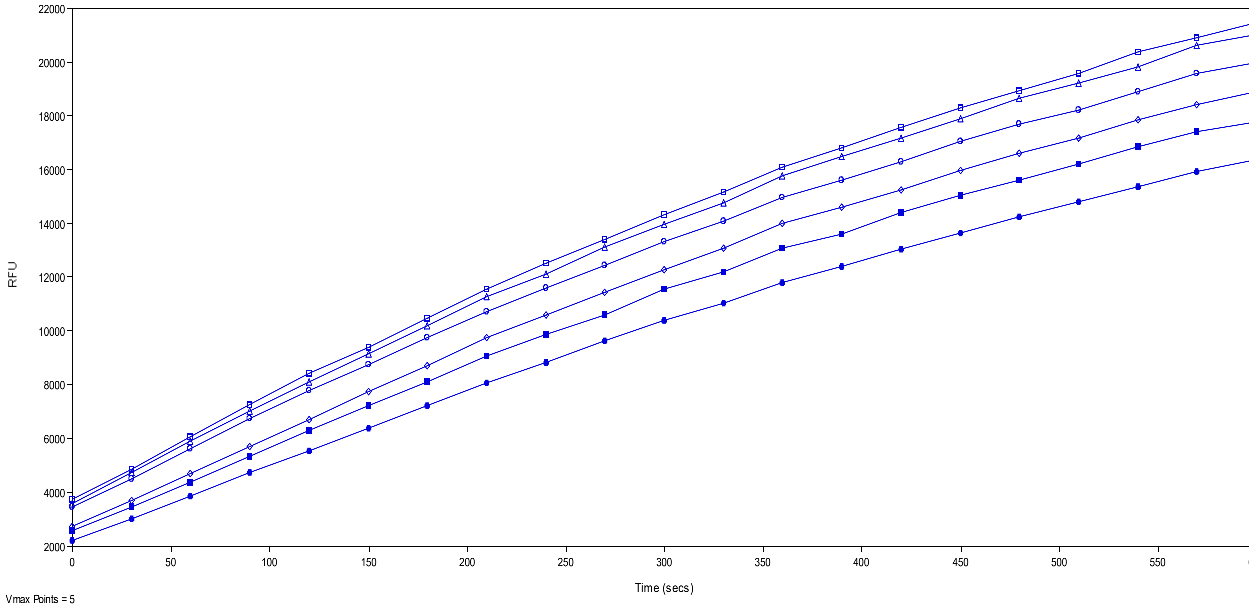

Vmax Points = 5

Well	A1	A2	A3	C7	C8	C9
Vmax Per Second	36.244	39.508	37.810	33.588	28.296	31.502
R^2	1.000	1.000	1.000	1.000	1.000	1.000

Uninhibited β-Gal activity (top curves) and inhibited β-Gal with 50 μM tPEP-2-7 (bottom curves).

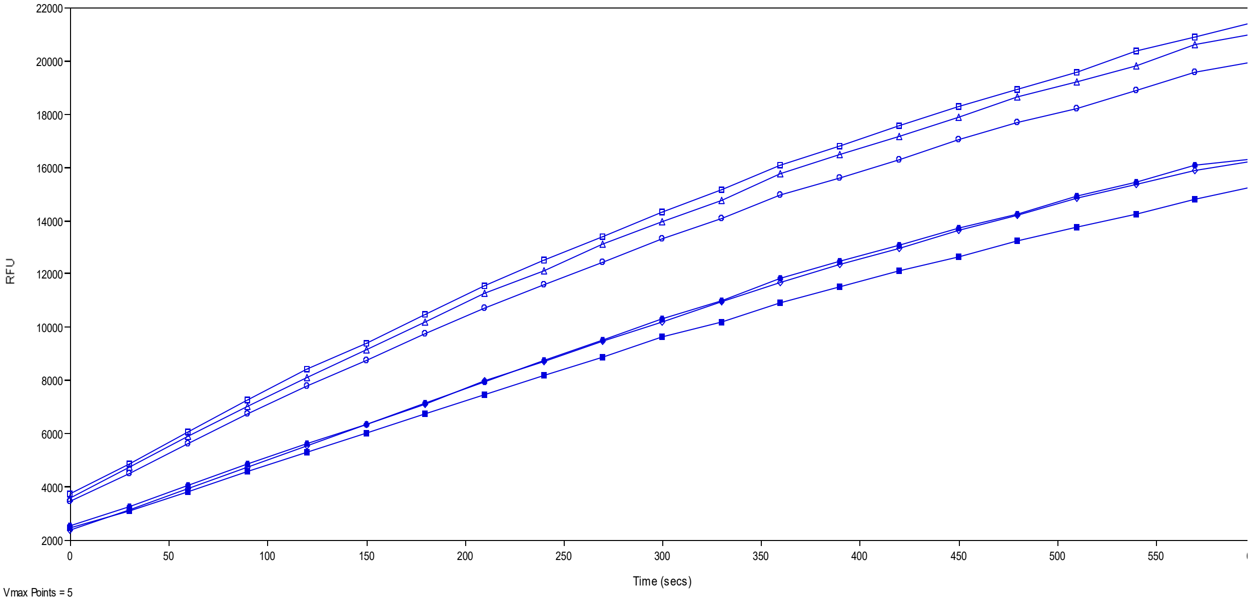

Vmax Points = 5

Well	A1	A2	A3	C10	C11	C12
Vmax Per Second	36.244	39.508	37.810	26.908	26.277	24.390
R^2	1.000	1.000	1.000	1.000	1.000	1.000

Uninhibited β-Gal activity (top curves) and inhibited β-Gal with 50 µM tPEP-2-8 (bottom curves).

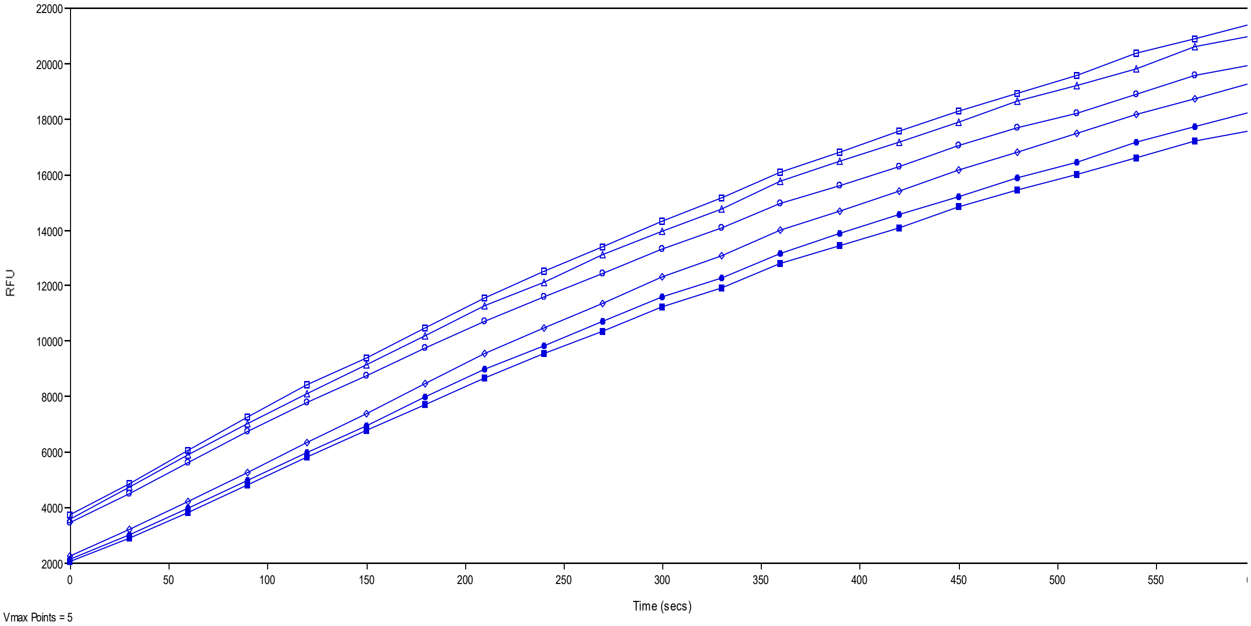

Vmax Points = 5

Well	A1	A2	A3	D4	D5	D6
Vmax Per Second	36.244	39.508	37.810	35.516	33.422	32.400
R^2	1.000	1.000	1.000	1.000	1.000	1.000

Uninhibited β-Gal activity (top curves) and inhibited β-Gal with 50 μM tPEP-2-9 (bottom curves).

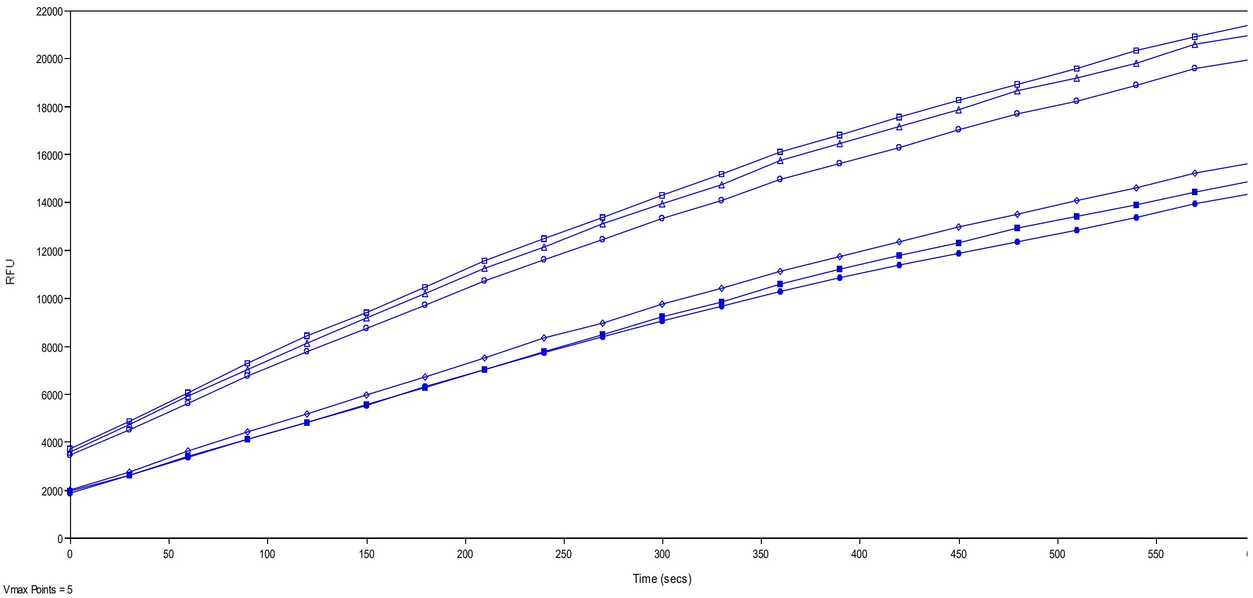

Vmax Points = 5

Well	A1	A2	A3	D7	D8	D9
Vmax Per Second	36.244	39.508	37.810	26.472	24.840	24.557
R^2	1.000	1.000	1.000	1.000	1.000	1.000

Uninhibited β-Gal activity (top curves) and inhibited β-Gal with 50 μM tPEP-2-10 (bottom curves).

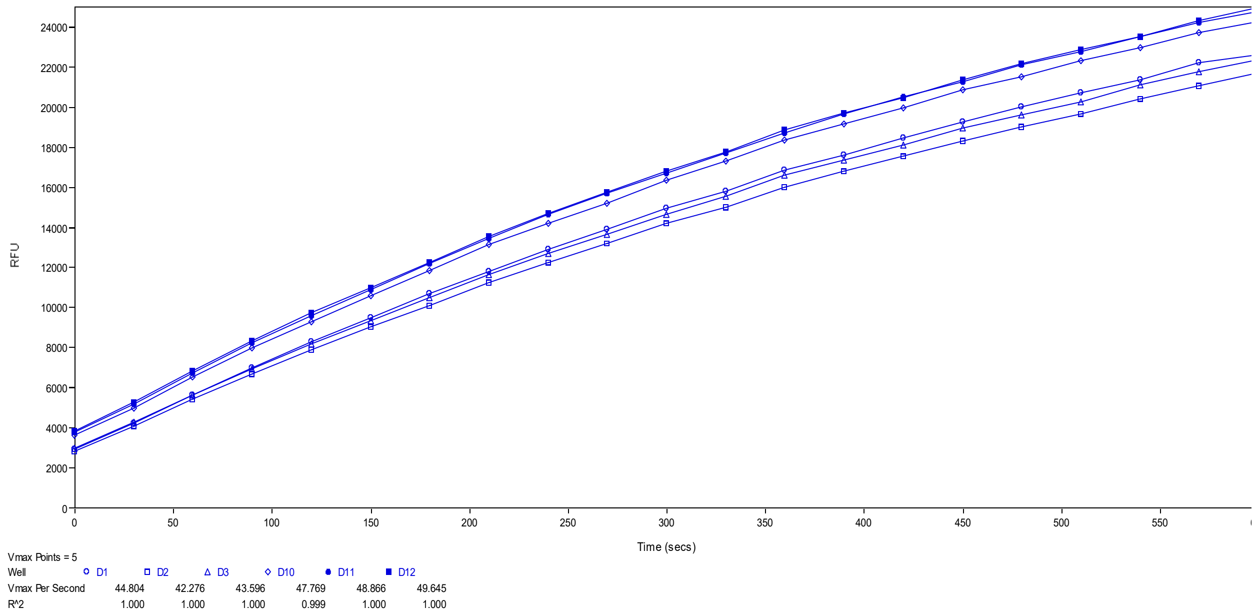

Uninhibited β-Gal activity (top curves) and inhibited β-Gal with 50 μM tPEP-2-11 (bottom curves).

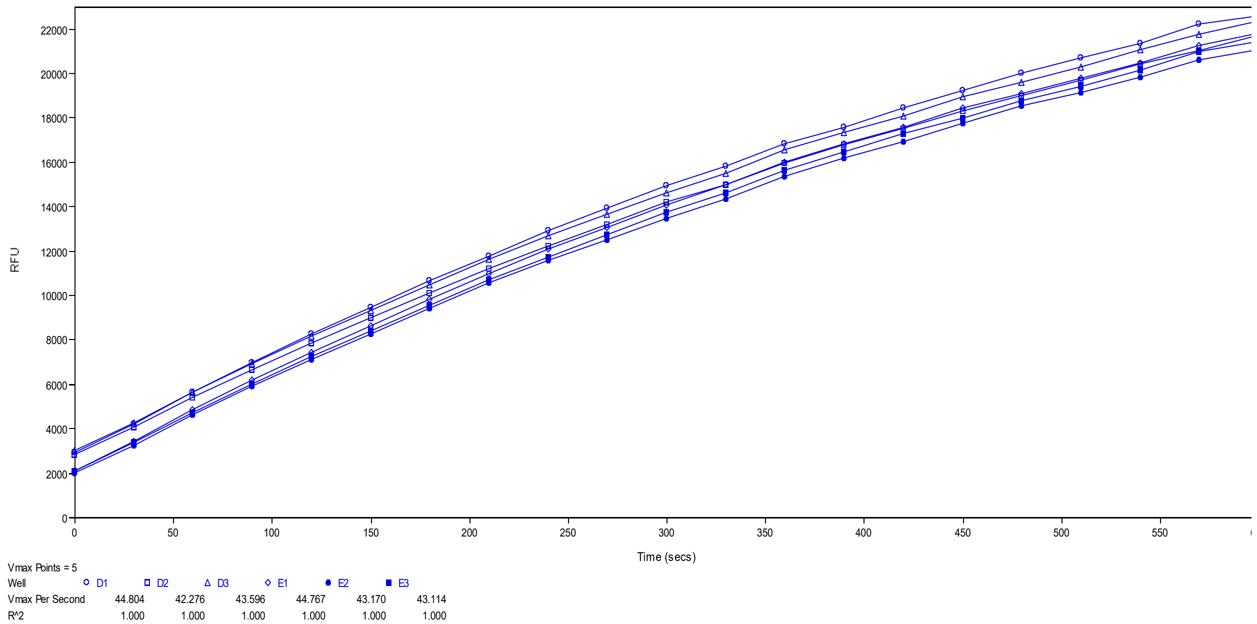

Uninhibited β-Gal activity (top curves) and inhibited β-Gal with 50 μM tPEP-2-12 (bottom curves).
