# Supplementary material for: Computational and experimental analysis of short peptide motifs for enzyme inhibition
Source: PLoS One. 2017 Aug 15;12(8):e0182847. doi: 10.1371/journal.pone.0182847 (PMC5557489; doi:10.1371/journal.pone.0182847)
Supplement: S4 Fig — (PDF) [file pone.0182847.s005.pdf]

S4 Fig. Alanine Scan of PEP-1. 10 µM peptide was incubated with 300 µg/L β-Gal and 100 µM RBG in 10 mM potassium phosphate buffer with 0.1 mM $MgCl_2$ (pH 7.4), 25 °C.

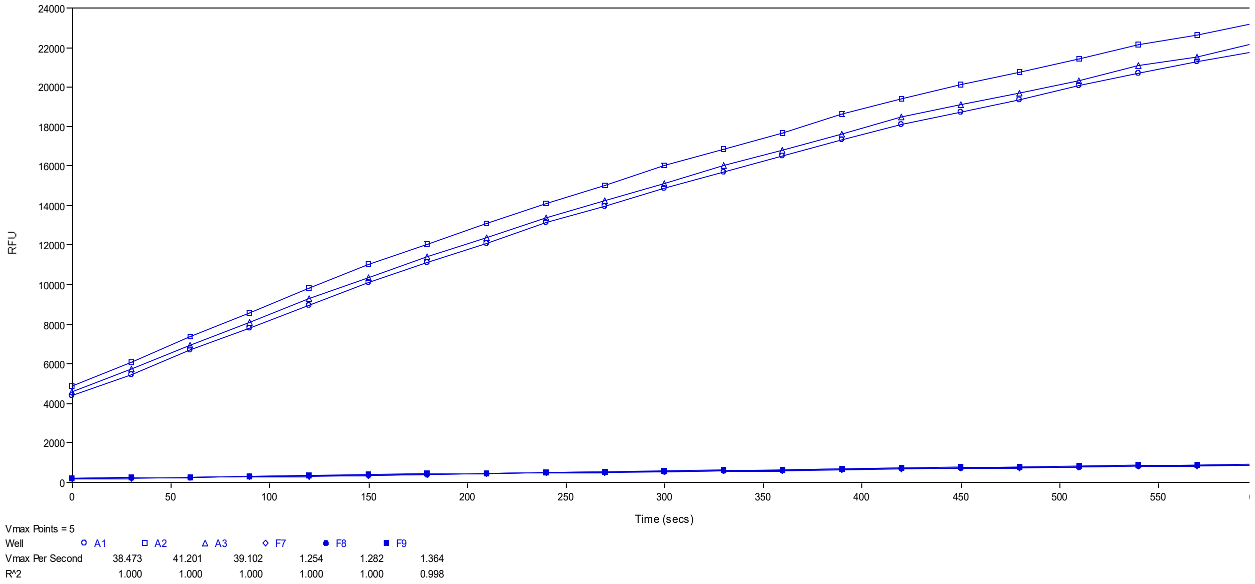

Uninhibited β-Gal activity (top curves) and inhibited β-Gal with 10 µM PEP-1 (bottom curves).

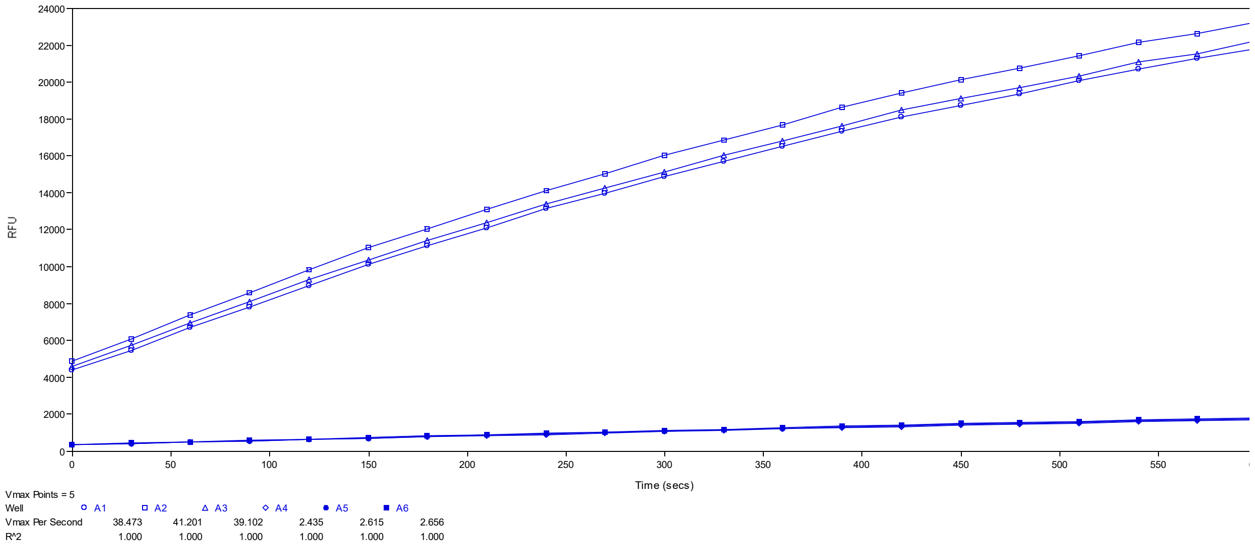

Uninhibited β-Gal activity (top curves) and inhibited β-Gal with 10 µM aPEP-1-1 (bottom curves).

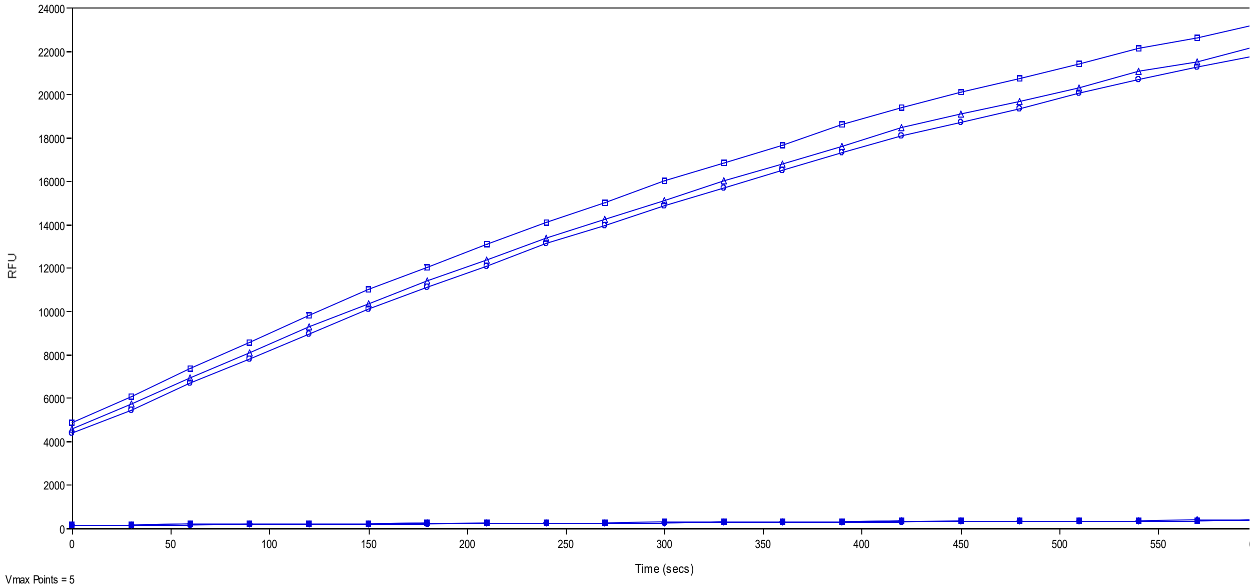

Vmax Points = 5

Well	A1	A2	A3	A7	A8	A9
Vmax Per Second	38.473	41.201	39.102	0.454	0.432	0.414
R^2	1.000	1.000	1.000	0.983	0.999	1.000

Uninhibited β-Gal activity (top curves) and inhibited β-Gal with 10 μM aPEP-1-2 (bottom curves).

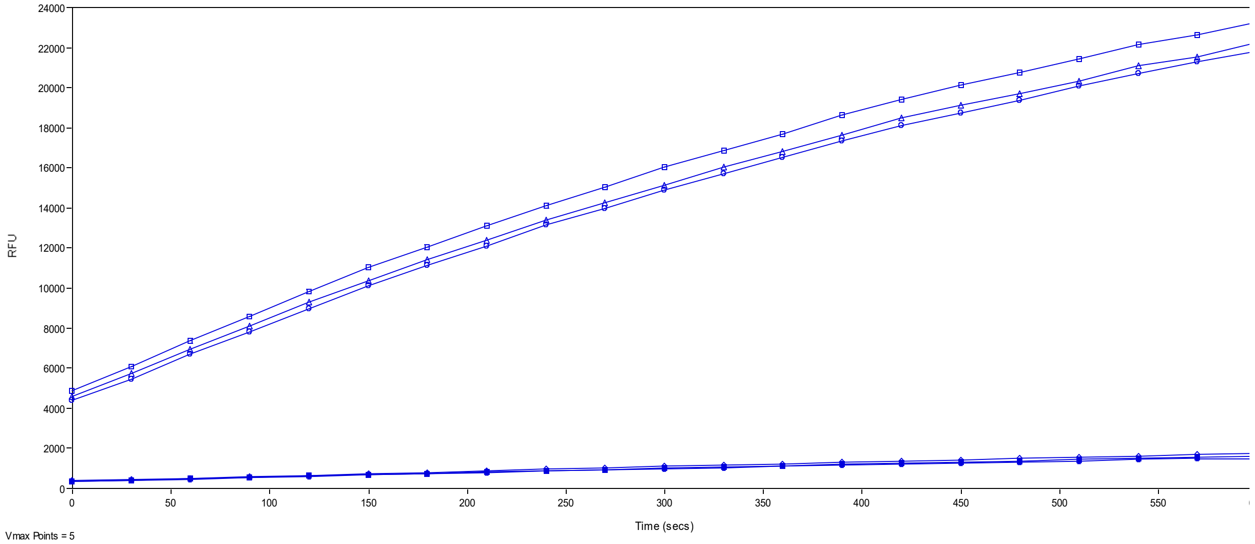

Vmax Points = 5

Well	A1	A2	A3	A10	A11	A12
Vmax Per Second	38.473	41.201	39.102	2.500	2.200	2.272
R^2	1.000	1.000	1.000	0.999	0.999	1.000

Uninhibited β-Gal activity (top curves) and inhibited β-Gal with 10 μM aPEP-1-3 (bottom curves).

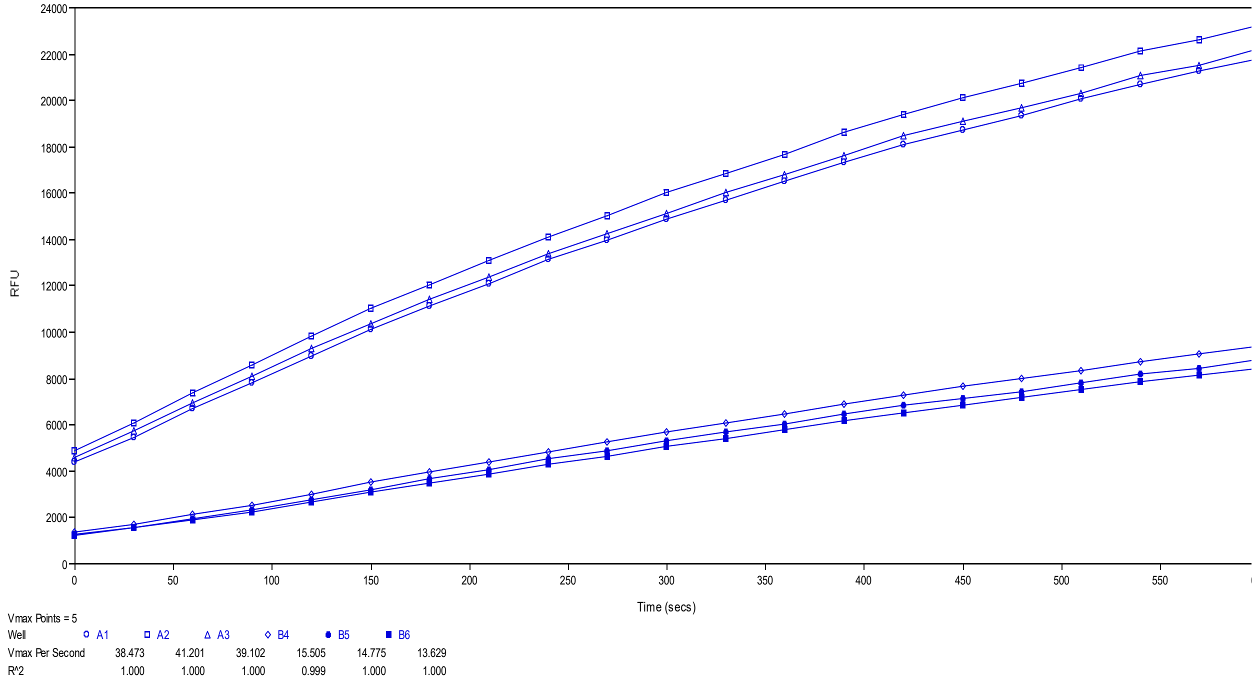

Uninhibited β-Gal activity (top curves) and inhibited β-Gal with 10 μM aPEP-1-4 (bottom curves).

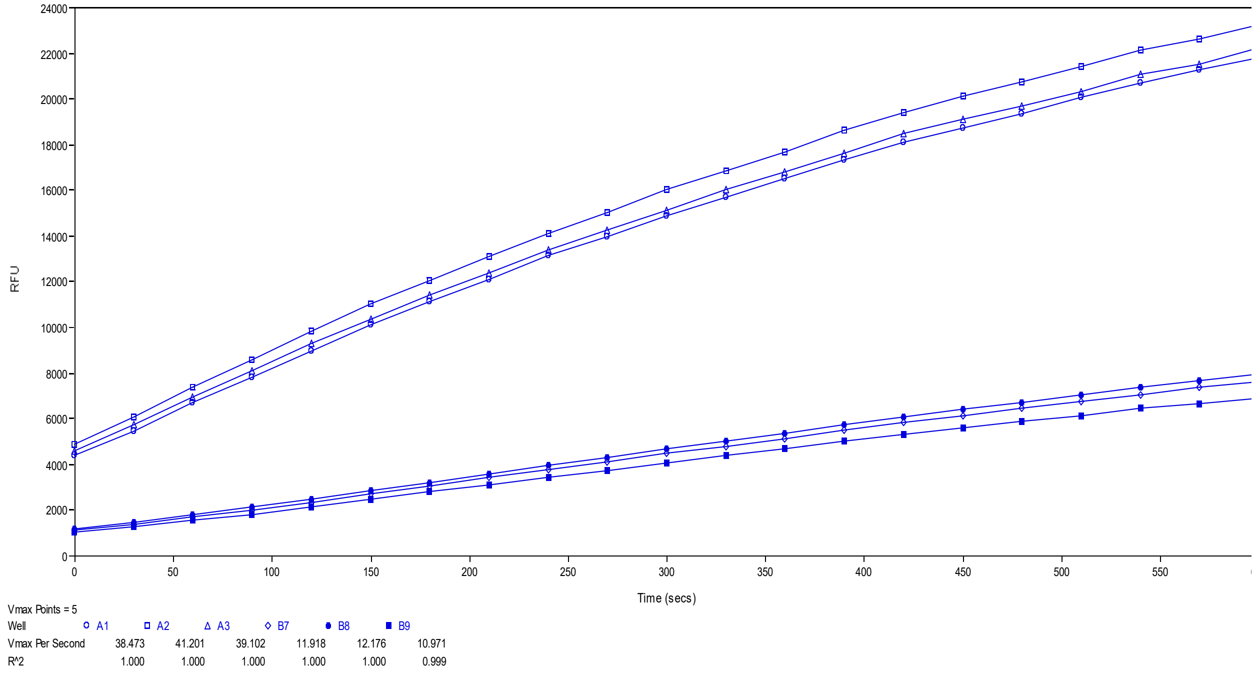

Uninhibited β-Gal activity (top curves) and inhibited β-Gal with 10 μM aPEP-1-5 (bottom curves).

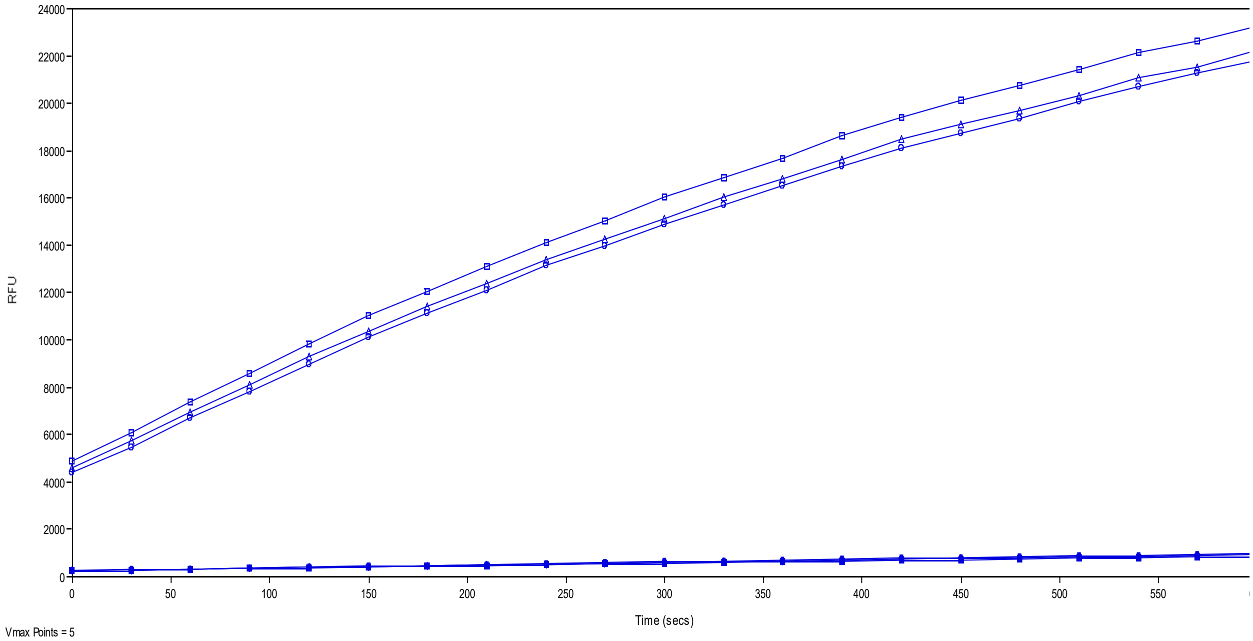

Vmax Points = 5

Well	A1	A2	A3	B10	B11	B12
Vmax Per Second	38.473	41.201	39.102	1.231	1.306	1.127
R^2	1.000	1.000	1.000	0.999	1.000	1.000

Uninhibited β-Gal activity (top curves) and inhibited β-Gal with 10 μM aPEP-1-6 (bottom curves).

.

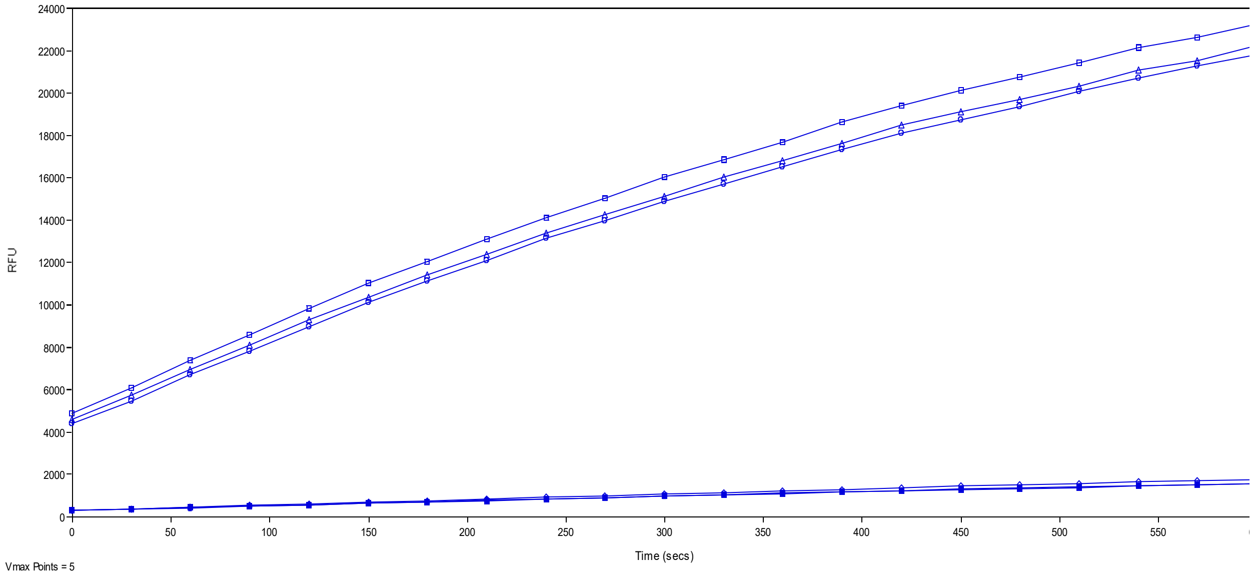

Vmax Points = 5

Well	A1	A2	A3	C4	C5	C6
Vmax Per Second	38.473	41.201	39.102	2.625	2.368	2.334
R^2	1.000	1.000	1.000	1.000	0.999	1.000

Uninhibited β-Gal activity (top curves) and inhibited β-Gal with 10 μM aPEP-1-7 (bottom curves).

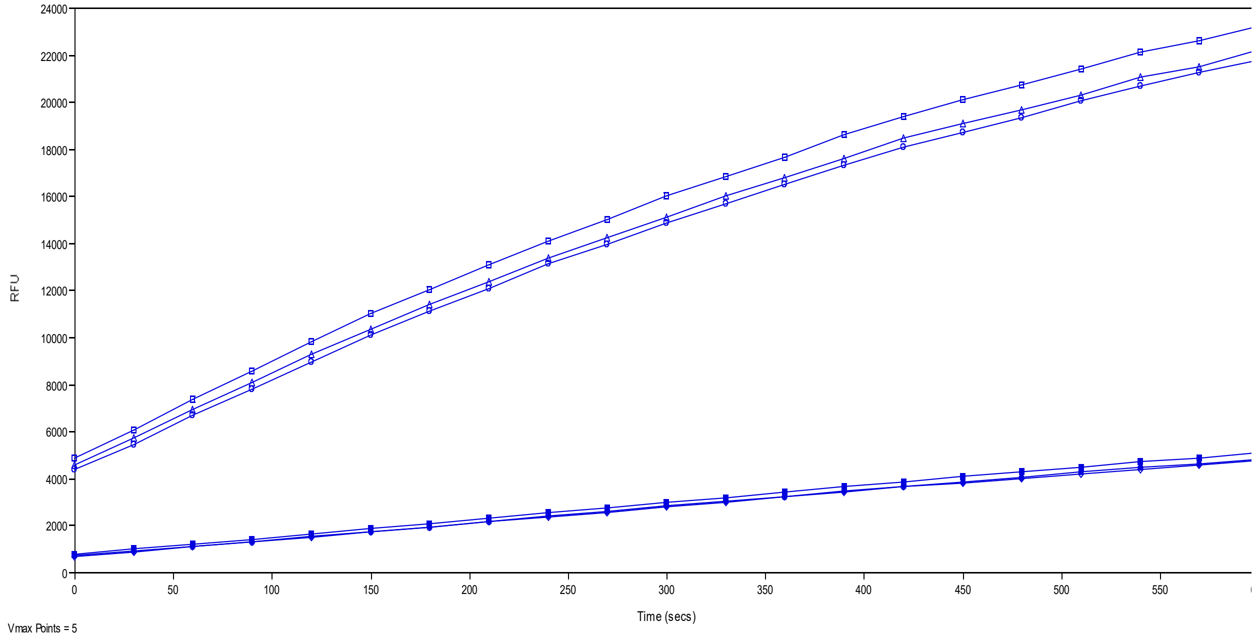

Vmax Points = 5

Well	A1	A2	A3	C7	C8	C9
Vmax Per Second	38.473	41.201	39.102	7.156	7.321	7.475
R^2	1.000	1.000	1.000	1.000	1.000	1.000

Uninhibited β-Gal activity (top curves) and inhibited β-Gal with 10 μM aPEP-1-8 (bottom curves).

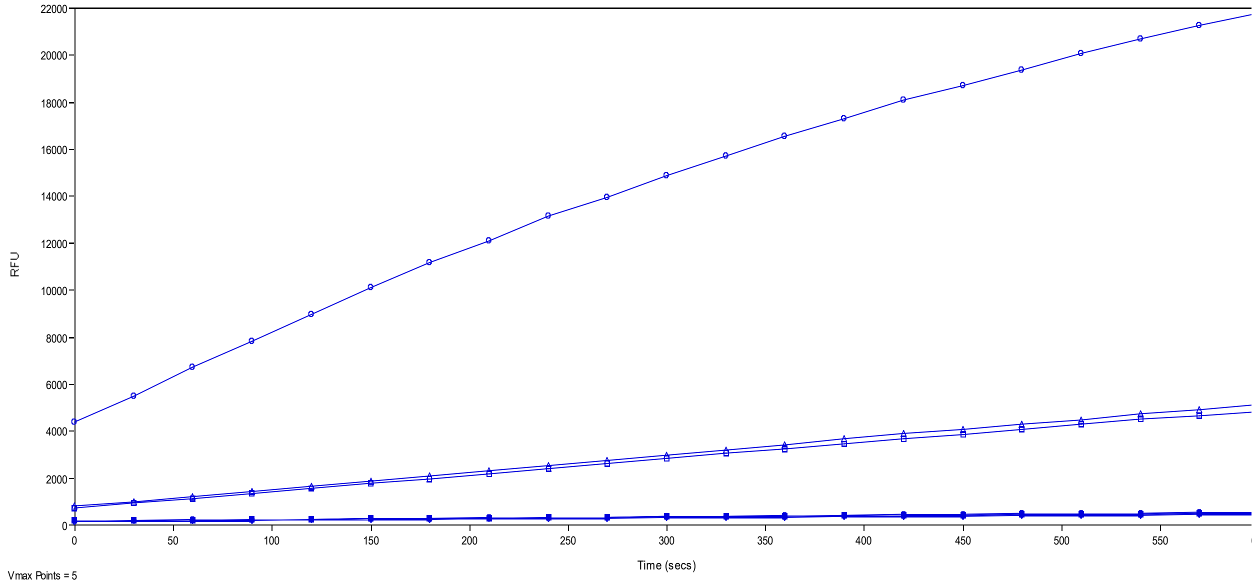

Vmax Points = 5

Well	A1	C8	C9	C10	C11	C12
Vmax Per Second	38.473	7.321	7.475	0.525	0.672	0.585
R^2	1.000	1.000	1.000	0.993	0.995	0.999

Uninhibited β-Gal activity (top curves) and inhibited β-Gal with 10 μM aPEP-1-9 (bottom curves).

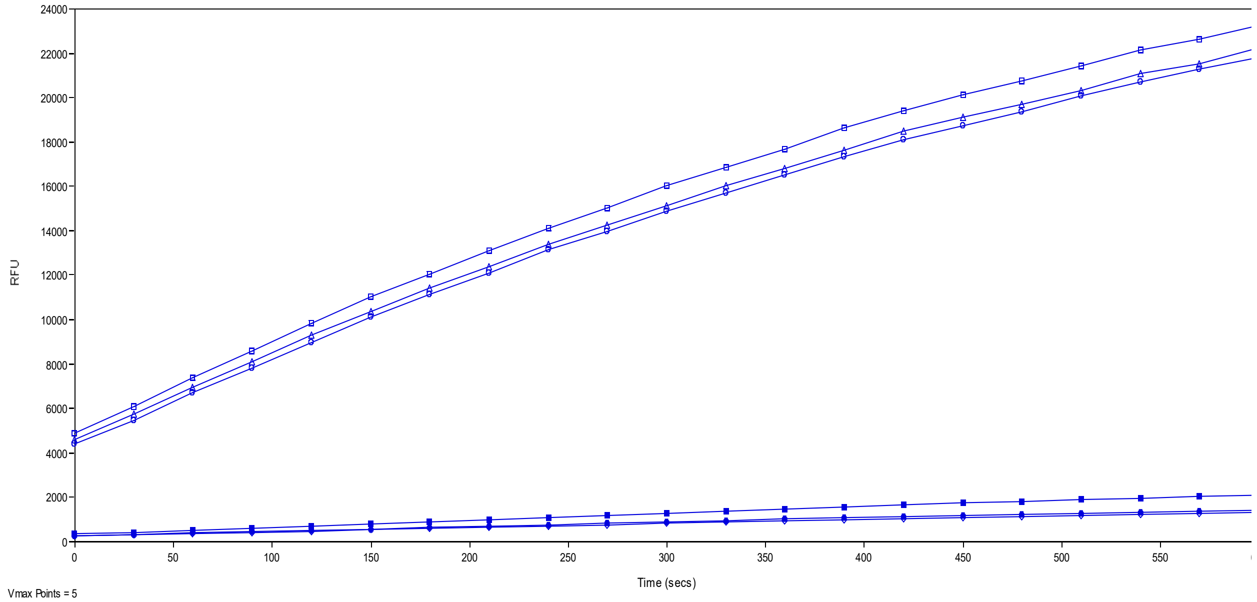

Vmax Points = 5

Well	A1	A2	A3	D4	D5	D6
Vmax Per Second	38.473	41.201	39.102	2.013	2.179	3.286
R^2	1.000	1.000	1.000	0.999	1.000	1.000

Uninhibited β-Gal activity (top curves) and inhibited β-Gal with 10 μM aPEP-1-10 (bottom curves).

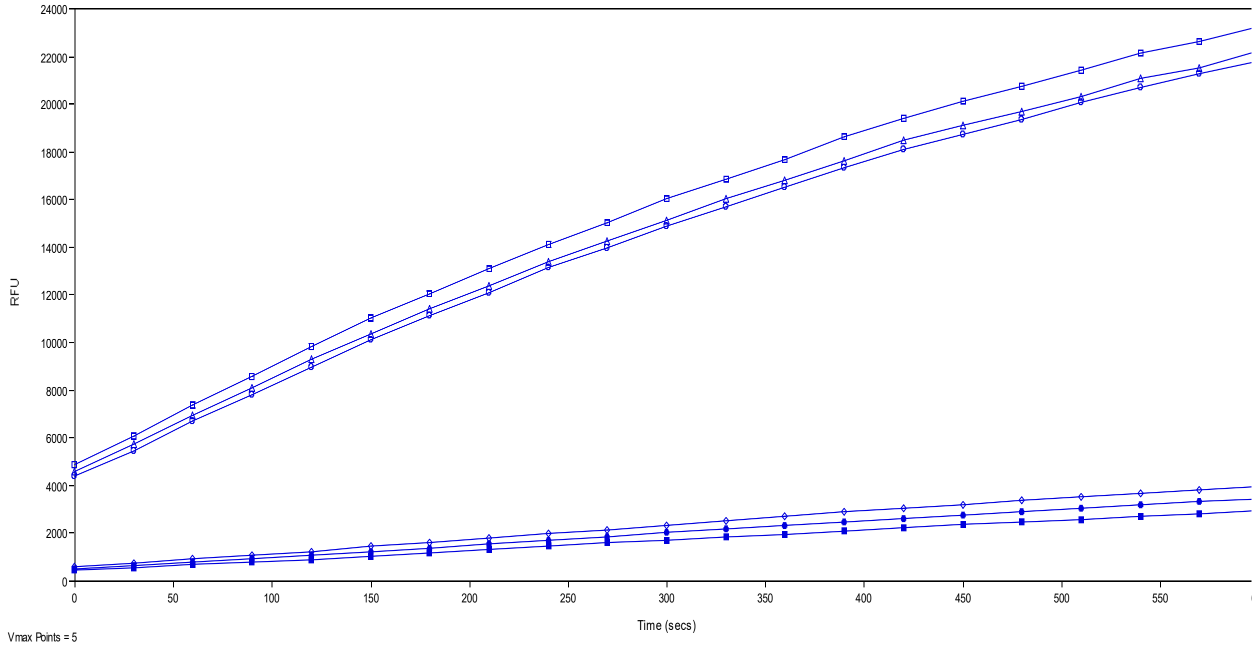

Vmax Points = 5

Well	A1	A2	A3	D7	D8	D9
Vmax Per Second	38.473	41.201	39.102	6.196	5.355	4.557
R^2	1.000	1.000	1.000	1.000	1.000	1.000

Uninhibited β-Gal activity (top curves) and inhibited β-Gal with 10 μM aPEP-1-11 (bottom curves).

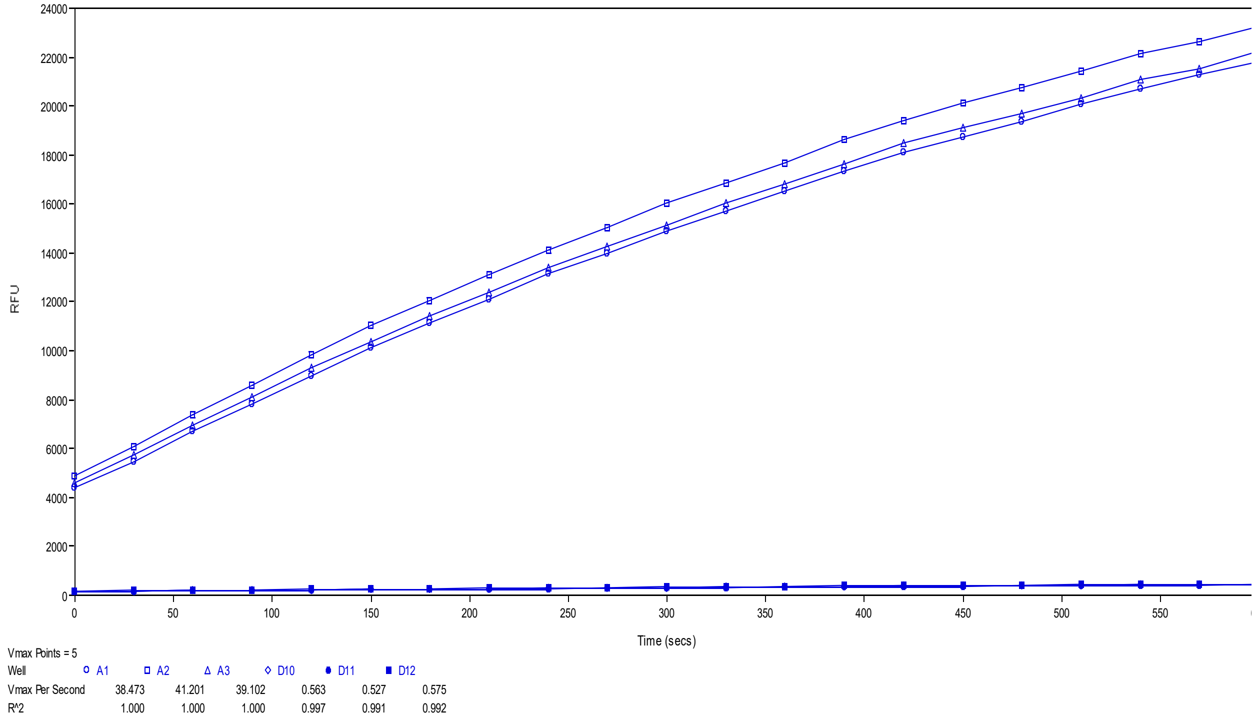

Uninhibited β-Gal activity (top curves) and inhibited β-Gal with 10 μM aPEP-1-12 (bottom curves).

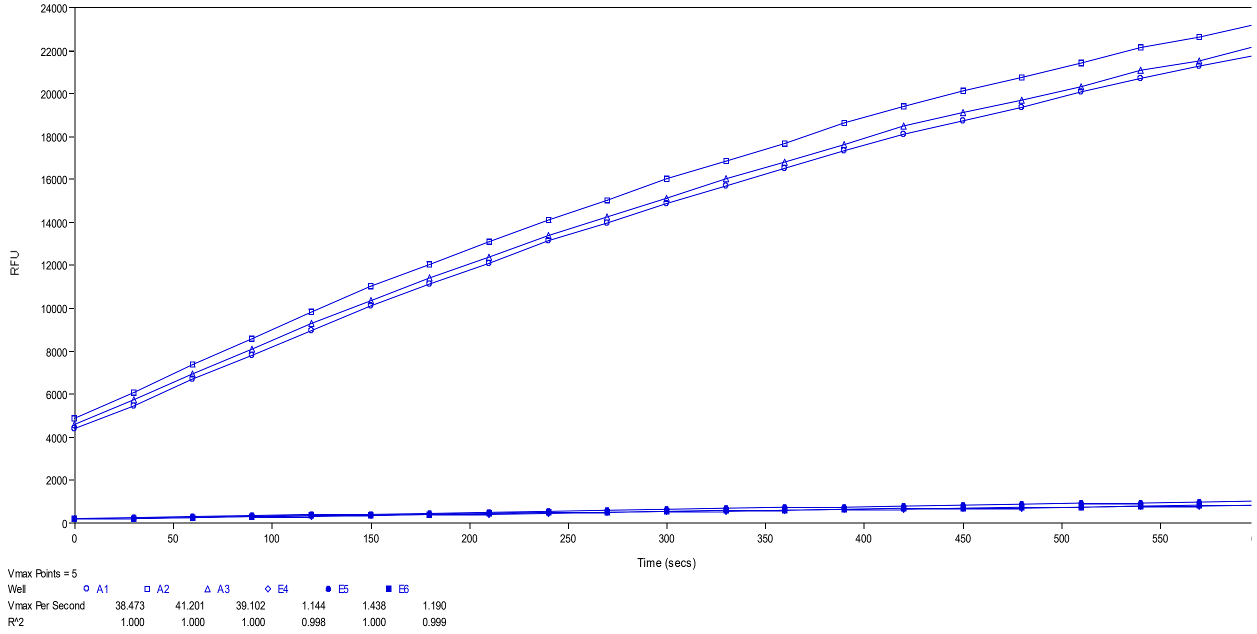

Uninhibited β-Gal activity (top curves) and inhibited β-Gal with 10 μM aPEP-1-13 (bottom curves).

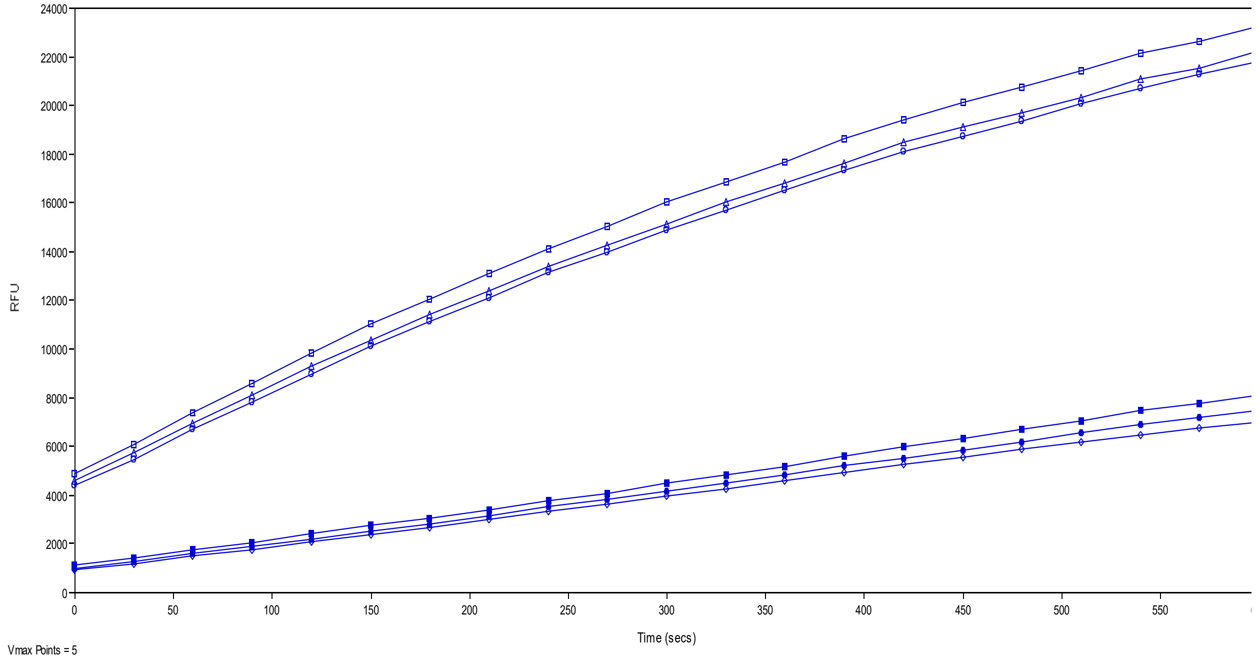

Vmax Points = 5

Well	A1	A2	A3	E7	E8	E9
Vmax Per Second	38.473	41.201	39.102	10.921	11.533	12.785
R^2	1.000	1.000	1.000	0.999	1.000	0.998

Uninhibited β-Gal activity (top curves) and inhibited β-Gal with 10 μM aPEP-1-14 (bottom curves).

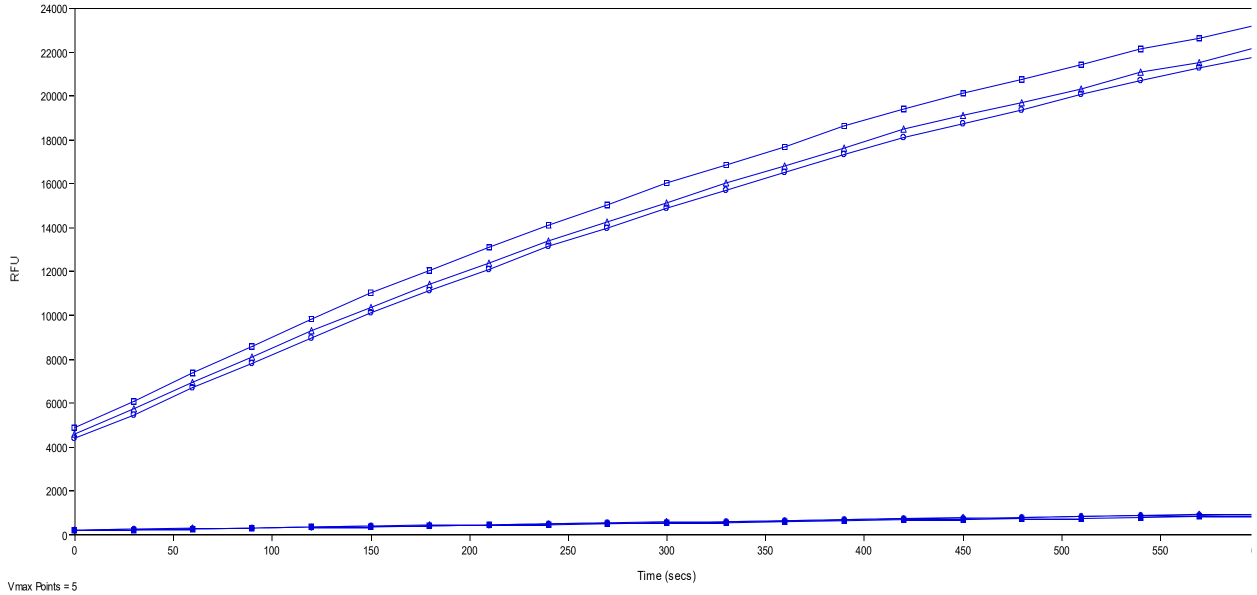

Vmax Points = 5

Well	A1	A2	A3	E10	E11	E12
Vmax Per Second	38.473	41.201	39.102	1.291	1.309	1.171
R^2	1.000	1.000	1.000	0.991	0.999	0.999

Uninhibited β-Gal activity (top curves) and inhibited β-Gal with 10 μM aPEP-1-15 (bottom curves).

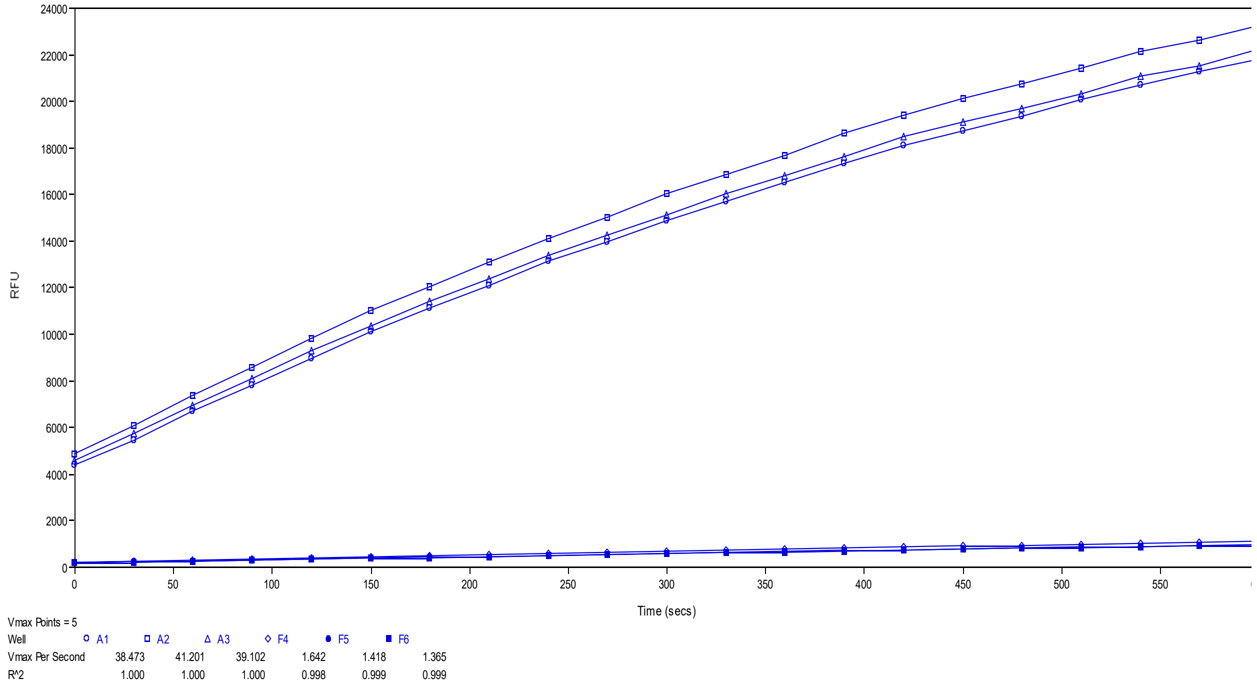

Uninhibited β-Gal activity (top curves) and inhibited β-Gal with 10 μM aPEP-1-16 (bottom curves).

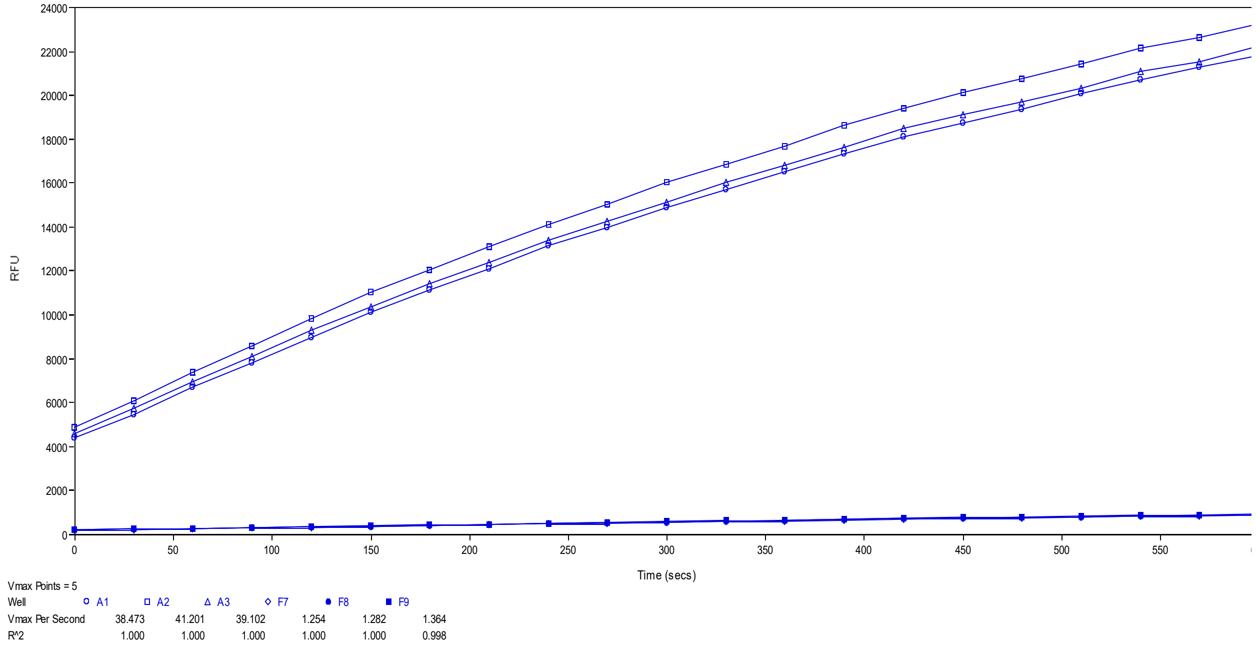

Uninhibited β-Gal activity (top curves) and inhibited β-Gal with 10 μM aPEP-1-17 (bottom curves).
