# Supplementary material for: Computational and experimental analysis of short peptide motifs for enzyme inhibition
Source: PLoS One. 2017 Aug 15;12(8):e0182847. doi: 10.1371/journal.pone.0182847 (PMC5557489; doi:10.1371/journal.pone.0182847)
Supplement: S5 Fig — (PDF) [file pone.0182847.s006.pdf]

S5 Fig. Alanine Scan of PEP-2. 50 μM peptide was incubated with 300 μg/L β-Gal and 100 μM RBG in 10 mM potassium phosphate buffer with 0.1 mM $MgCl_2$ (pH 7.4), 25 °C.

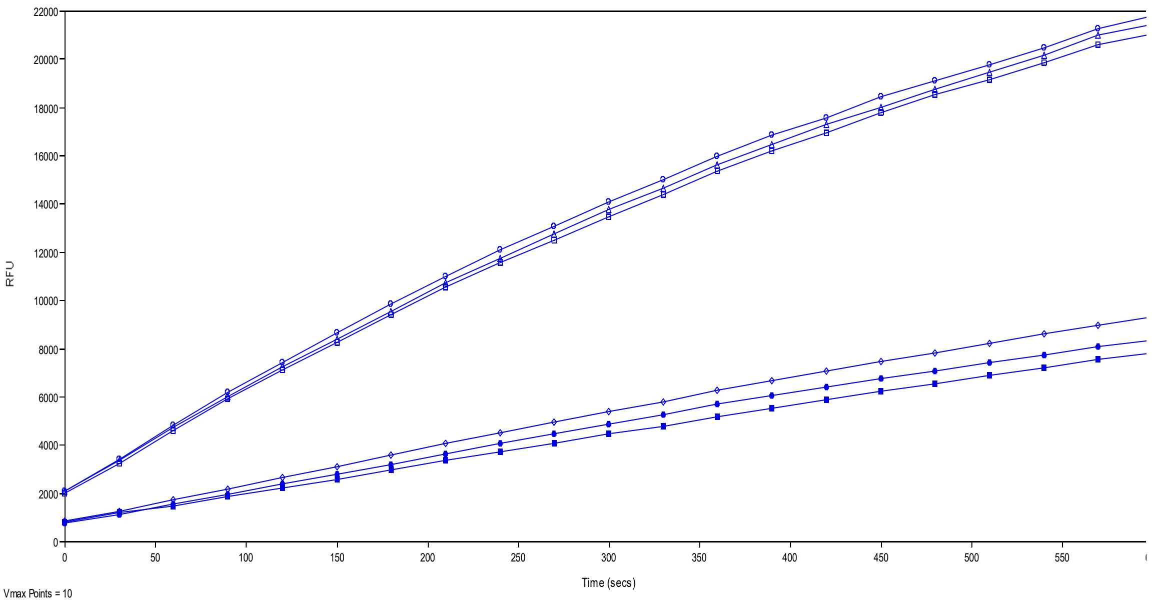

Vmax Points = 10

Well	E1	E2	E3	E7	E8	E9
Vmax Per Second	40.821	39.256	39.634	15.394	13.864	12.389
R^2	0.997	0.997	0.998	1.000	1.000	1.000

Uninhibited β-Gal activity (top curves) and inhibited β-Gal with 50 μM PEP-2 (bottom curves)

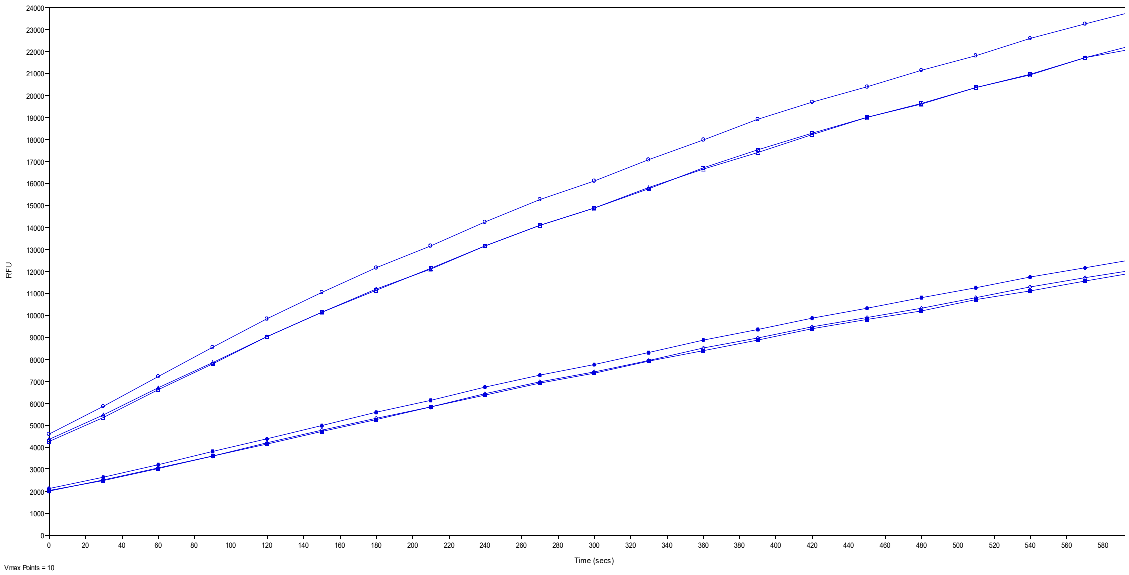

Vmax Points = 10

Well	D1	D2	D3	D4	D5	D6
Vmax Per Second	39.667	36.737	36.339	18.537	19.279	18.355
R^2	0.997	0.998	0.998	1.000	1.000	1.000

Uninhibited β-Gal activity (top curves) and inhibited β-Gal with 50 μM aPEP-2-1 (bottom curves)

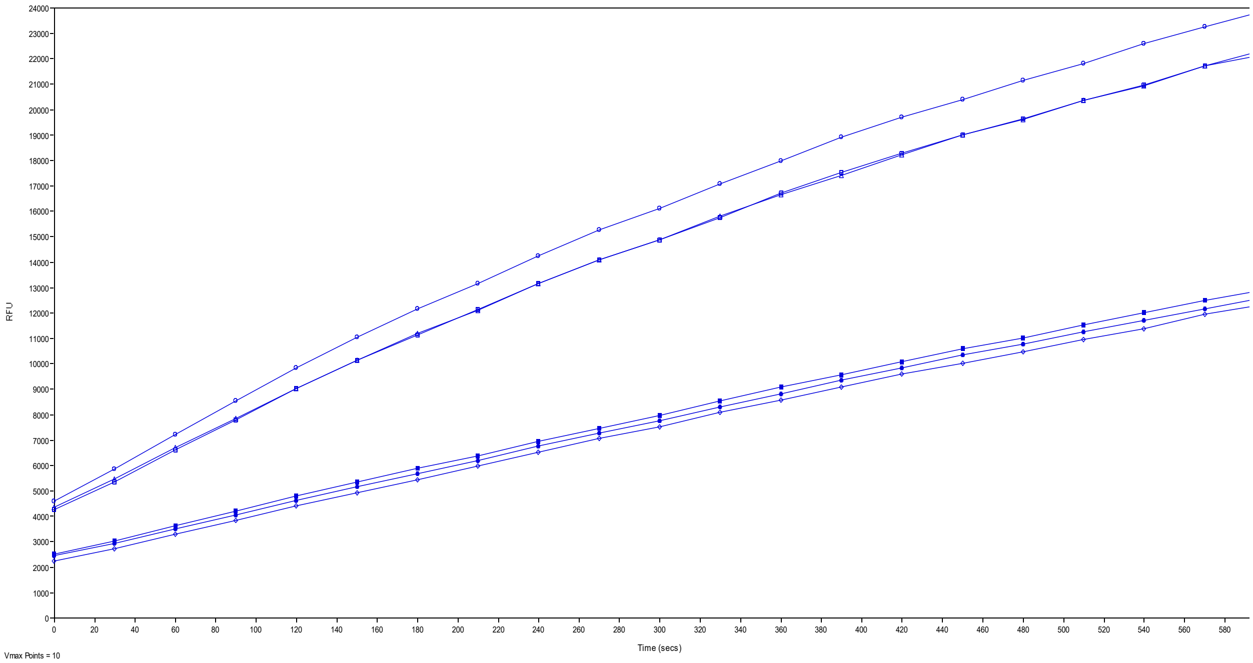

Vmax Points = 10

Well	D1	D2	D3	D7	D8	D9
Vmax Per Second	39.667	36.737	36.339	17.975	17.960	18.410
R^2	0.997	0.998	0.998	1.000	1.000	1.000

Uninhibited β-Gal activity (top curves) and inhibited β-Gal with 50 μM aPEP-2-2 (bottom curves)

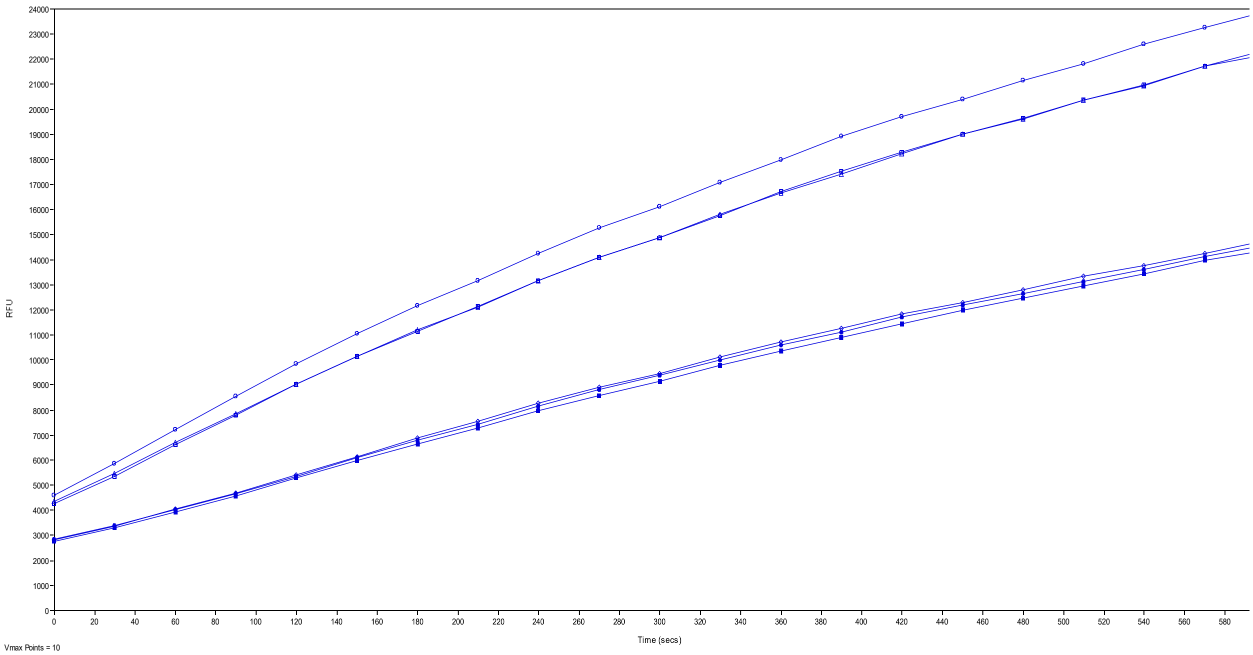

Vmax Points = 10

Well	D1	D2	D3	D10	D11	D12
Vmax Per Second	39.667	36.737	36.339	23.053	22.602	22.004
R^2	0.997	0.998	0.998	0.999	0.999	1.000

Uninhibited β-Gal activity (top curves) and inhibited β-Gal with 50 μM aPEP-2-3 (bottom curves)

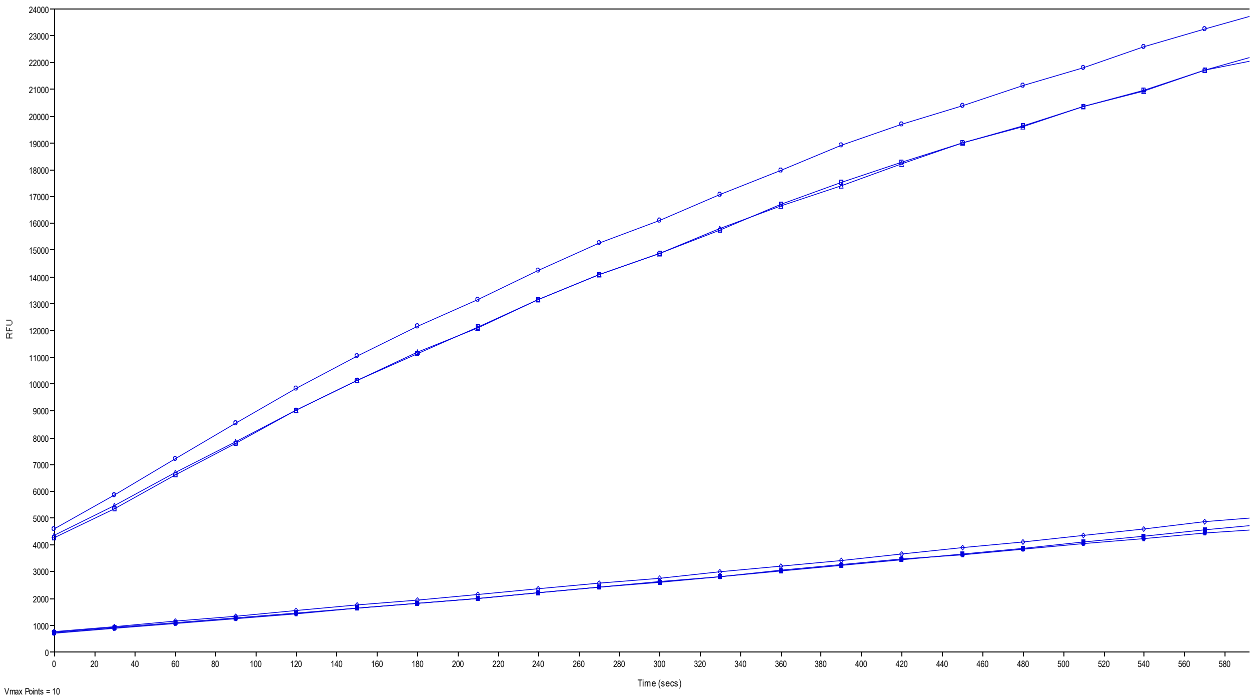

Vmax Points = 10

Well	D1	D2	D3	E4	E5	E6
Vmax Per Second	39.667	36.737	36.339	7.700	6.832	7.263
R^2	0.997	0.998	0.998	0.999	1.000	1.000

Uninhibited β-Gal activity (top curves) and inhibited β-Gal with 50 μM aPEP-2-4 (bottom curves)

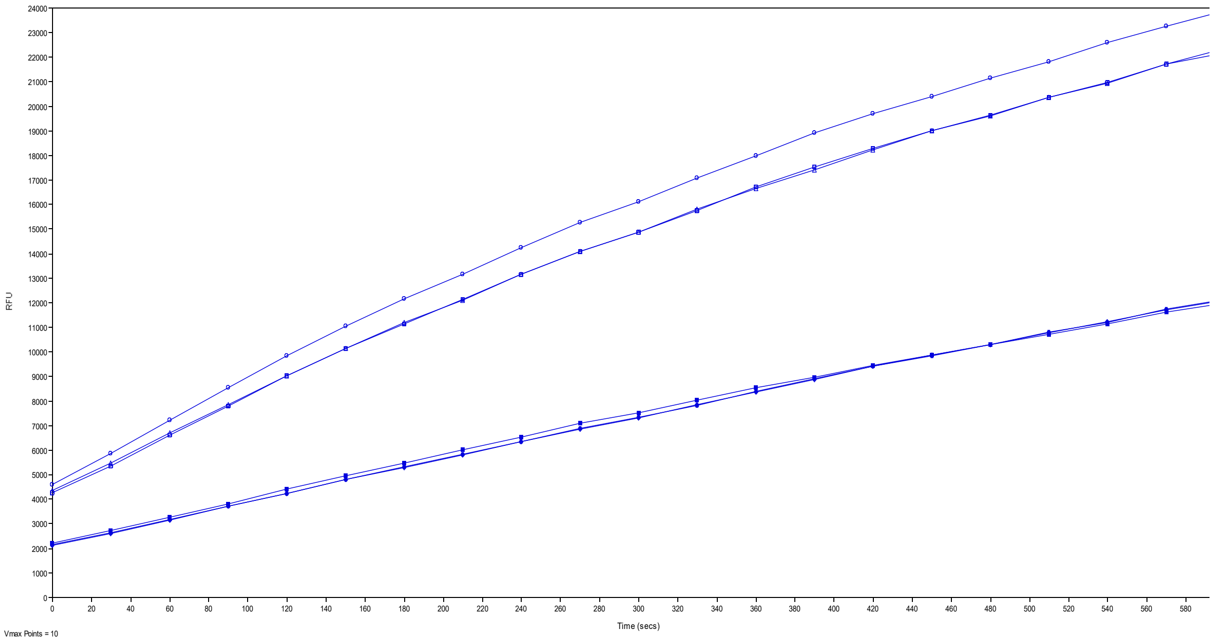

Vmax Points = 10

Well	D1	D2	D3	E7	E8	E9
Vmax Per Second	39.667	36.737	36.339	17.644	17.667	18.112
R^2	0.997	0.998	0.998	1.000	1.000	1.000

Uninhibited β-Gal activity (top curves) and inhibited β-Gal with 50 μM aPEP-2-5 (bottom curves)

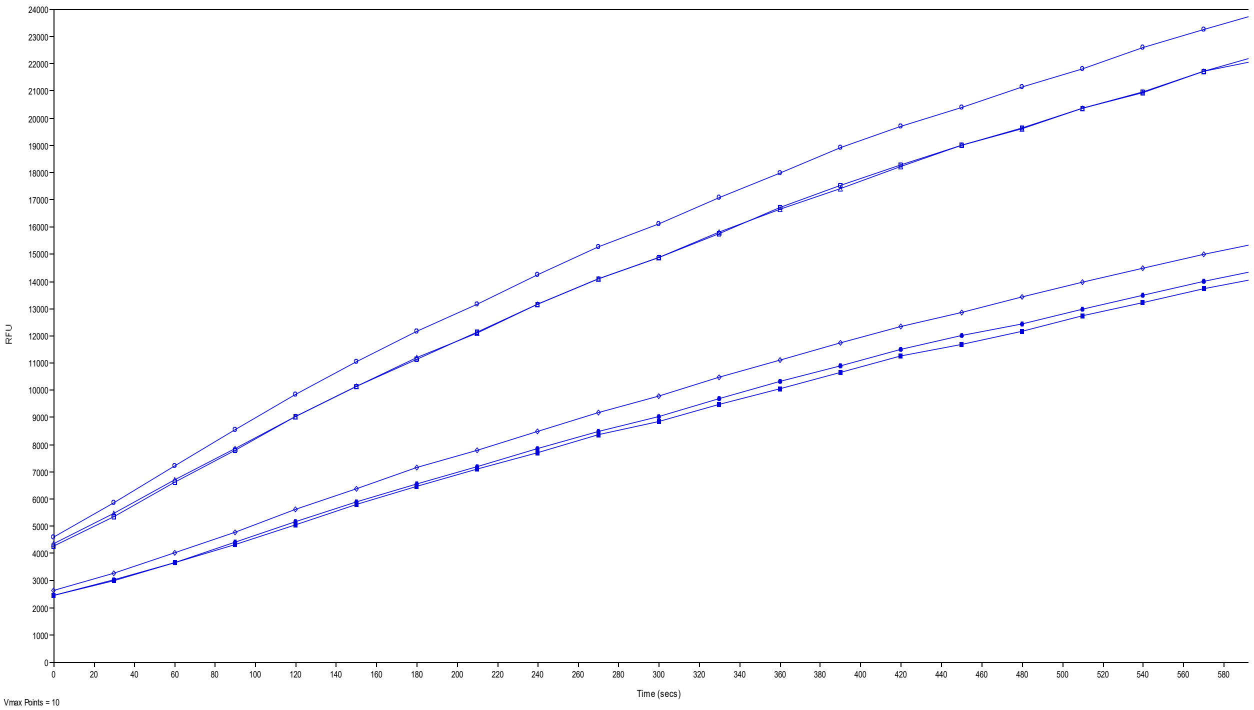

Vmax Points = 10

Well	D1	D2	D3	E10	E11	E12
Vmax Per Second	39.667	36.737	36.339	24.717	22.841	22.268
R^2	0.997	0.998	0.998	0.999	0.999	0.999

Uninhibited β-Gal activity (top curves) and inhibited β-Gal with 50 μM aPEP-2-6 (bottom curves)

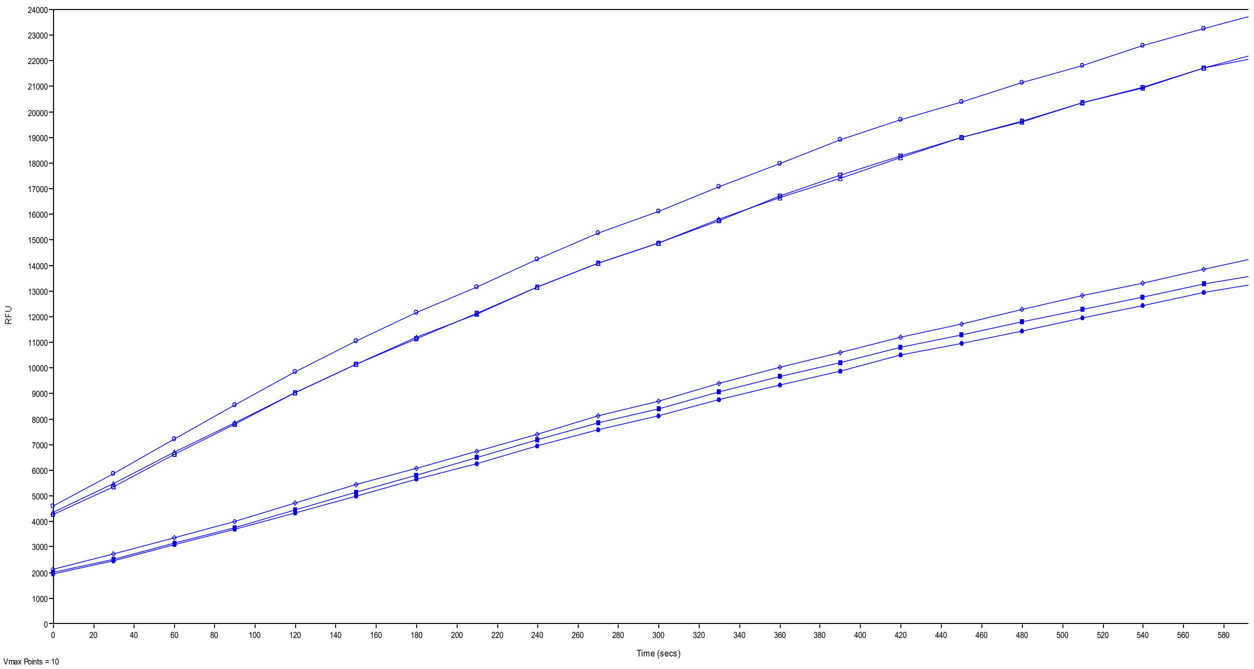

Vmax Points = 10

Well	D1	D2	D3	F4	F5	F6
Vmax Per Second	39.667	36.737	36.339	22.388	21.235	22.218
R^2	0.997	0.998	0.998	1.000	1.000	0.999

Uninhibited β-Gal activity (top curves) and inhibited β-Gal with 50 μM aPEP-2-7 (bottom curves)

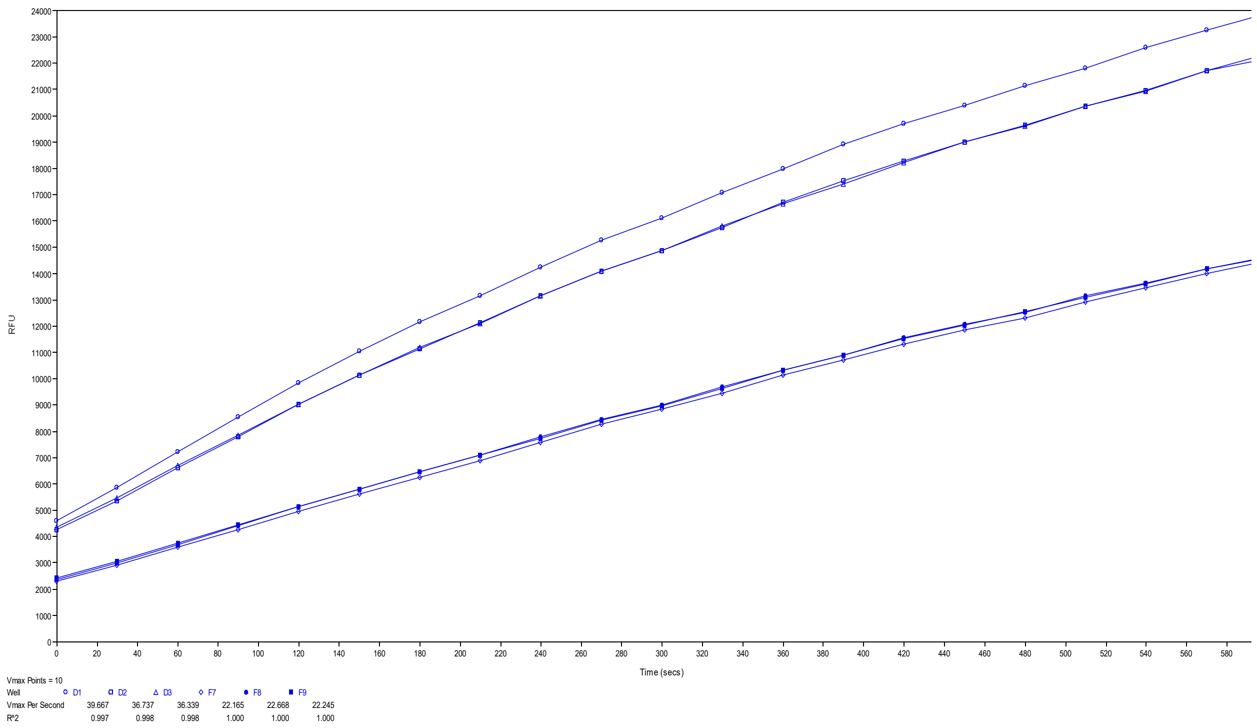

Uninhibited β-Gal activity (top curves) and inhibited β-Gal with 50 μM aPEP-2-8 (bottom curves)

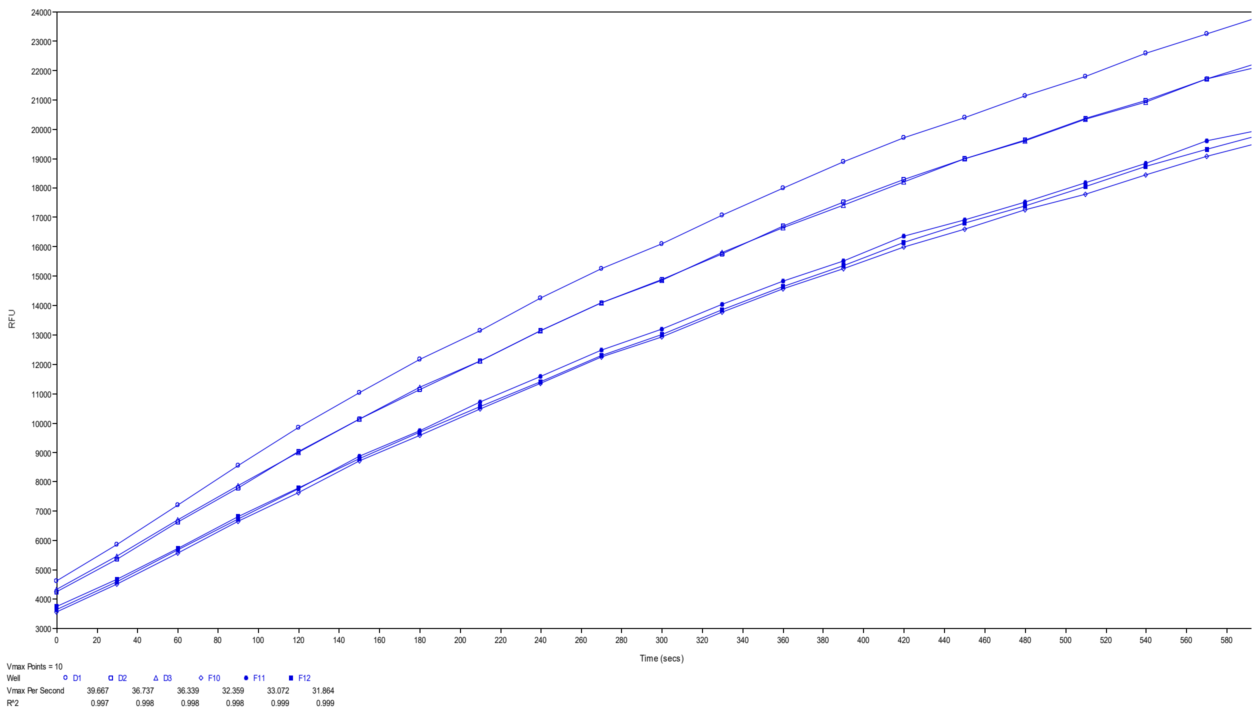

Uninhibited β-Gal activity (top curves) and inhibited β-Gal with 50 μM aPEP-2-9 (bottom curves)

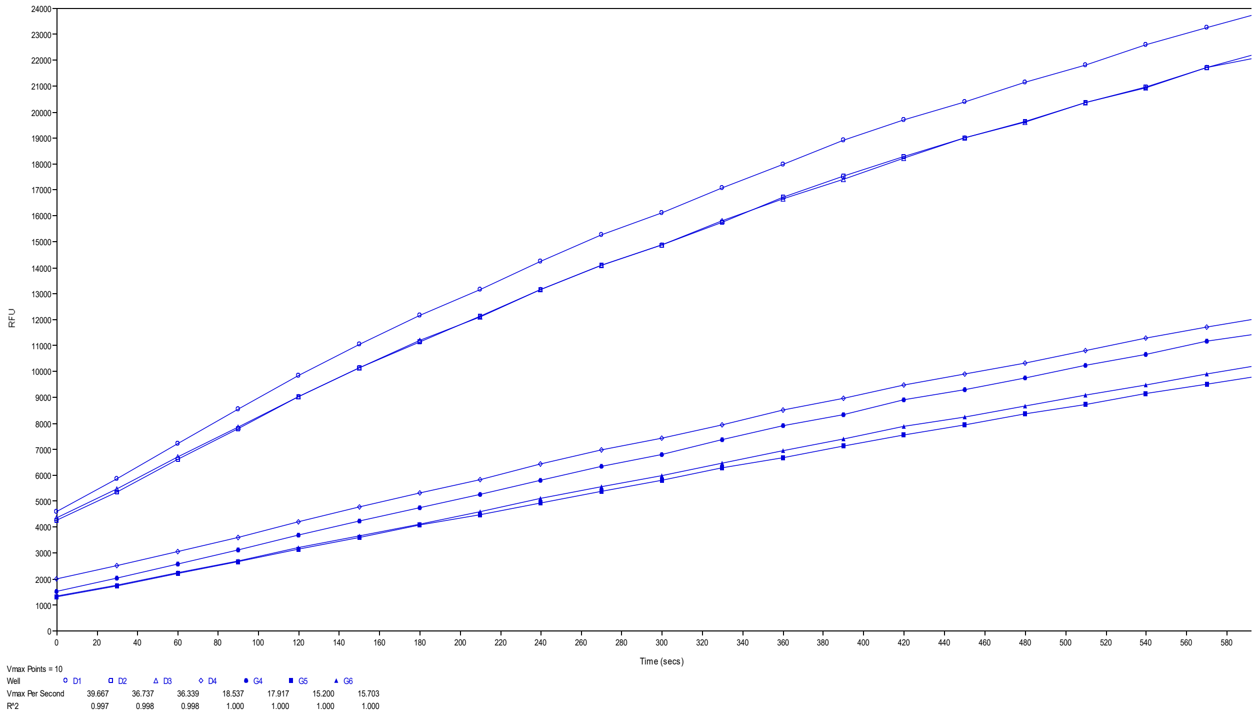

Uninhibited β-Gal activity (top curves) and inhibited β-Gal with 50 μM aPEP-2-10 (bottom curves)

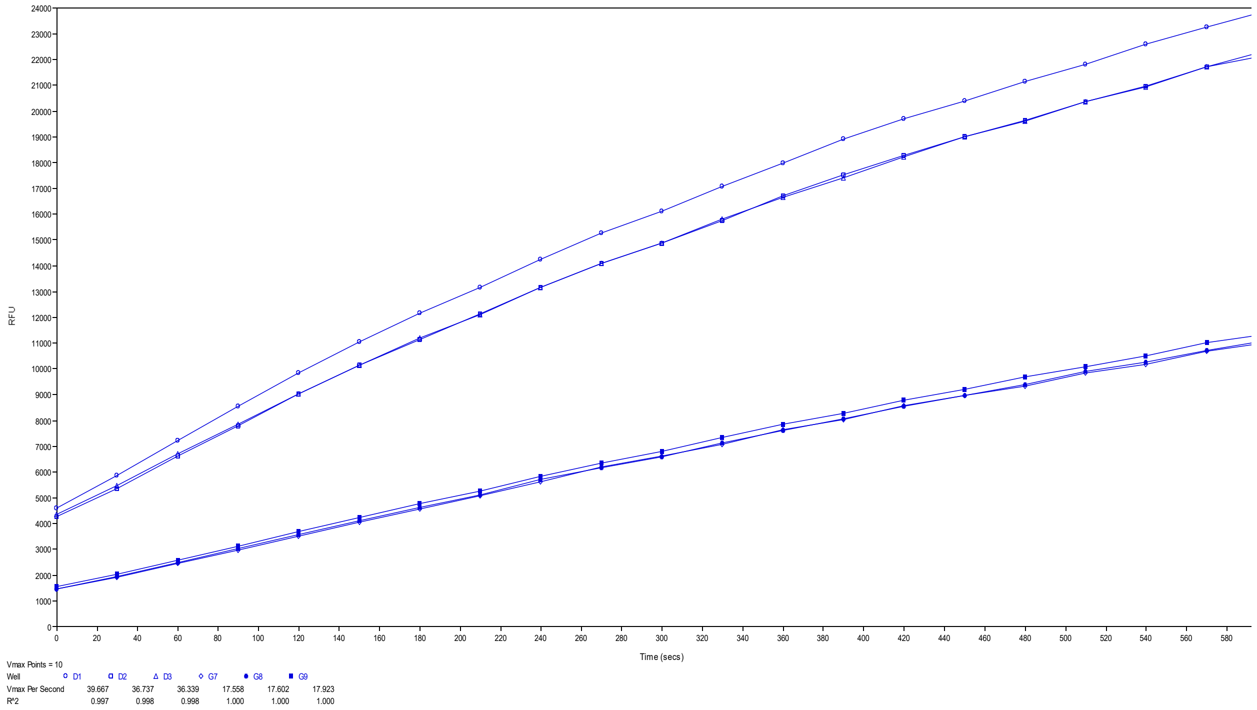

Uninhibited β-Gal activity (top curves) and inhibited β-Gal with 50 μM aPEP-2-11 (bottom curves)

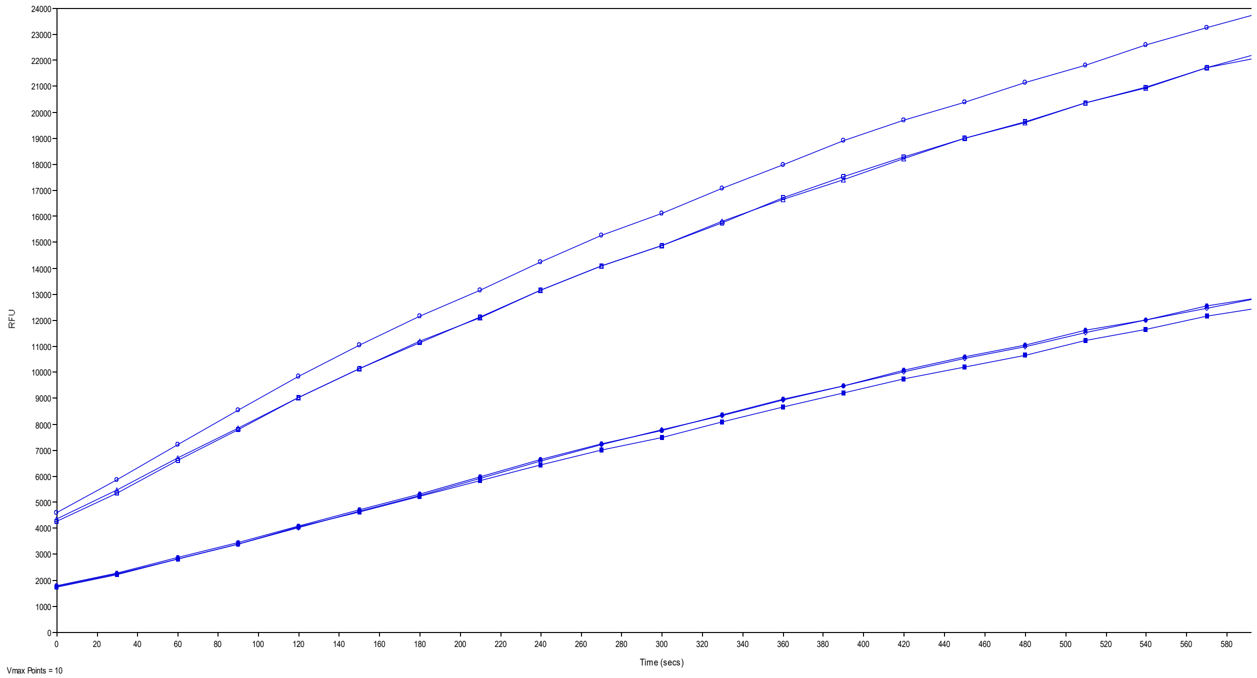

Vmax Points = 10

Well	D1	D2	D3	G10	G11	G12
Vmax Per Second	39.667	36.737	36.339	20.792	20.641	19.825
R^2	0.997	0.998	0.998	1.000	1.000	1.000

Uninhibited β-Gal activity (top curves) and inhibited β-Gal with 50 μM aPEP-2-12 (bottom curves)

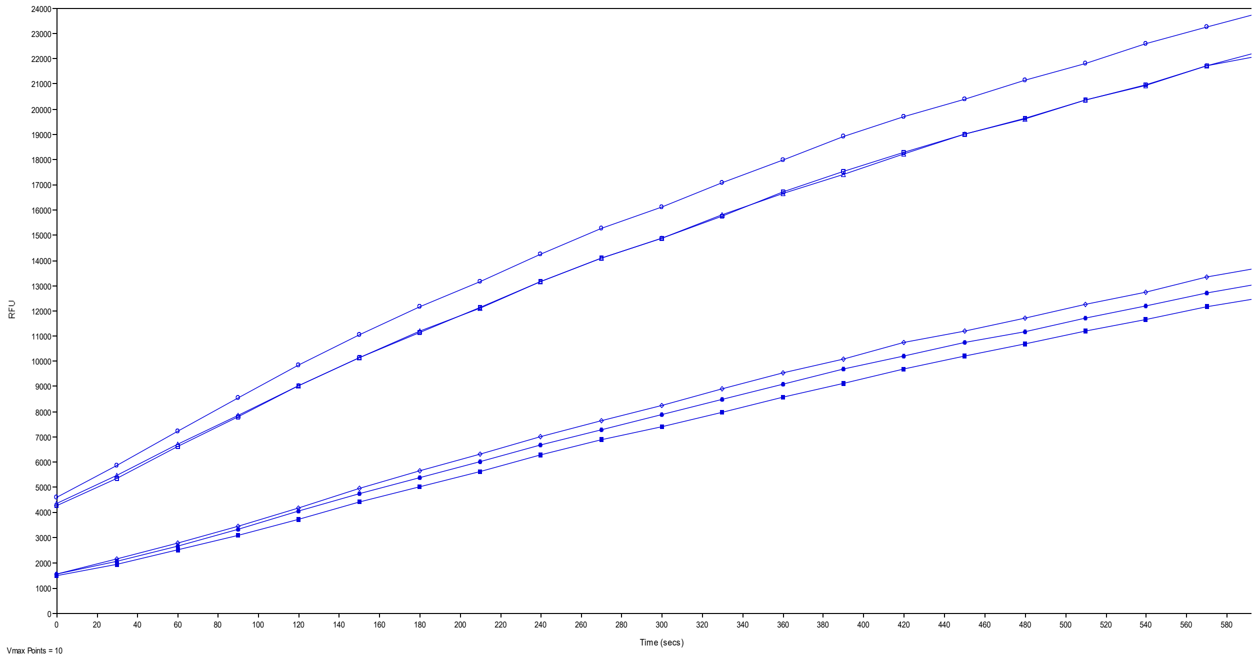

Vmax Points = 10

Well	D1	D2	D3	H4	H5	H6
Vmax Per Second	39.667	36.737	36.339	23.004	21.810	20.646
R^2	0.997	0.998	0.998	0.999	0.999	0.999

Uninhibited β-Gal activity (top curves) and inhibited β-Gal with 50 μM aPEP-2-13 (bottom curves)

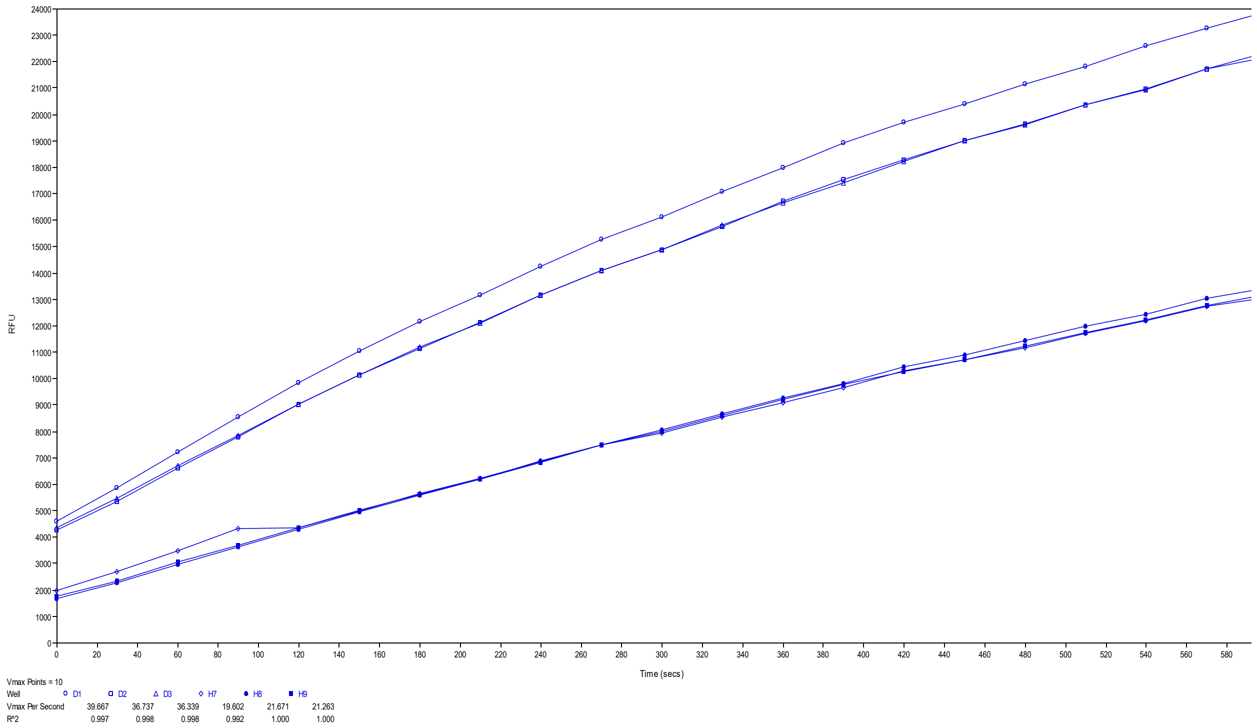

Uninhibited β-Gal activity (top curves) and inhibited β-Gal with 50 μM aPEP-2-14 (bottom curves)

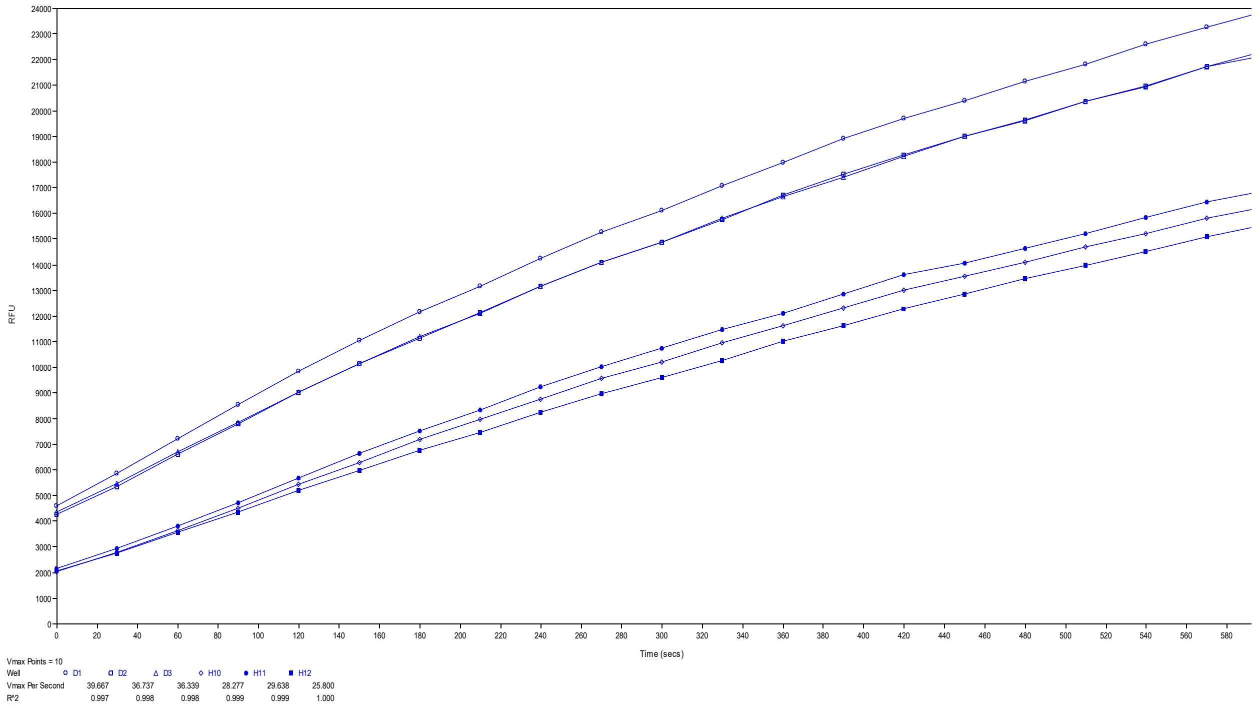

Uninhibited β-Gal activity (top curves) and inhibited β-Gal with 50 μM aPEP-2-15 (bottom curves)

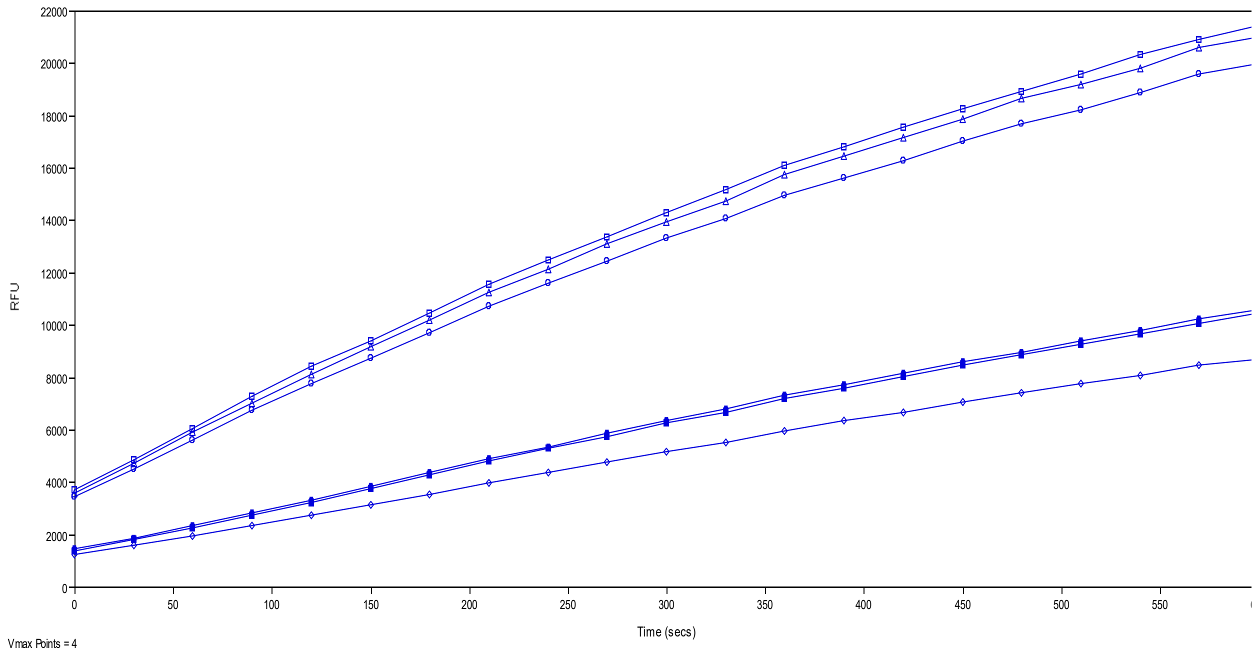

Vmax Points = 4

Well	A1	A2	A3	A4	A5	A6
Vmax Per Second	36.645	39.936	38.183	13.747	17.316	17.541
R^2	0.999	1.000	1.000	1.000	1.000	1.000

Uninhibited β-Gal activity (top curves) and inhibited β-Gal with 50 μM aPEP-2-16 (bottom curves)
