# Supplementary material for: Computational and experimental analysis of short peptide motifs for enzyme inhibition
Source: PLoS One. 2017 Aug 15;12(8):e0182847. doi: 10.1371/journal.pone.0182847 (PMC5557489; doi:10.1371/journal.pone.0182847)
Supplement: S6 Fig — (PDF) [file pone.0182847.s007.pdf]

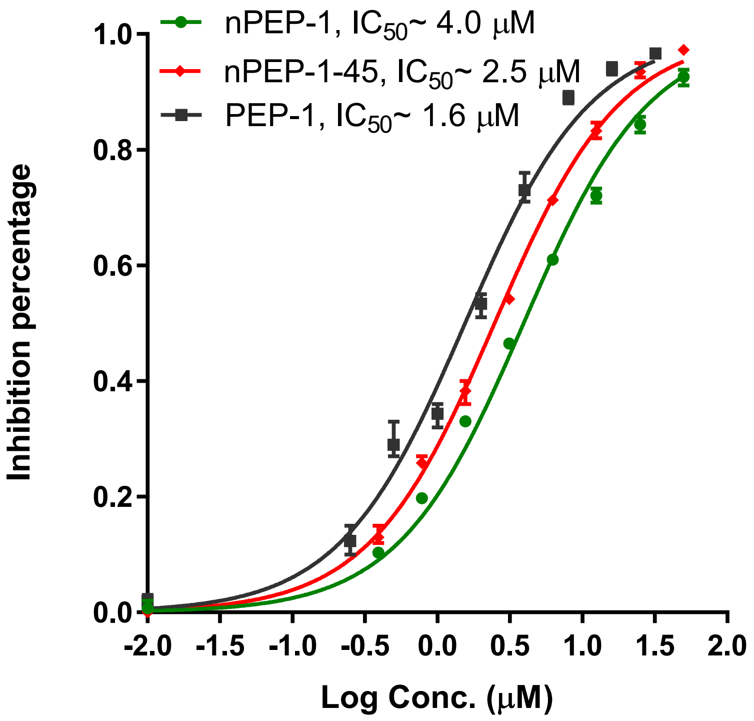

S6 Fig. Curve fit determining the IC50 values of of nPEP-1 "FKRYKRWGSC" (green), nPEP-1-45 "FKRYRRWGSC" (red) and PEP-1 (black) for inhibiting β-Gal. GraphPad Prism 5 was used for enzyme kinetics fitting to the equation: Y=Bottom + (Top-Bottom)/(1+10^(X-LogIC50)). Here, Bottom is constrained to 1 which represents the maximal inhibition of 100% and Top is constrained to 0 which represents the minimal inhibition of 0%. All tests included three replicates. Error bar: range of data.
