# Supplementary material for: Computational and experimental analysis of short peptide motifs for enzyme inhibition
Source: PLoS One. 2017 Aug 15;12(8):e0182847. doi: 10.1371/journal.pone.0182847 (PMC5557489; doi:10.1371/journal.pone.0182847)
Supplement: S7 Fig — (PDF) [file pone.0182847.s008.pdf]

S7 Fig. Point-variant screening of nPEP-1 peptide "FKRYKRWGSG" at each of the 7 N-terminal positions with a substitution set of S, Y, E, L, W, Q, and R. 50 µM peptide was incubated with 500 µg/L β-Gal and 100 µM RBG in 10 mM potassium phosphate buffer with 0.1 mM $MgCl_2$ (pH 7.4), 25 °C.

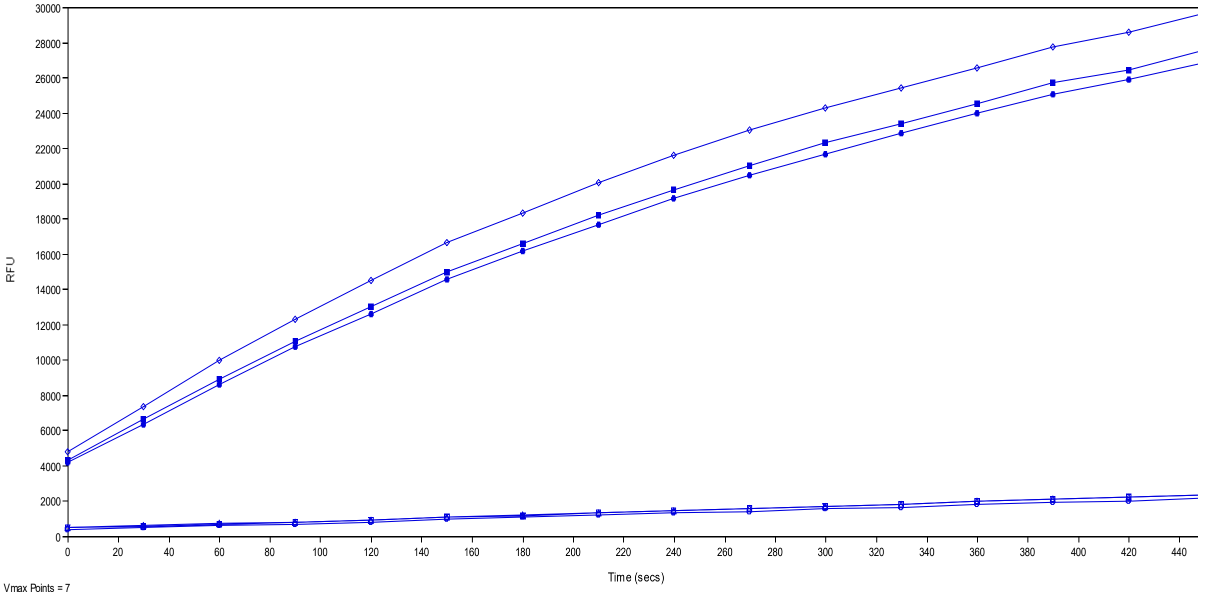

Vmax Points = 7

Well	A4	A5	A6	G1	G2	G3
Vmax Per Second	4.055	4.386	4.343	75.946	67.123	68.710
R^2	0.999	0.999	0.999	0.996	0.997	0.997

Uninhibited β-Gal activity (top curves) and inhibited β-Gal with 50 µM nPEP-1-1 (bottom curves).

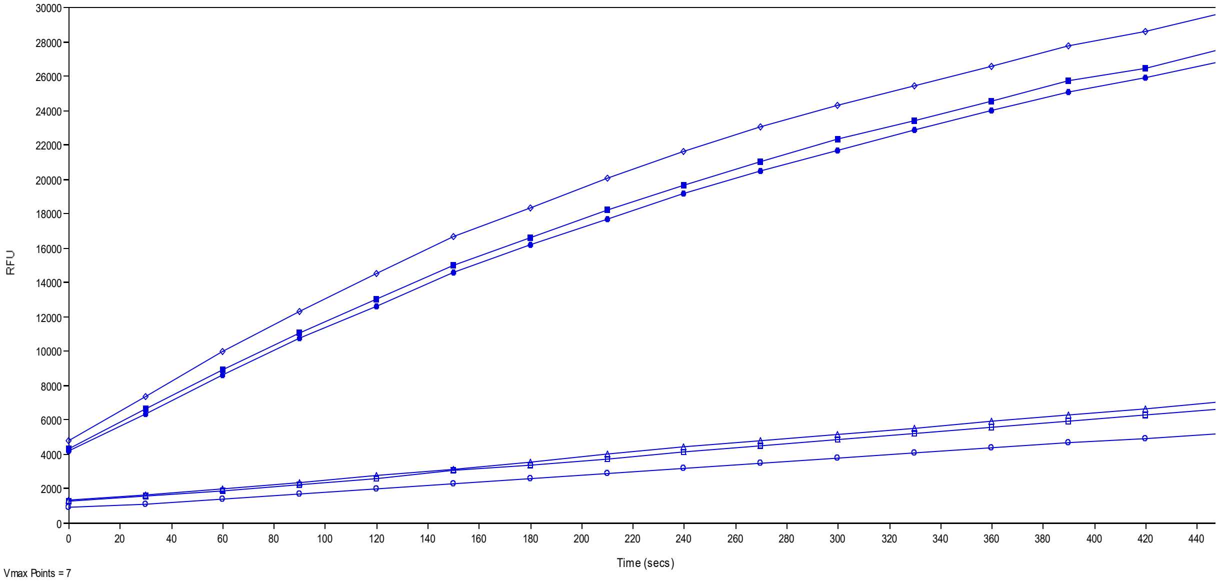

Vmax Points = 7

Well	B4	B5	B6	G1	G2	G3
Vmax Per Second	10.201	12.580	13.752	75.946	67.123	68.710
R^2	1.000	0.999	0.999	0.996	0.997	0.997

Uninhibited β-Gal activity (top curves) and inhibited β-Gal with 50 µM nPEP-1-2 (bottom).

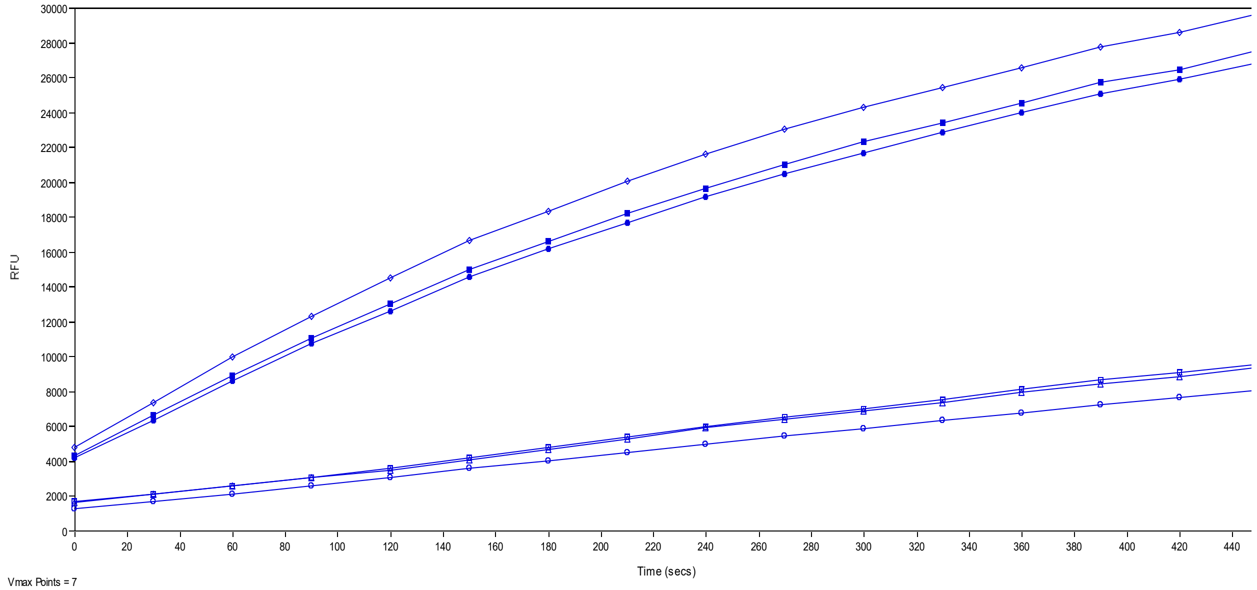

Vmax Points = 7

Well	C4	C5	C6	G1	G2	G3
Vmax Per Second	16.086	19.390	19.125	75.946	67.123	68.710
R^2	1.000	0.999	0.999	0.996	0.997	0.997

Uninhibited β-Gal activity (top curves) and inhibited β-Gal with 50 μM nPEP-1-3 (bottom curves).

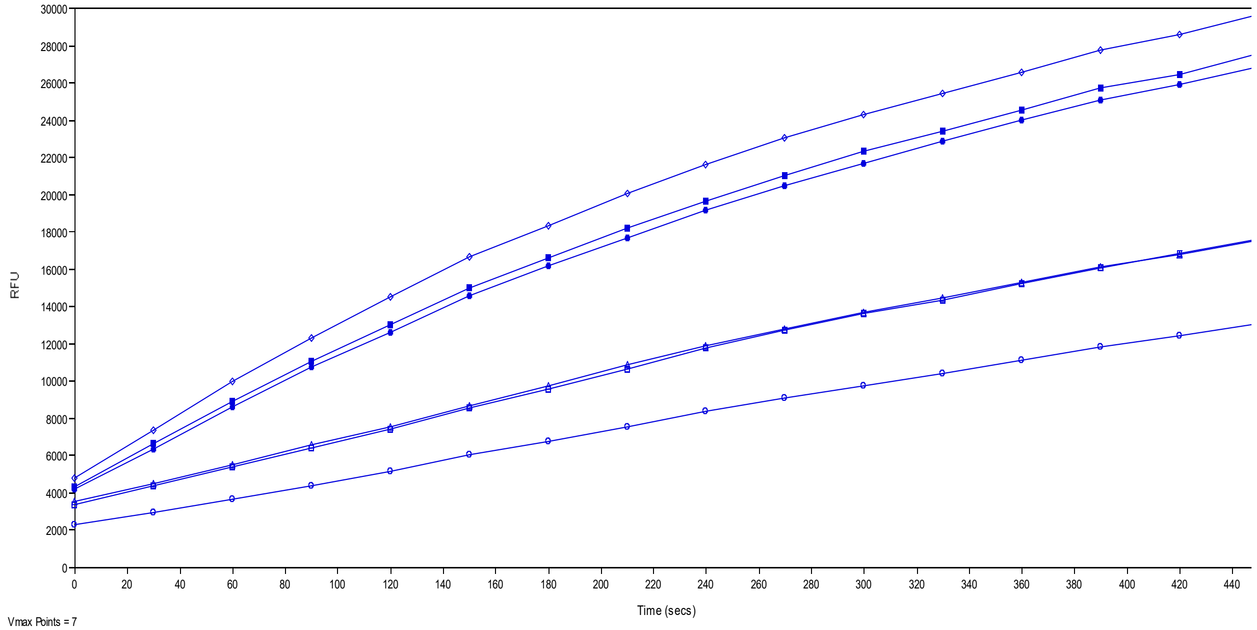

Vmax Points = 7

Well	D4	D5	D6	G1	G2	G3
Vmax Per Second	26.307	35.494	35.515	75.946	67.123	68.710
R^2	1.000	1.000	1.000	0.996	0.997	0.997

Uninhibited β-Gal activity (top curves) and inhibited β-Gal with 50 μM nPEP-1-4 (bottom curves).

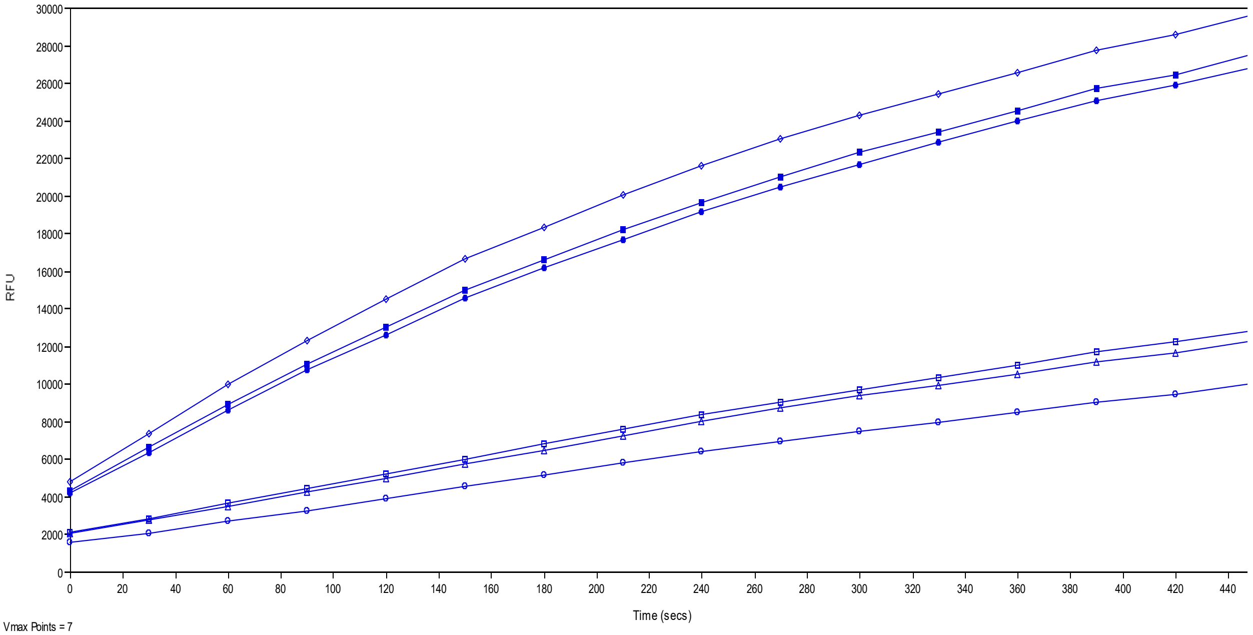

Vmax Points = 7

Well	E4	E5	E6	G1	G2	G3
Vmax Per Second	20.794	26.466	24.928	75.946	67.123	68.710
R^2	1.000	1.000	1.000	0.996	0.997	0.997

Uninhibited β-Gal activity (top curves) and inhibited β-Gal with 50 μM nPEP-1-5 (bottom curves).

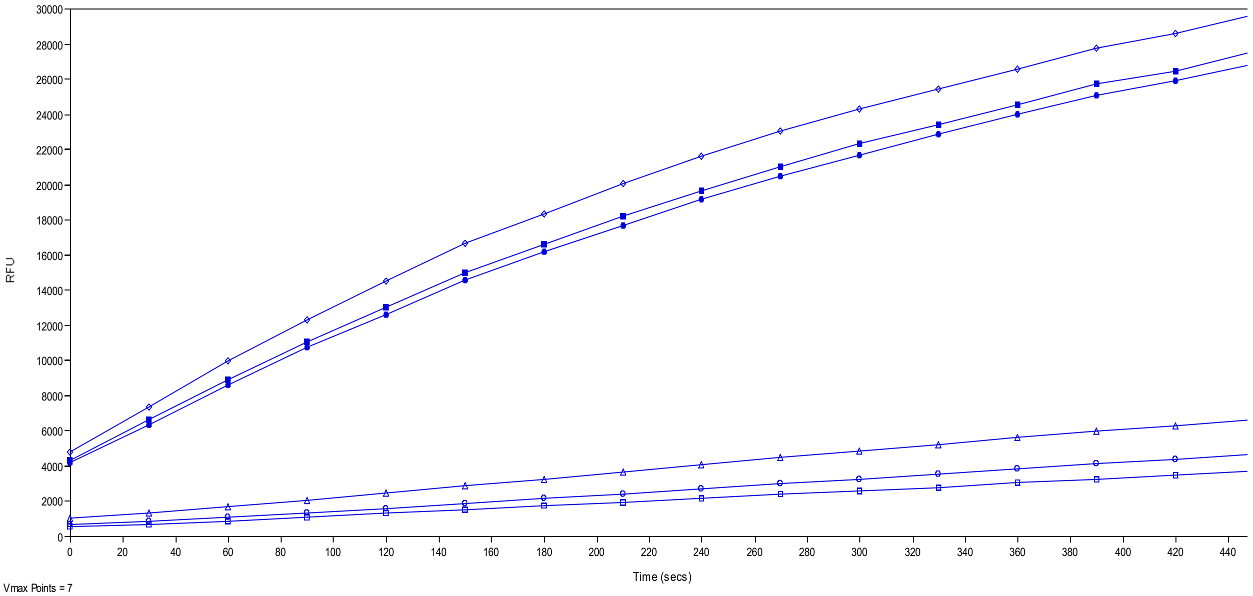

Vmax Points = 7

Well	F4	F5	F6	G1	G2	G3
Vmax Per Second	9.447	7.324	13.389	75.946	67.123	68.710
R^2	1.000	1.000	1.000	0.996	0.997	0.997

Uninhibited β-Gal activity (top curves) and inhibited β-Gal with 50 μM nPEP-1-6 (bottom).

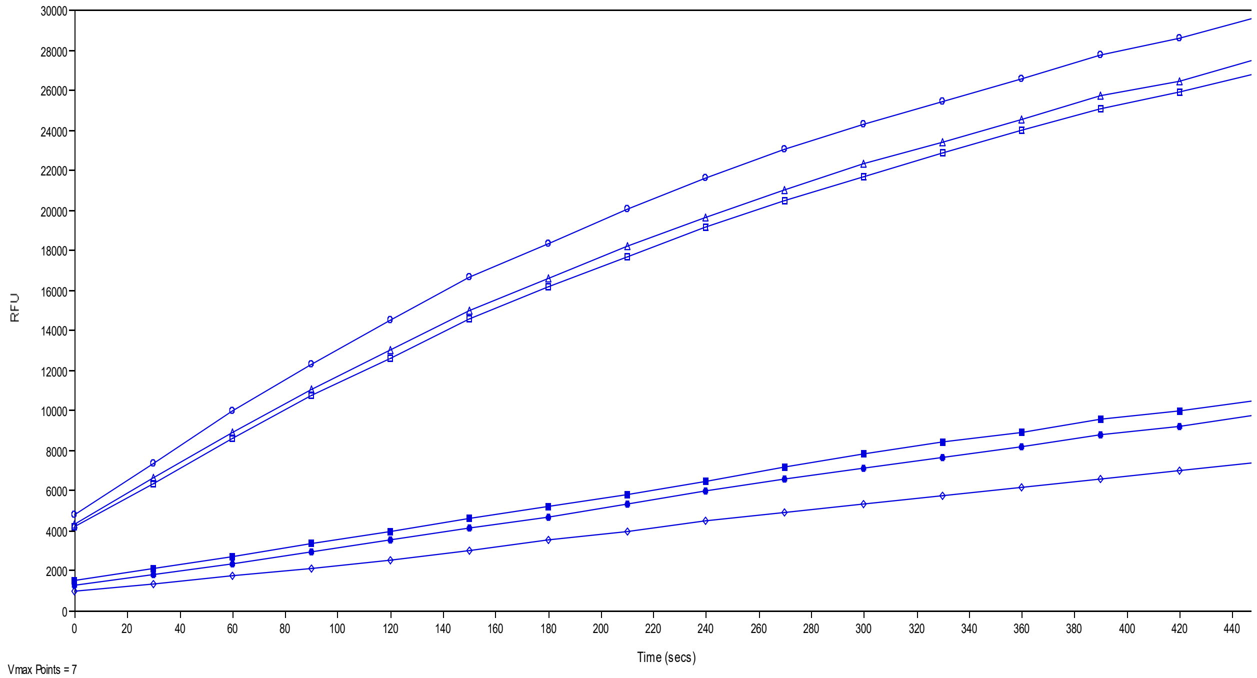

Vmax Points = 7

Well	G1	G2	G3	G4	G5	G6
Vmax Per Second	75.946	67.123	68.710	15.677	20.180	21.522
R^2	0.996	0.997	0.997	1.000	1.000	0.999

Uninhibited β-Gal activity (top curves) and inhibited β-Gal with 50 μM nPEP-1-7 (bottom curves).

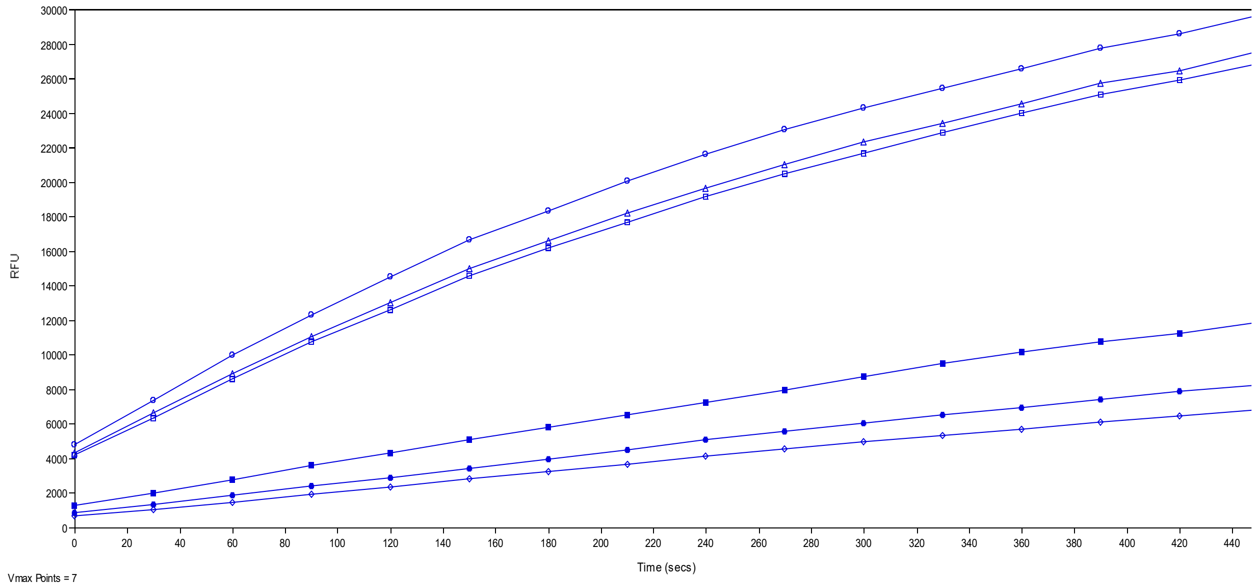

Vmax Points = 7

Well	G1	G2	G3	H4	H5	H6
Vmax Per Second	75.946	67.123	68.710	14.891	17.865	25.414
R^2	0.996	0.997	0.997	1.000	1.000	1.000

Uninhibited β-Gal activity (top curves) and inhibited β-Gal with 50 μM nPEP-1-8 (bottom).

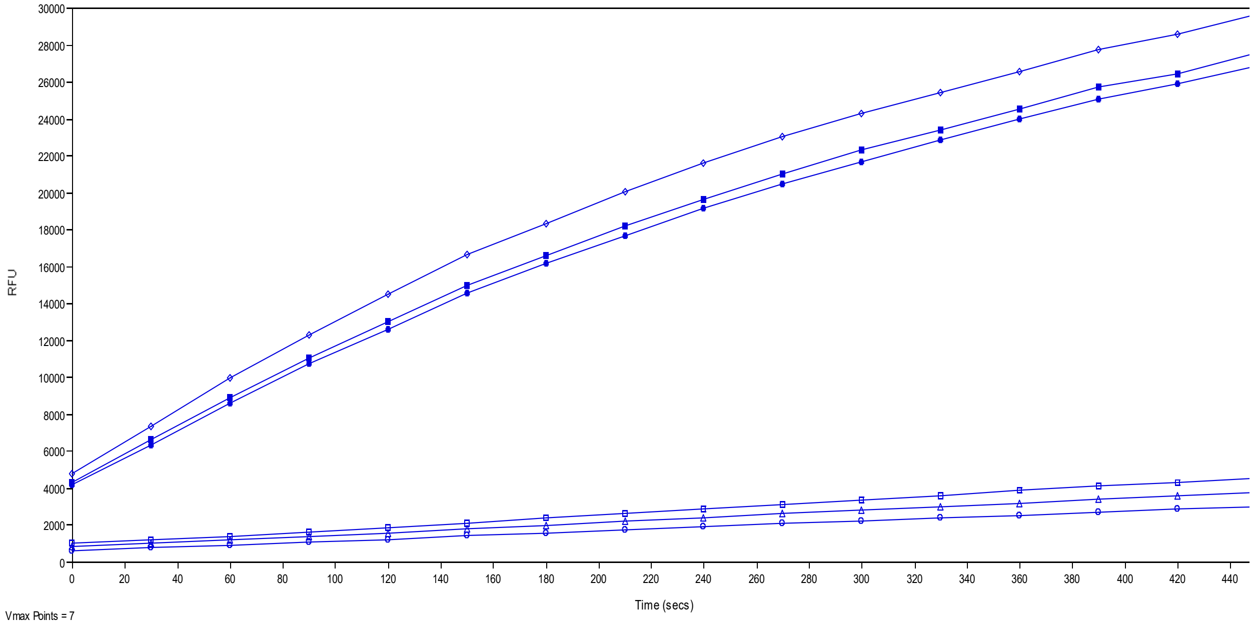

Vmax Points = 7

Well	A7	A8	A9	G1	G2	G3
Vmax Per Second	5.617	8.595	7.082	75.946	67.123	68.710
R^2	0.999	1.000	0.999	0.996	0.997	0.997

Uninhibited β-Gal activity (top curves) and inhibited β-Gal with 50 μM nPEP-1-9 (bottom curves).

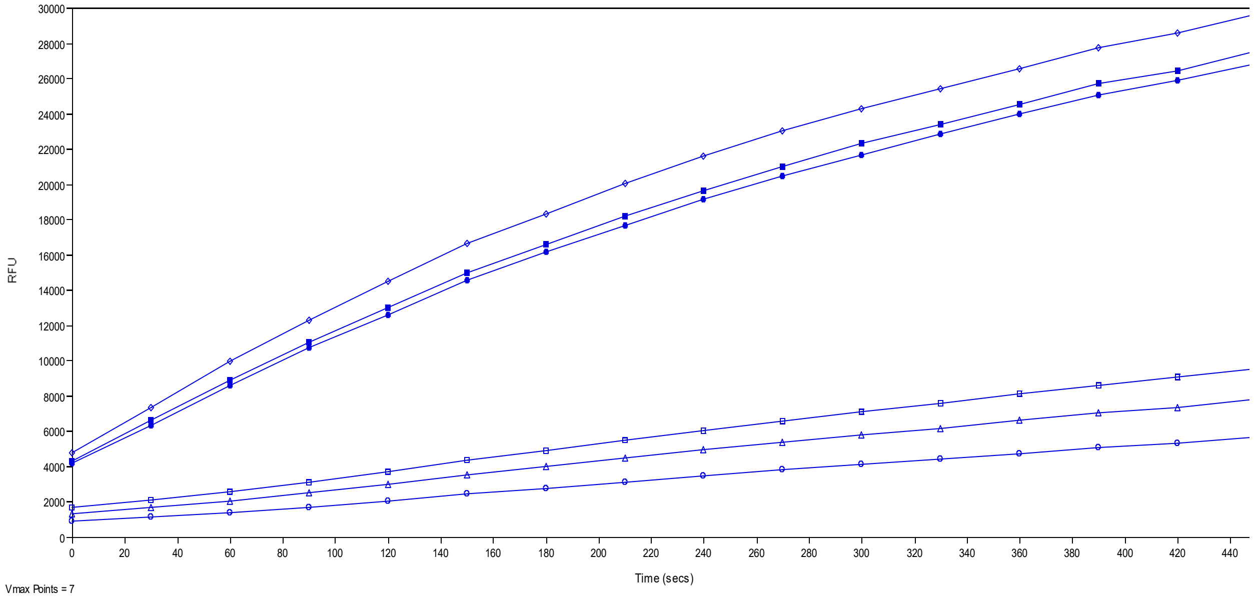

Vmax Points = 7

Well	B7	B8	B9	G1	G2	G3
Vmax Per Second	11.719	19.460	16.100	75.946	67.123	68.710
R^2	1.000	1.000	1.000	0.996	0.997	0.997

Uninhibited β-Gal activity (top curves) and inhibited β-Gal with 50 μM nPEP-1-10 (bottom curves).

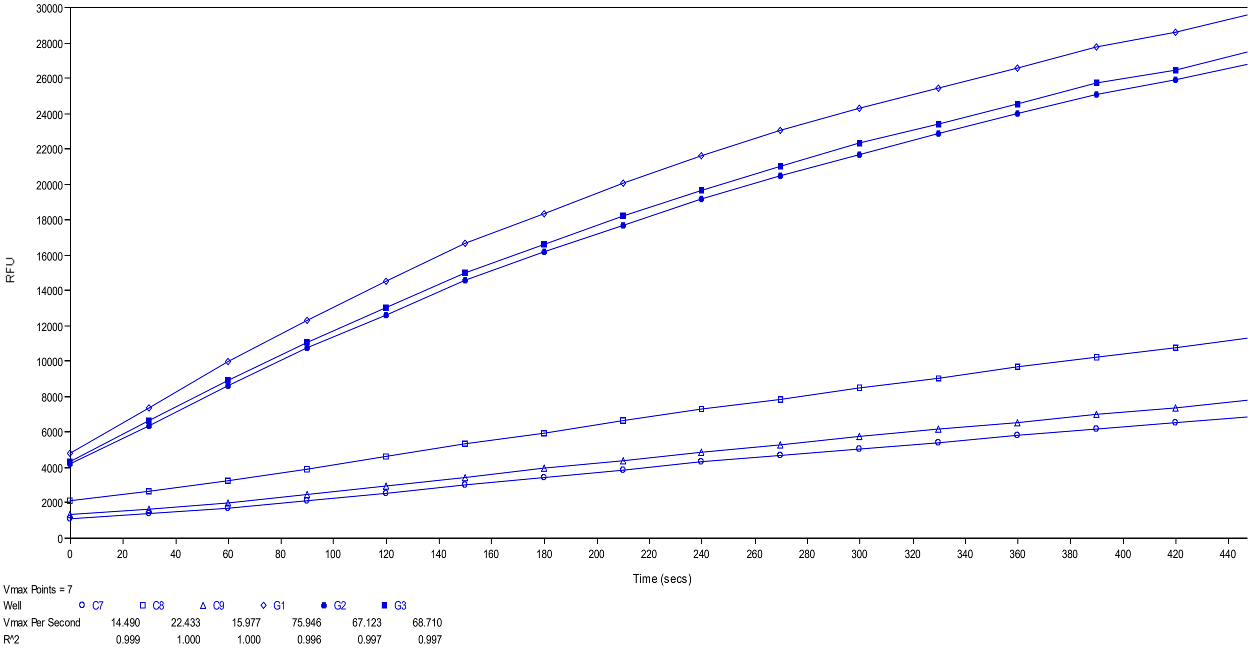

Uninhibited β-Gal activity (top curves) and inhibited β-Gal with 50 μM nPEP-1-11 (bottom curves).

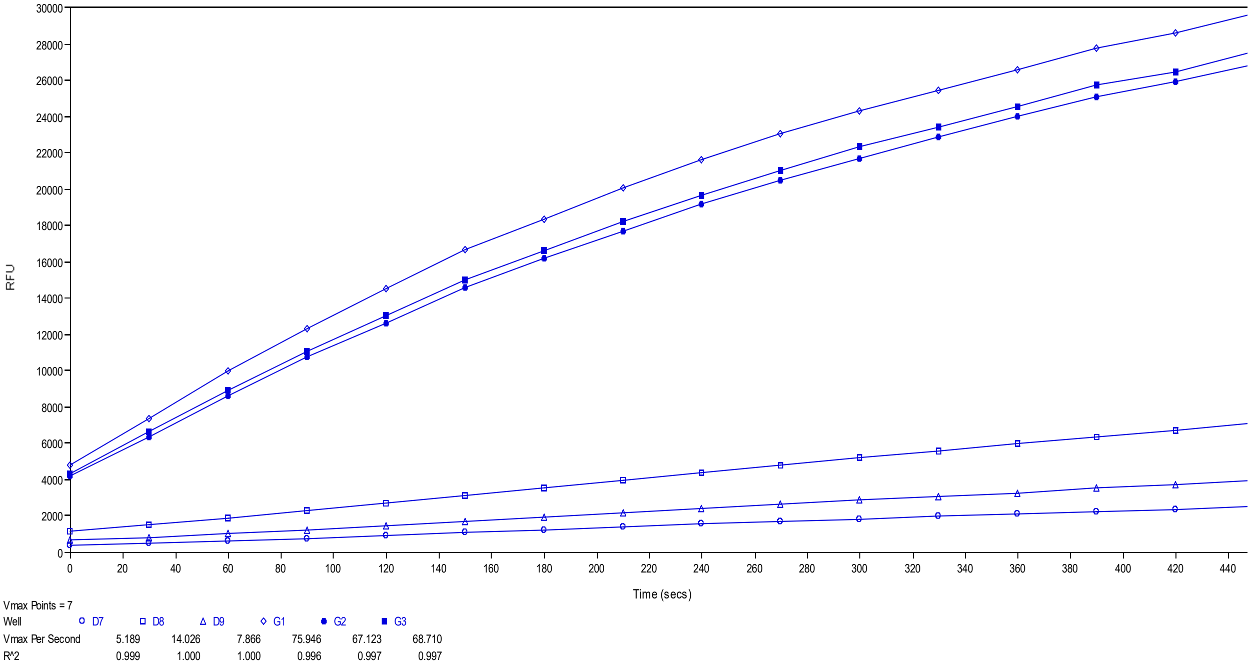

Uninhibited β-Gal activity (top curves) and inhibited β-Gal with 50 μM nPEP-1-12 (bottom).

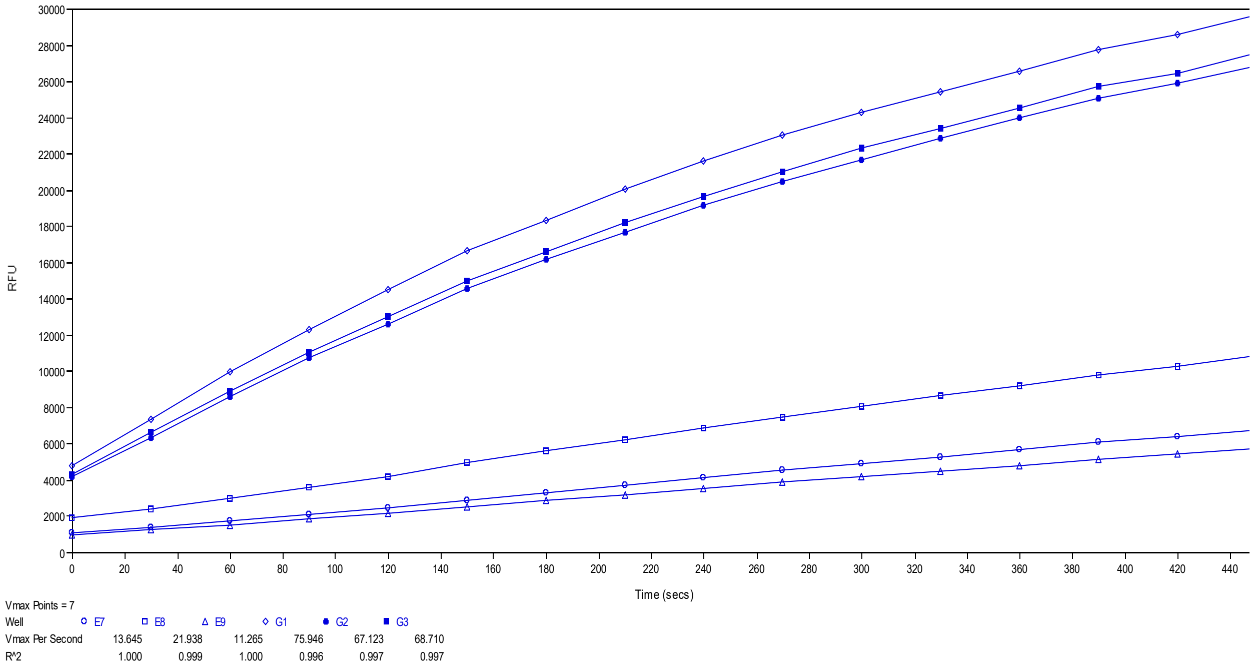

Uninhibited β-Gal activity (top curves) and inhibited β-Gal with 50 μM nPEP-1-13 (bottom curves).

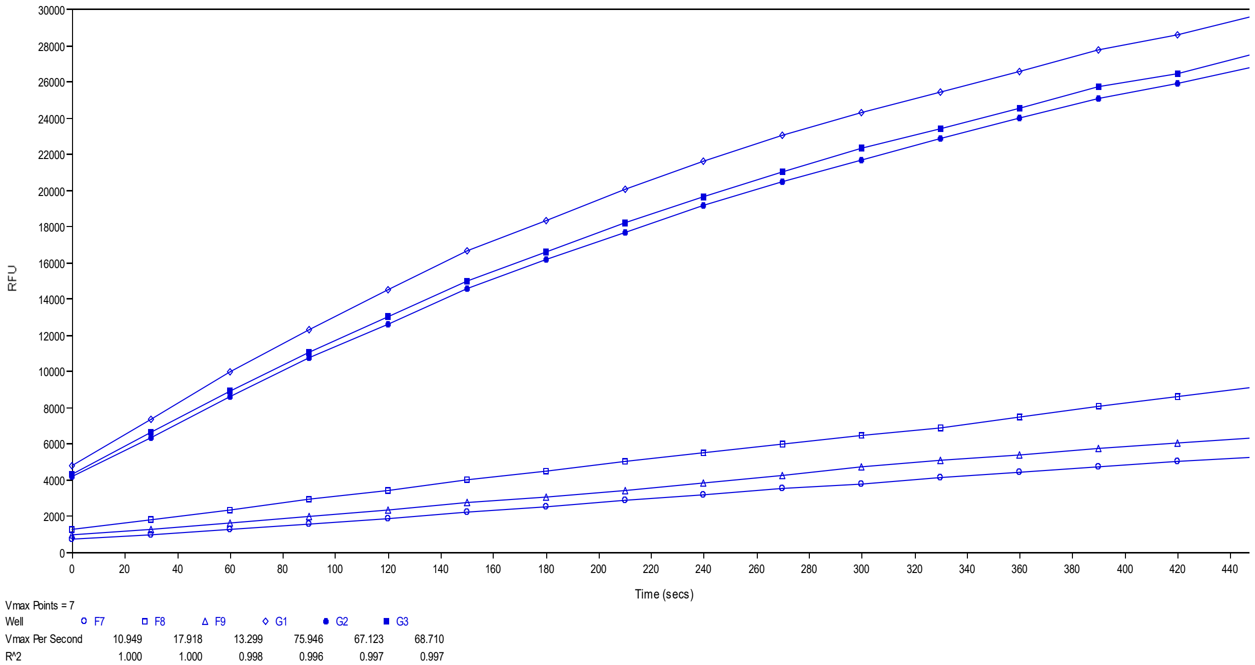

Uninhibited β-Gal activity (top curves) and inhibited β-Gal with 50 μM nPEP-1-14 (bottom curves).

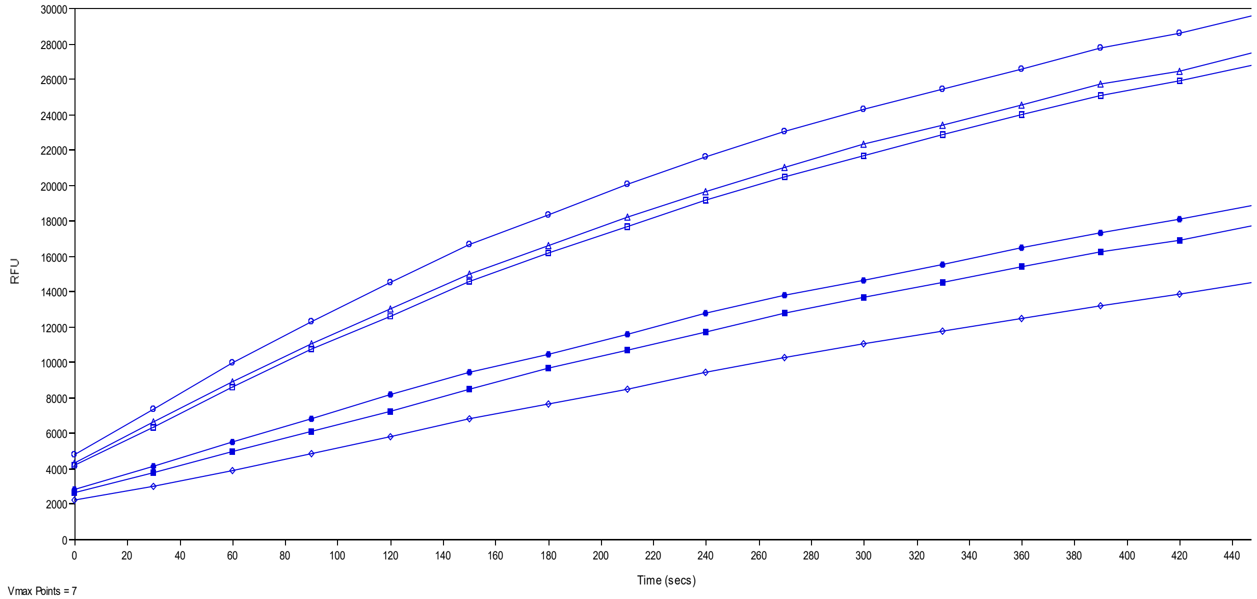

Vmax Points = 7

Well	G1	G2	G3	G7	G8	G9
Vmax Per Second	75.946	67.123	68.710	31.032	43.050	39.087
R^2	0.996	0.997	0.997	0.999	0.999	1.000

Uninhibited β-Gal activity (top curves) and inhibited β-Gal with 50 μM nPEP-1-15 (bottom curves).

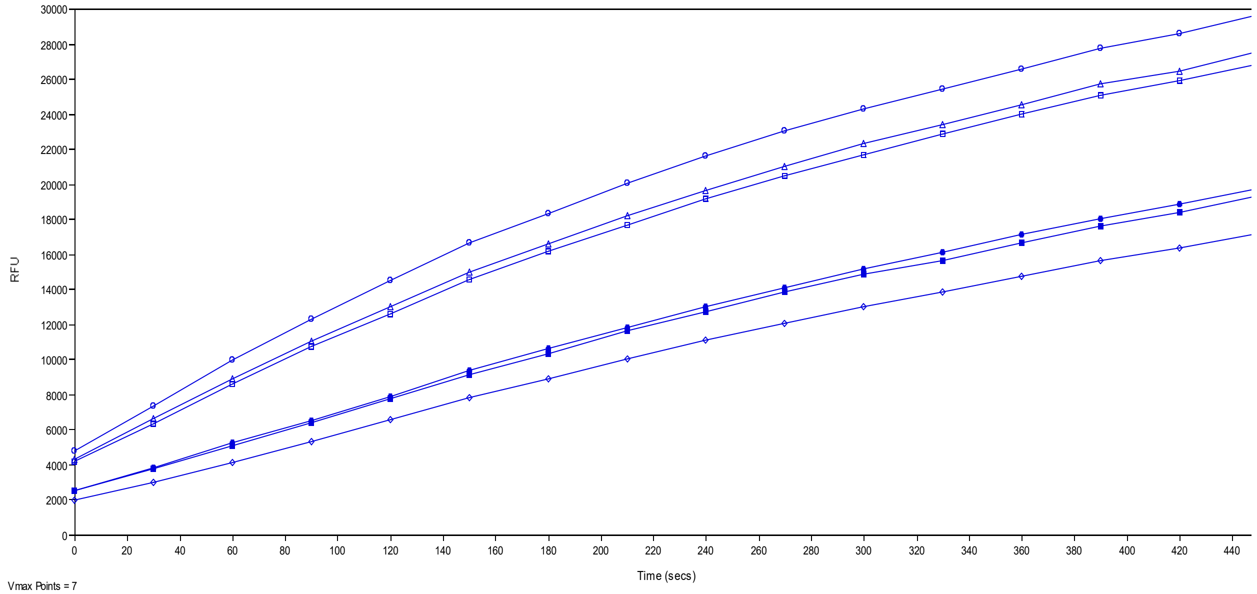

Vmax Points = 7

Well	G1	G2	G3	H7	H8	H9
Vmax Per Second	75.946	67.123	68.710	39.459	45.293	43.991
R^2	0.996	0.997	0.997	1.000	1.000	1.000

Uninhibited β-Gal activity (top curves) and inhibited β-Gal with 50 μM nPEP-1-16 (bottom curves).

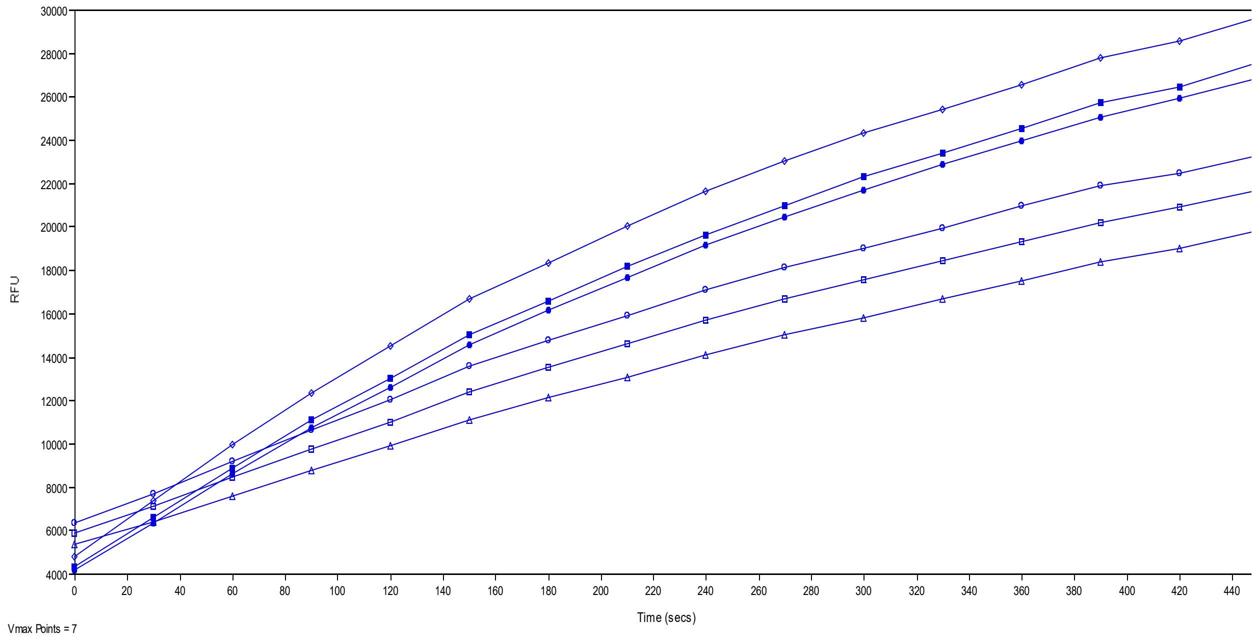

Vmax Points = 7

Well	A10	A11	A12	G1	G2	G3
Vmax Per Second	47.432	42.788	38.115	75.946	67.123	68.710
R^2	0.999	1.000	1.000	0.996	0.997	0.997

Uninhibited β-Gal activity (top curves) and inhibited β-Gal with 50 μM nPEP-1-17 (bottom curves).

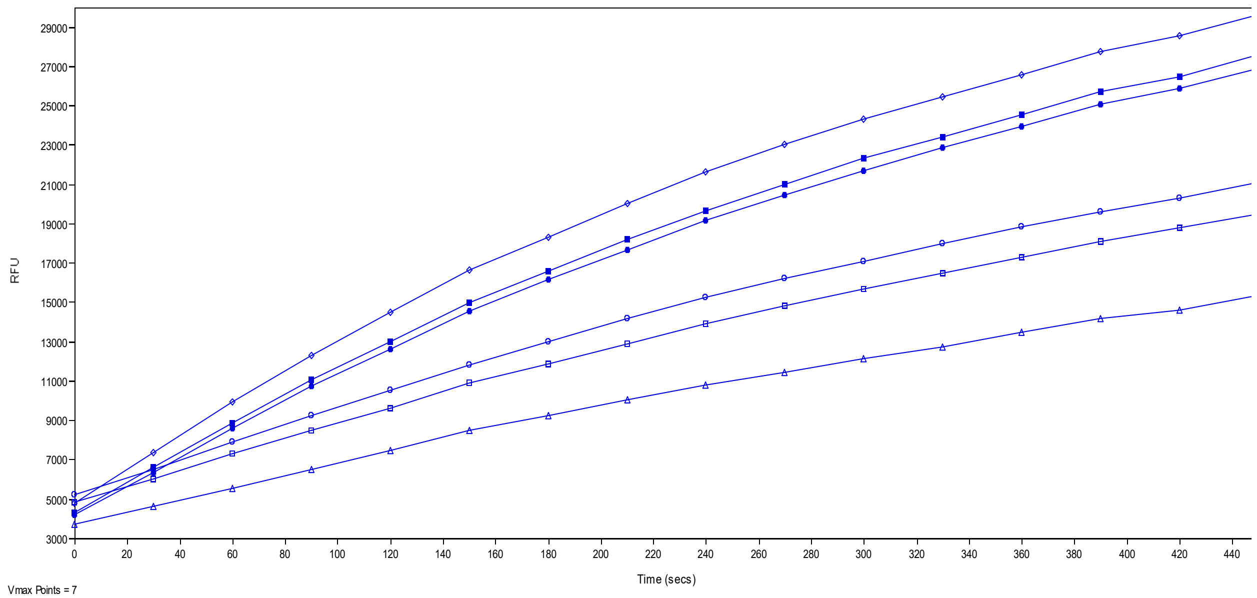

Vmax Points = 7

Well	B10	B11	B12	G1	G2	G3
Vmax Per Second	43.652	39.327	31.186	75.946	67.123	68.710
R^2	1.000	0.999	0.999	0.996	0.997	0.997

Uninhibited β-Gal activity (top curves) and inhibited β-Gal with 50 μM nPEP-1-18 (bottom curves).

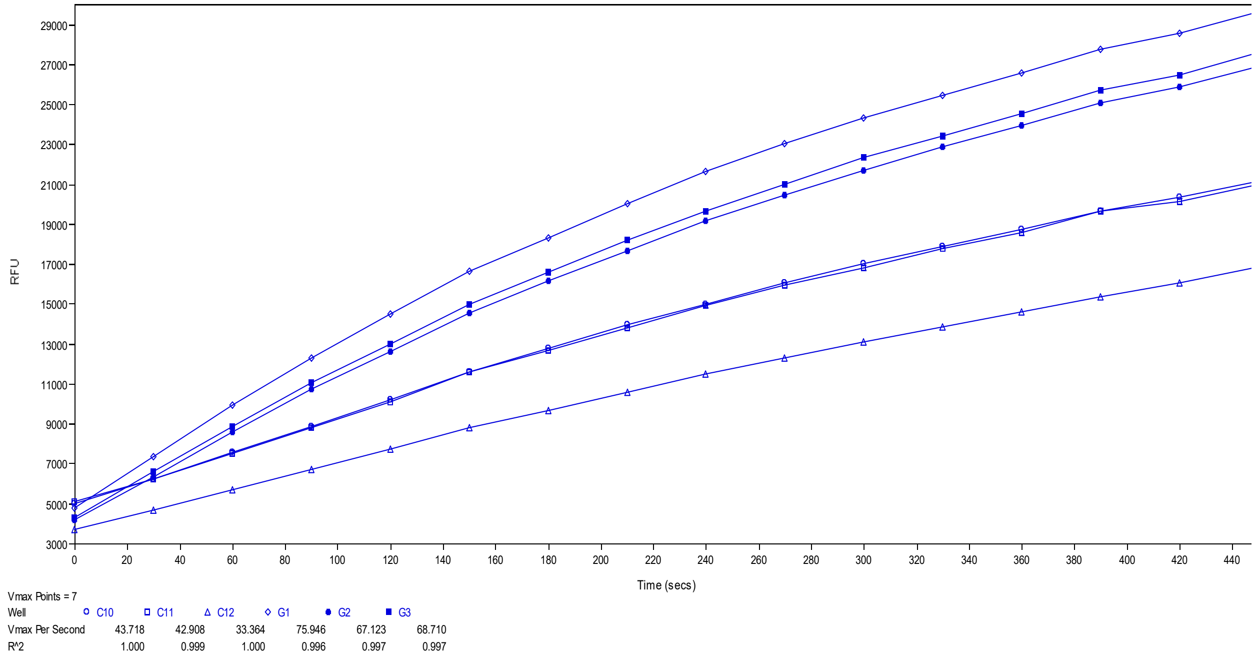

Uninhibited β-Gal activity (top curves) and inhibited β-Gal with 50 μM nPEP-1-19 (bottom curves).

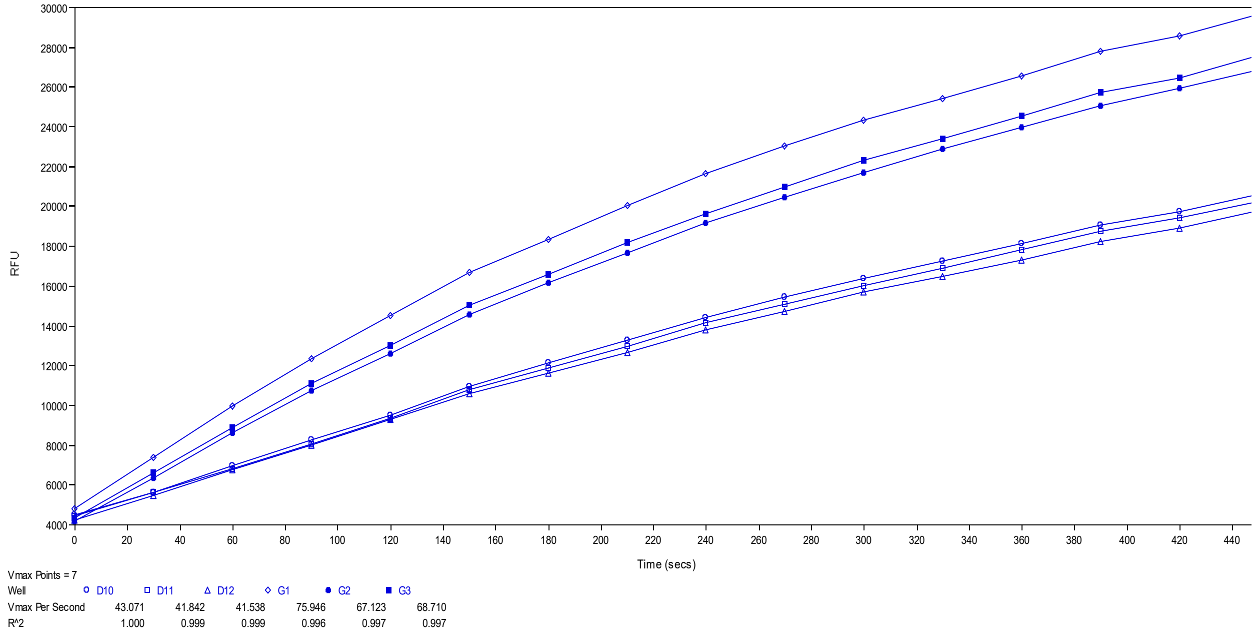

Uninhibited β-Gal activity (top curves) and inhibited β-Gal with 50 μM nPEP-1-20 (bottom curves).

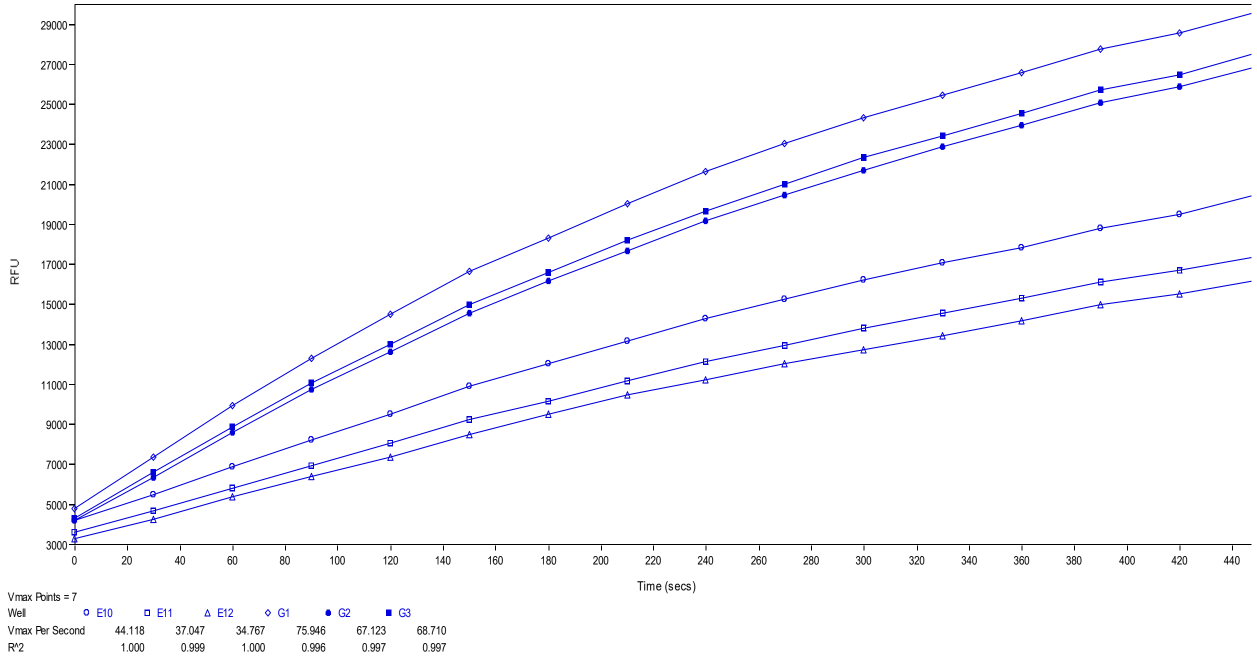

Uninhibited β-Gal activity (top curves) and inhibited β-Gal with 50 μM nPEP-1-21 (bottom curves).

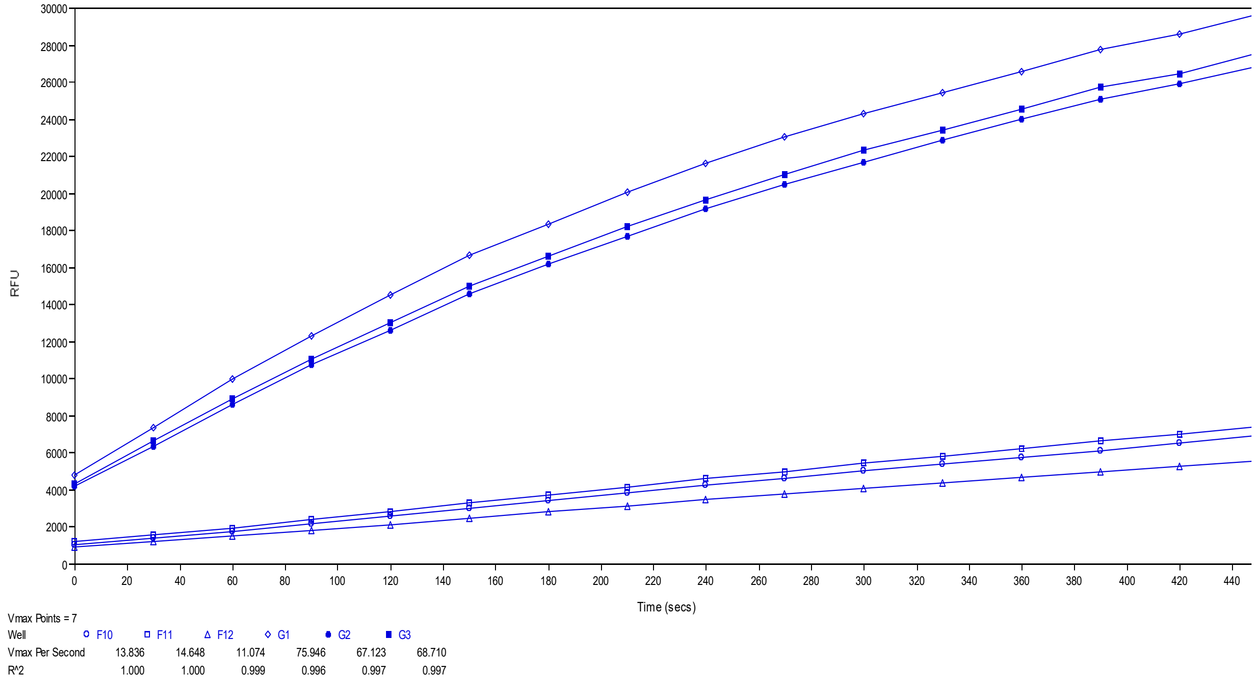

Uninhibited β-Gal activity (top curves) and inhibited β-Gal with 50 μM nPEP-1-22 (bottom curves).

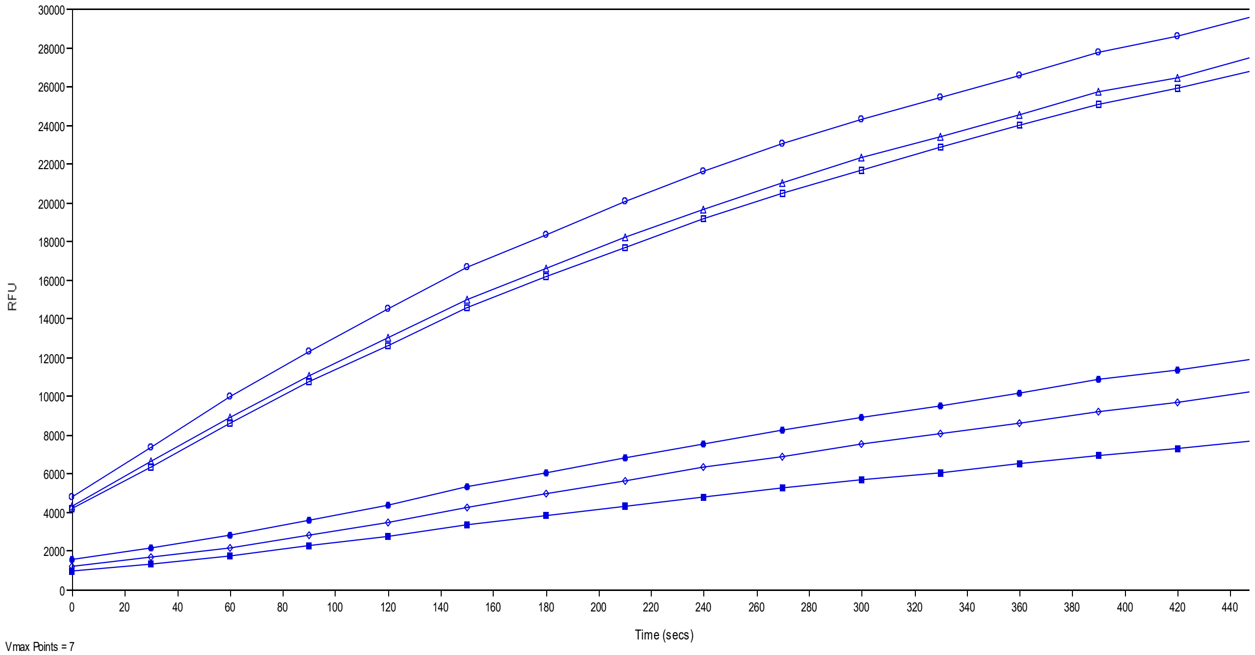

Vmax Points = 7

Well	G1	G2	G3	G10	G11	G12
Vmax Per Second	75.946	67.123	68.710	23.076	26.520	16.878
R^2	0.996	0.997	0.997	1.000	0.999	0.999

Uninhibited β-Gal activity (top curves) and inhibited β-Gal with 50 μM nPEP-1-23 (bottom curves).

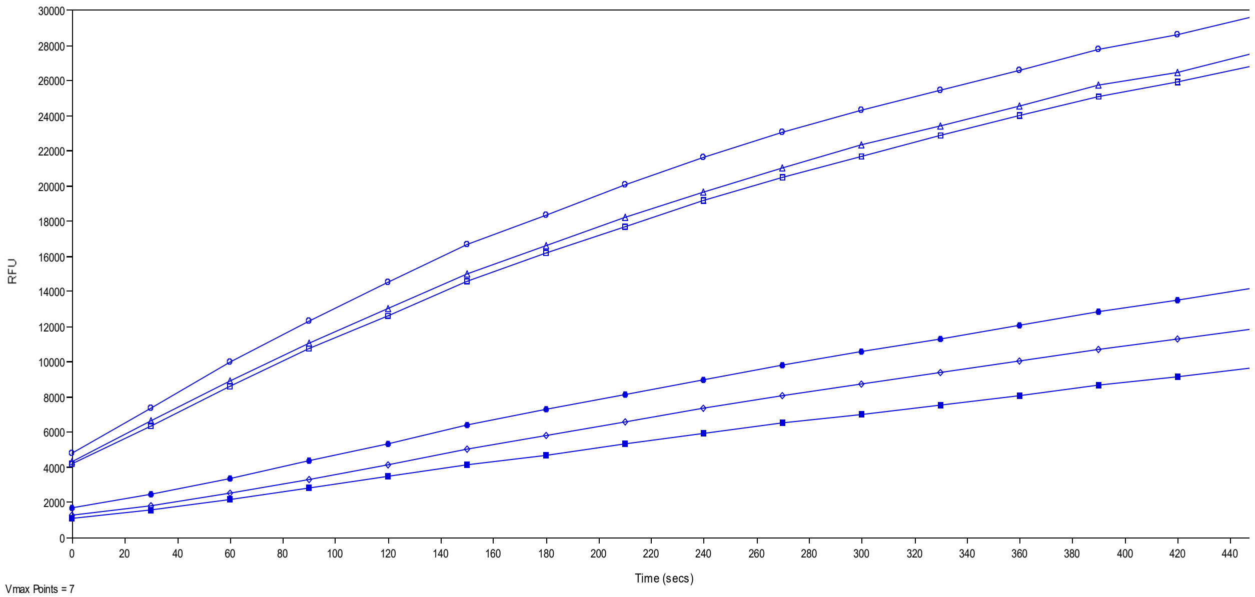

Vmax Points = 7

Well	G1	G2	G3	H10	H11	H12
Vmax Per Second	75.946	67.123	68.710	27.209	31.959	20.952
R^2	0.996	0.997	0.997	0.999	0.999	1.000

Uninhibited β-Gal activity (top curves) and inhibited β-Gal with 50 μM nPEP-1-24 (bottom curves).

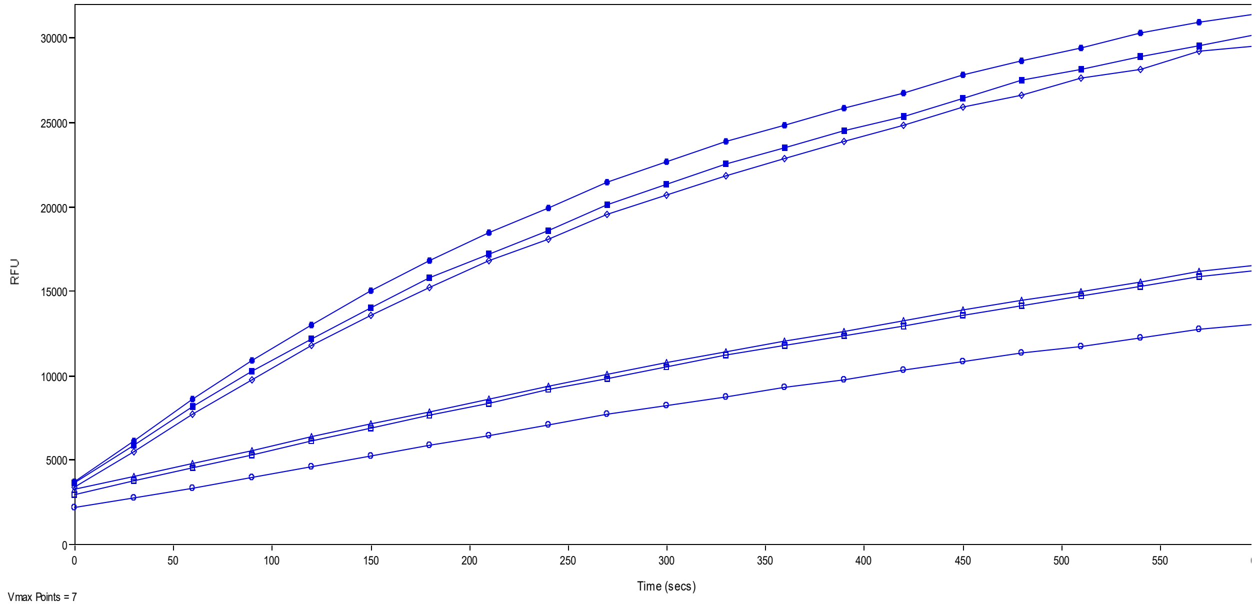

Vmax Points = 7

Well	A4	A5	A6	H1	H2	H3
Vmax Per Second	20.801	26.080	25.636	66.377	73.303	67.402
R^2	1.000	1.000	1.000	0.998	0.997	0.997

Uninhibited β-Gal activity (top curves) and inhibited β-Gal with 50 µM nPEP-1-25 (bottom curves).

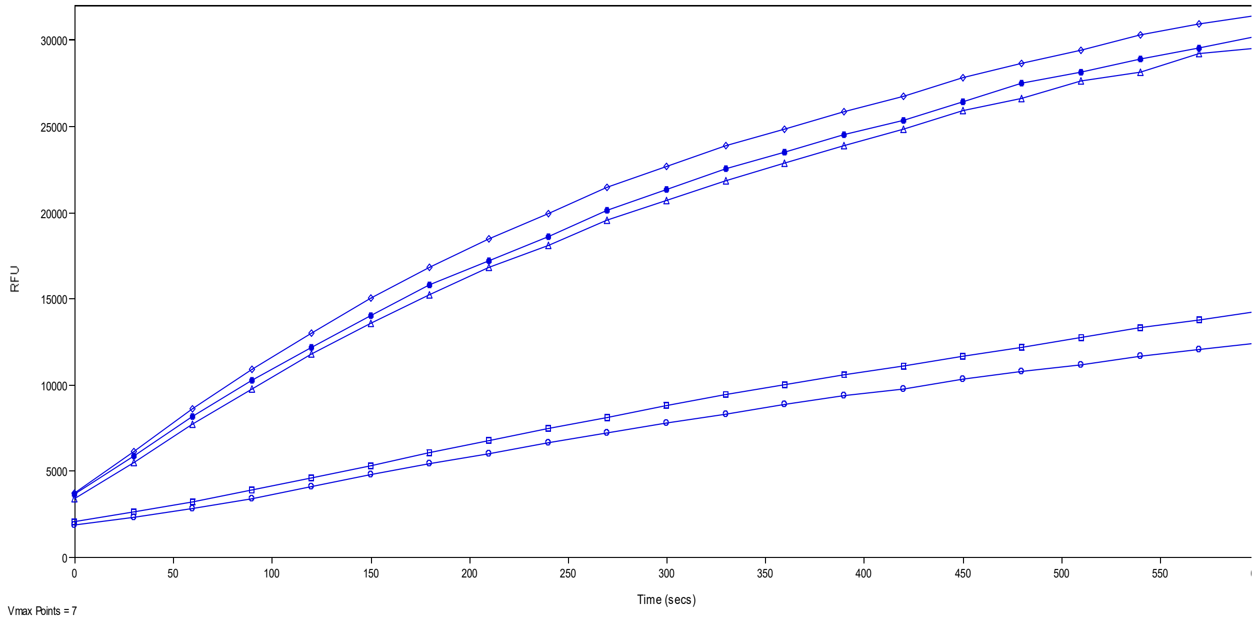

Vmax Points = 7

Well	B5	B6	H1	H2	H3
Vmax Per Second	21.329	23.689	66.377	73.303	67.402
R^2	0.999	1.000	0.998	0.997	0.997

Uninhibited β-Gal activity (top curves) and inhibited β-Gal with 50 µM nPEP-1-26 (bottom curves).

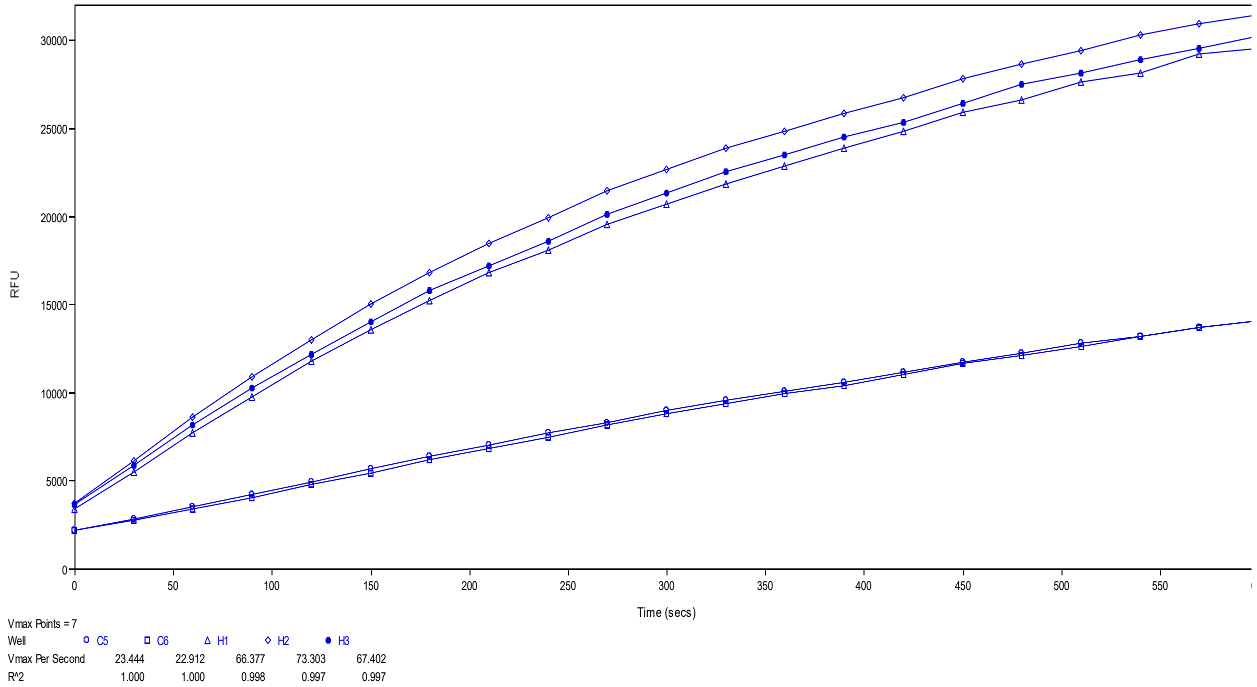

Uninhibited β-Gal activity (top curves) and inhibited β-Gal with 50 μM nPEP-1-27 (bottom curves).

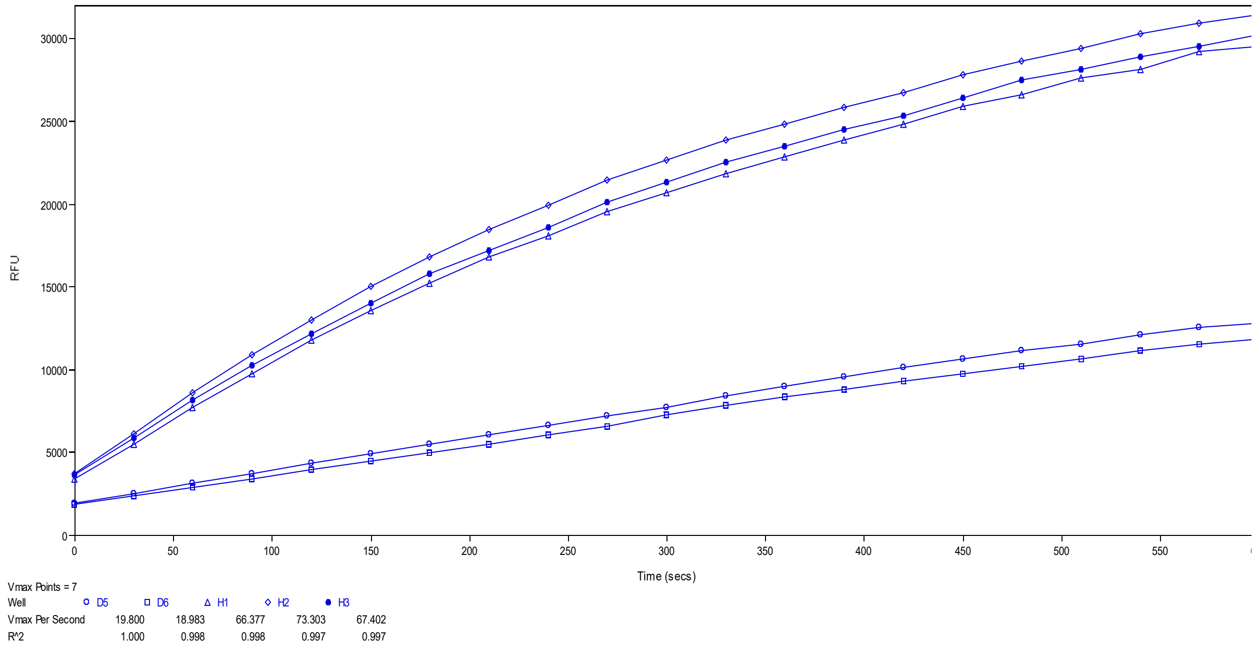

Uninhibited β-Gal activity (top curves) and inhibited β-Gal with 50 μM nPEP-1-28 (bottom curves).

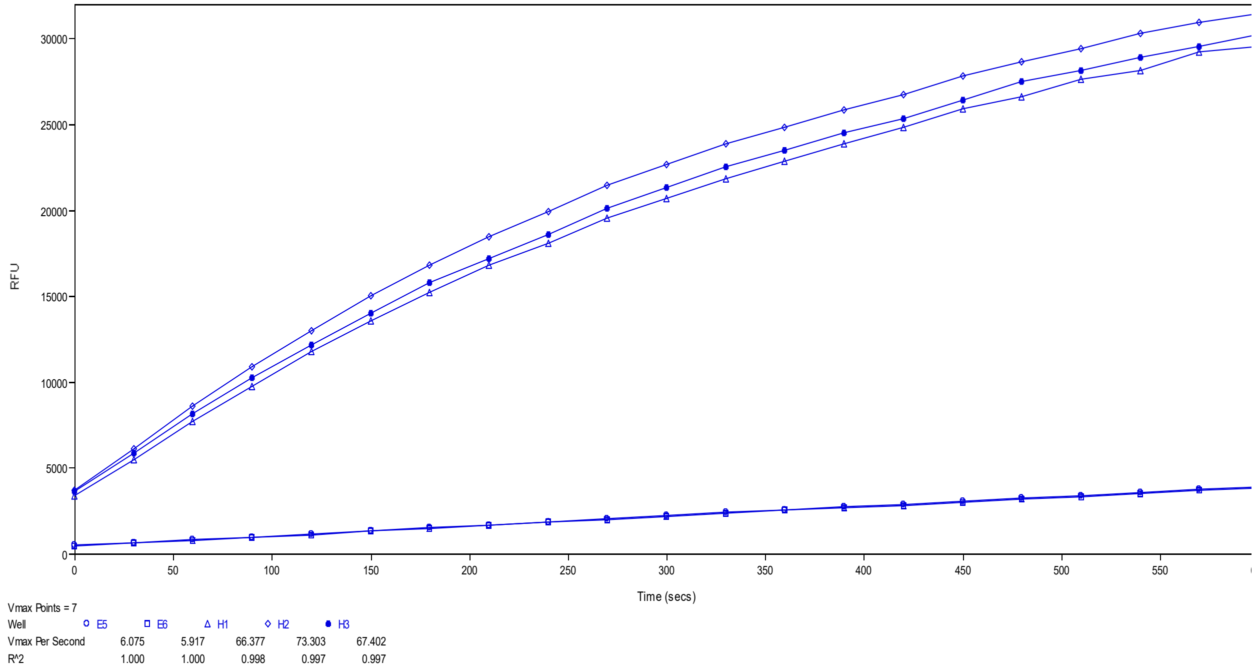

Uninhibited β-Gal activity (top curves) and inhibited β-Gal with 50 μM nPEP-1-29 (bottom curves).

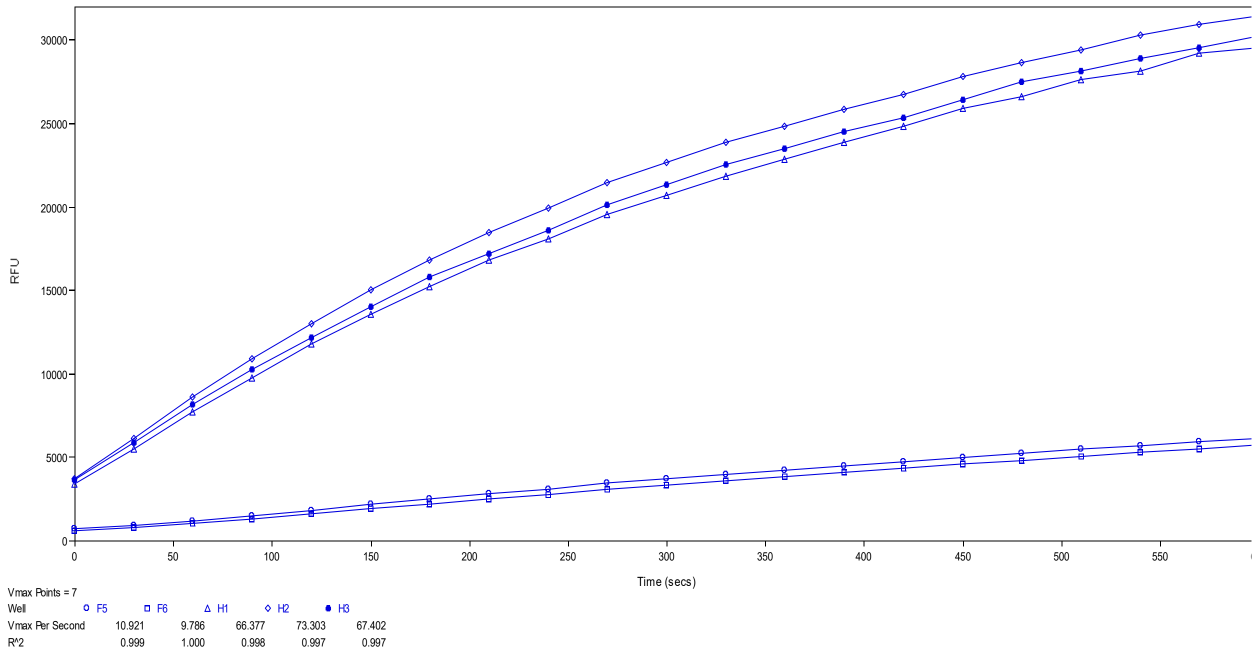

Uninhibited β-Gal activity (top curves) and inhibited β-Gal with 50 μM nPEP-1-30 (bottom curves).

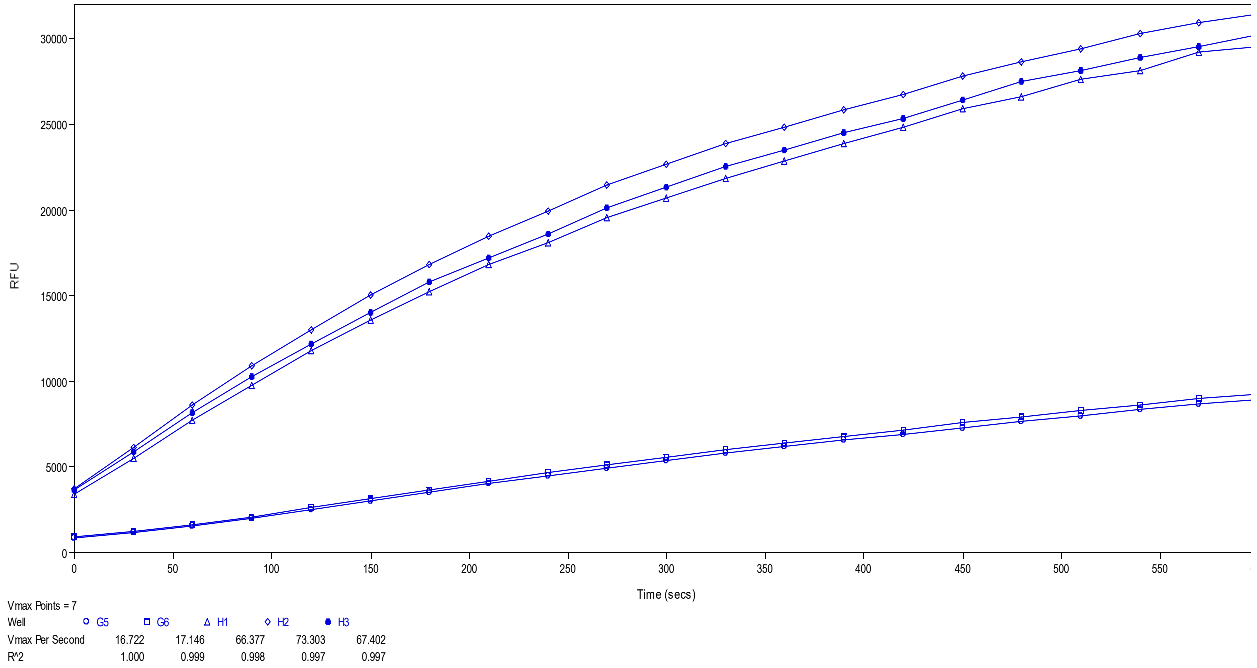

Uninhibited β-Gal activity (top curves) and inhibited β-Gal with 50 μM nPEP-1-31 (bottom curves).

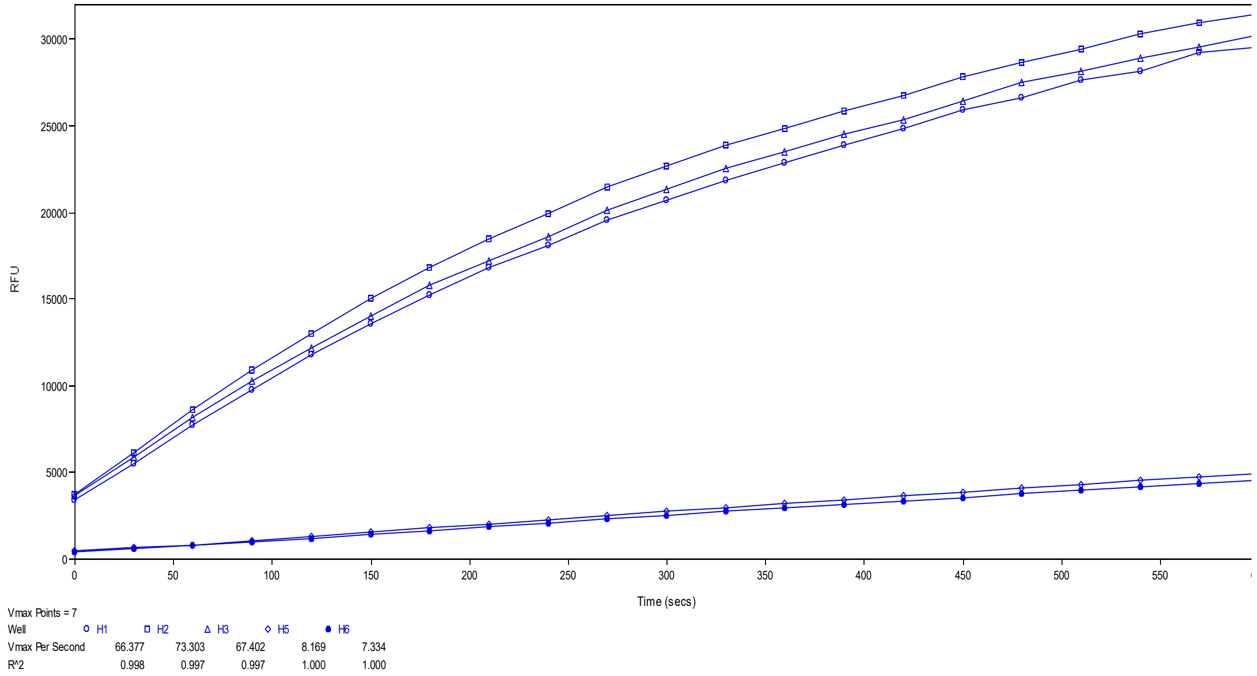

Uninhibited β-Gal activity (top curves) and inhibited β-Gal with 50 μM nPEP-1-32 (bottom curves).

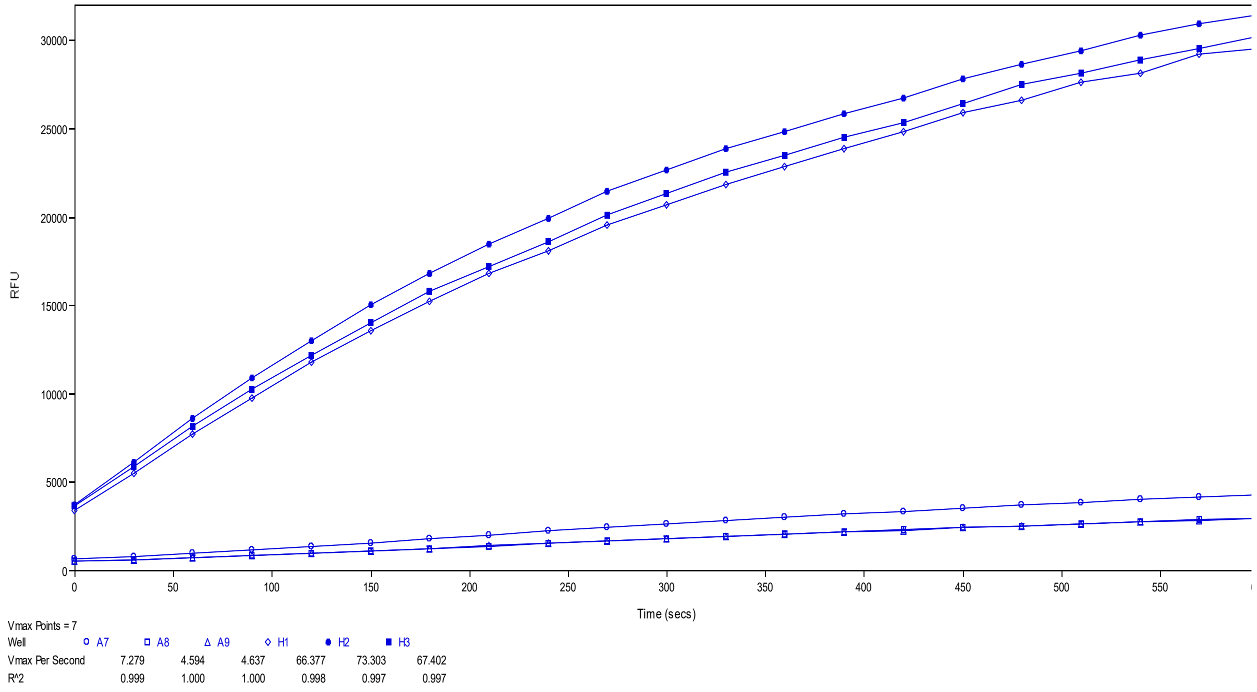

Uninhibited β-Gal activity (top curves) and inhibited β-Gal with 50 μM nPEP-1-33 (bottom curves).

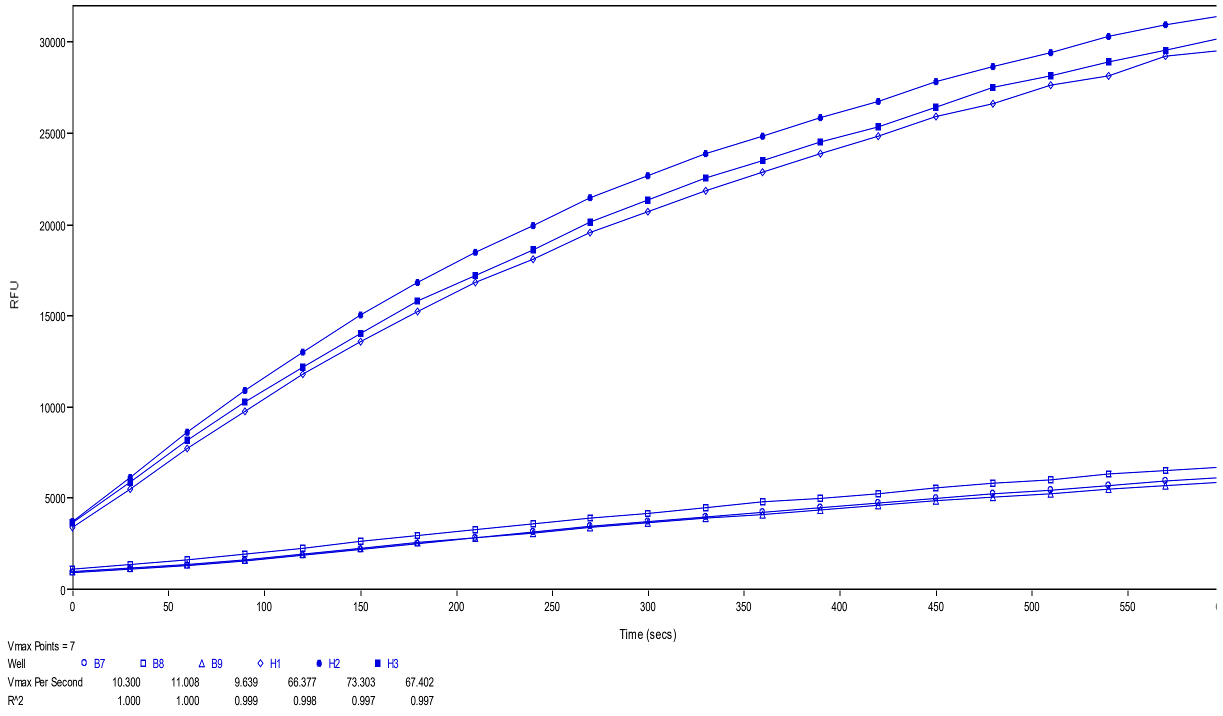

Uninhibited β-Gal activity (top curves) and inhibited β-Gal with 50 μM nPEP-1-34 (bottom).

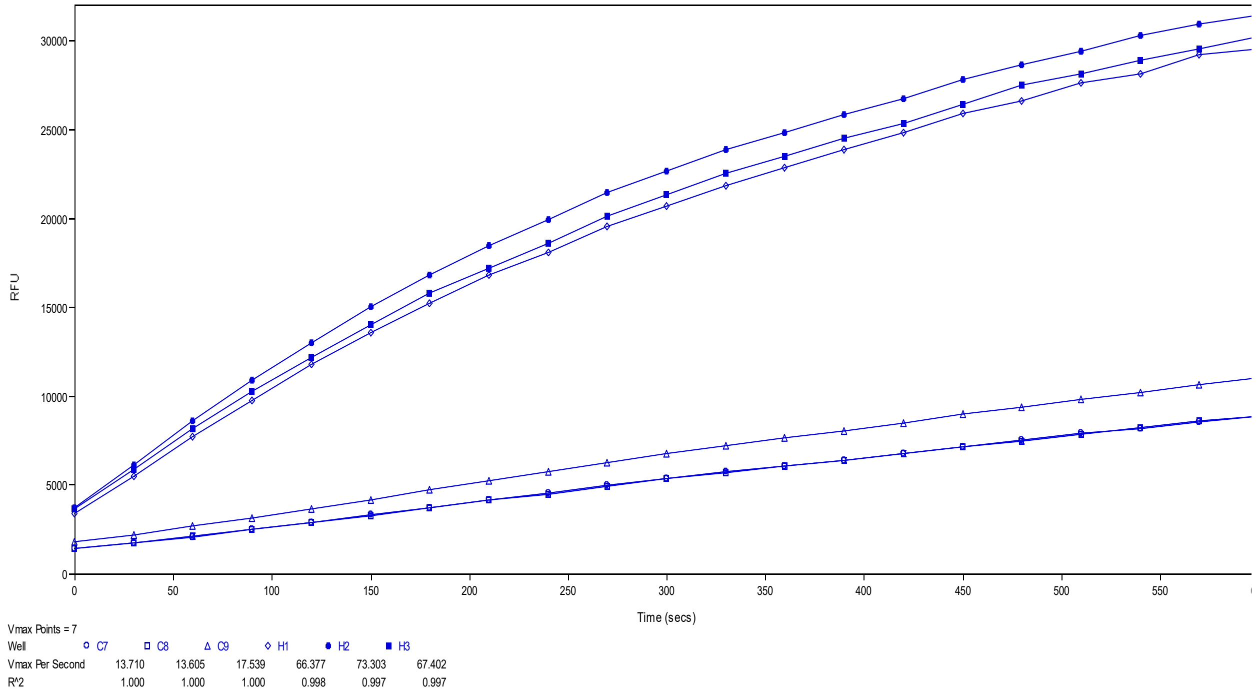

Uninhibited β-Gal activity (top curves) and inhibited β-Gal with 50 μM nPEP-1-35 (bottom curves).

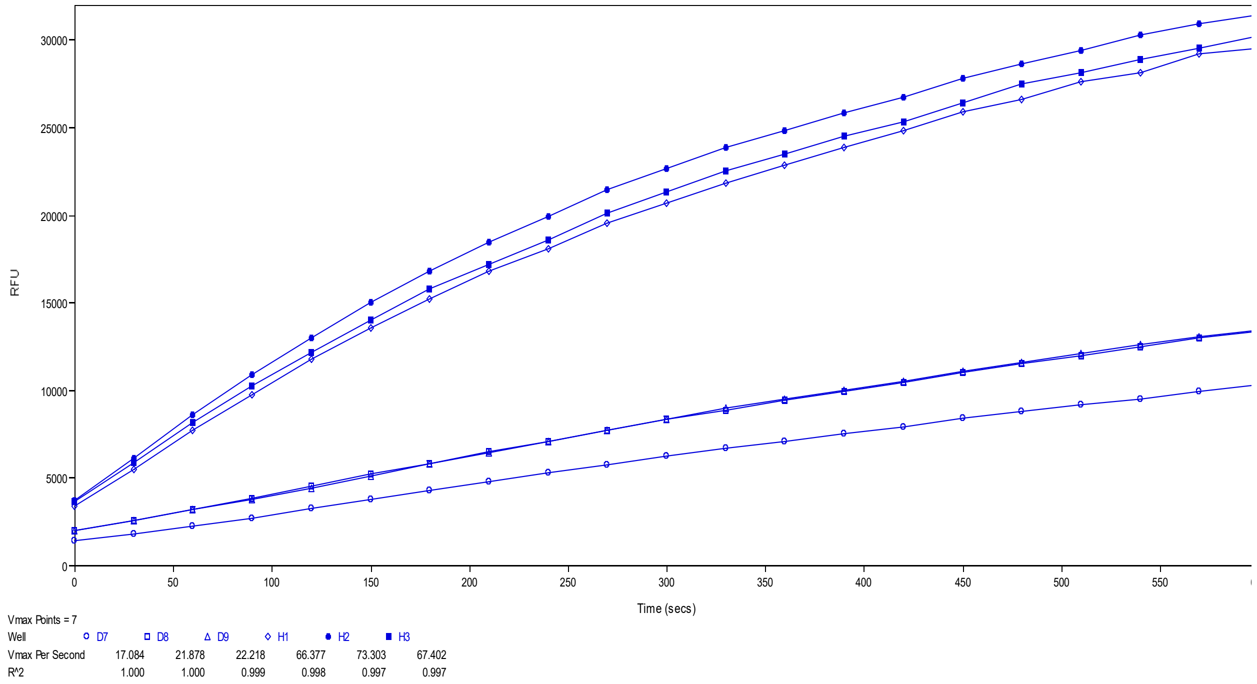

Uninhibited β-Gal activity (top curves) and inhibited β-Gal with 50 μM nPEP-1-36 (bottom).

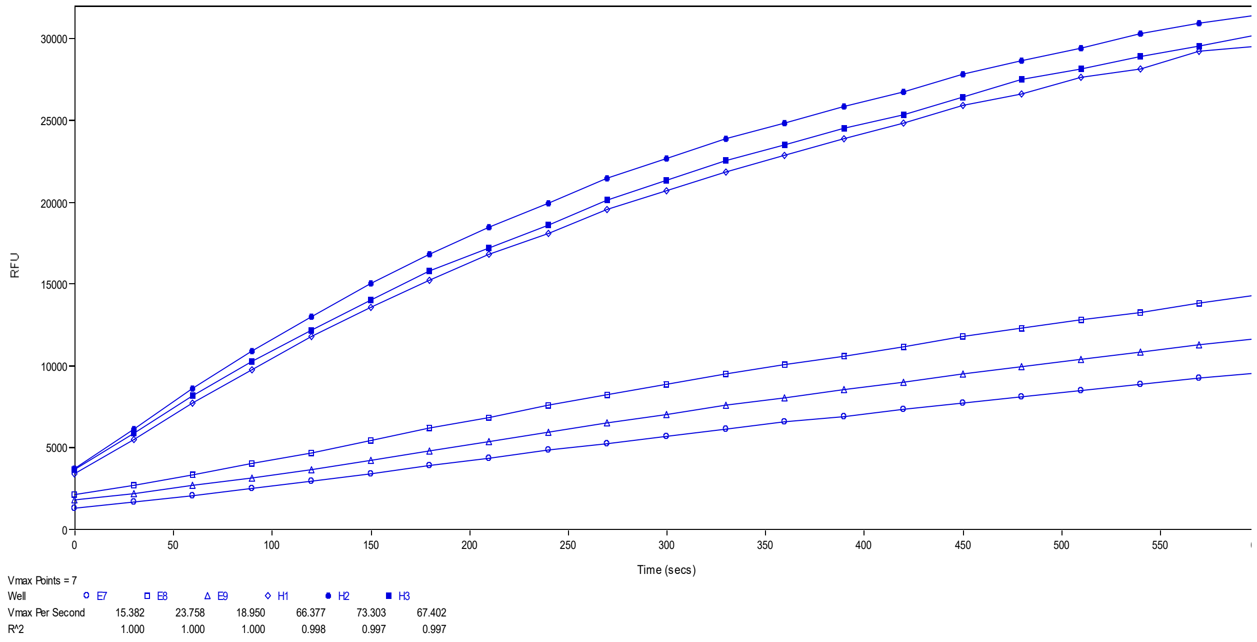

Uninhibited β-Gal activity (top curves) and inhibited β-Gal with 50 μM nPEP-1-37 (bottom curves).

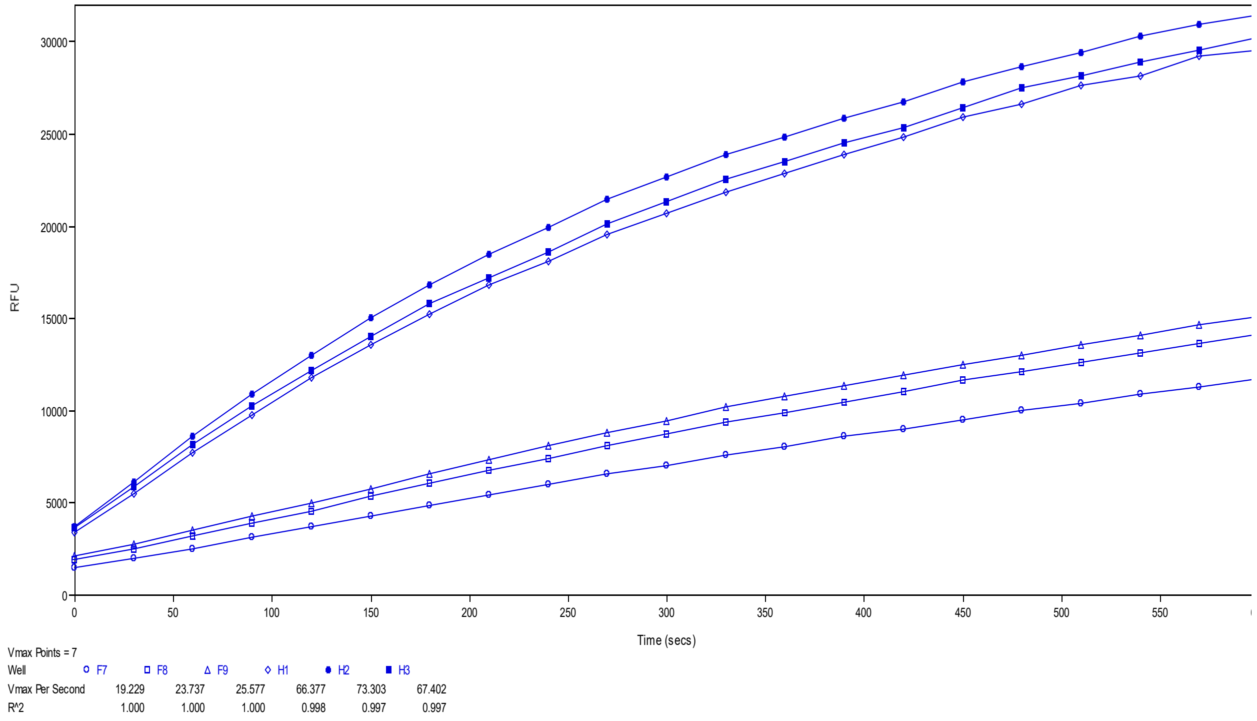

Uninhibited β-Gal activity (top curves) and inhibited β-Gal with 50 μM nPEP-1-38 (bottom curves).

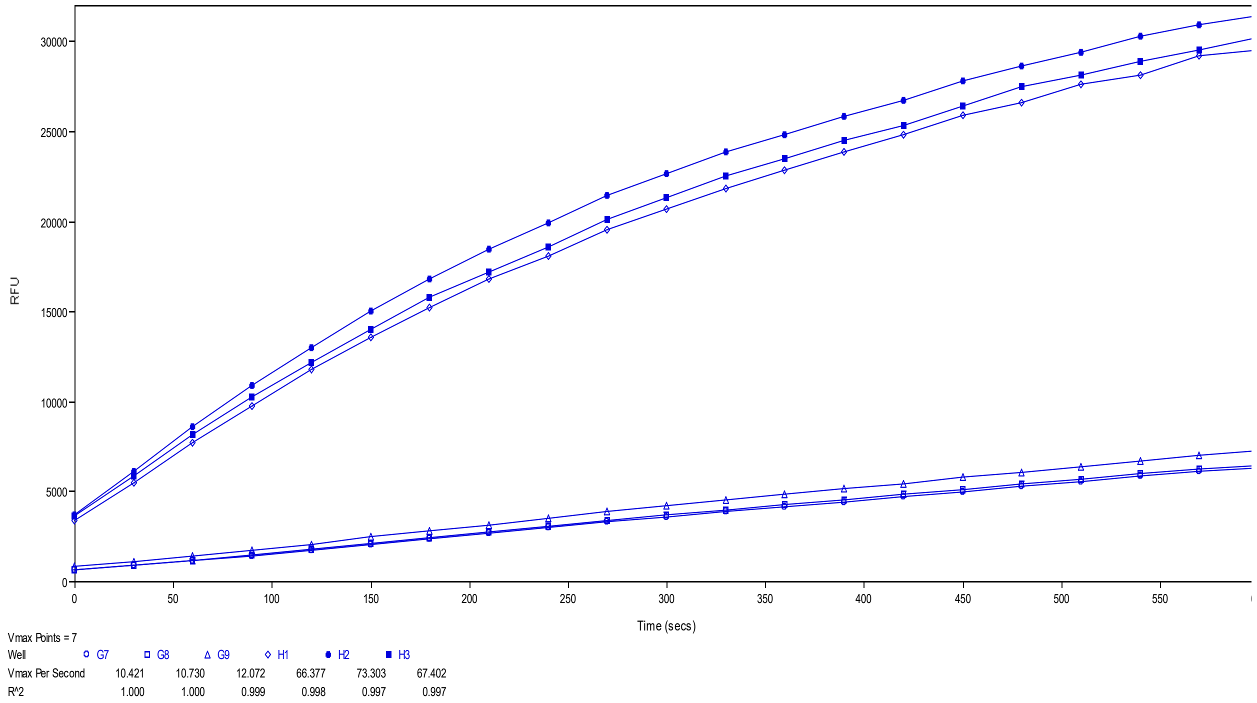

Uninhibited β-Gal activity (top curves) and inhibited β-Gal with 50 μM nPEP-1-39 (bottom curves).

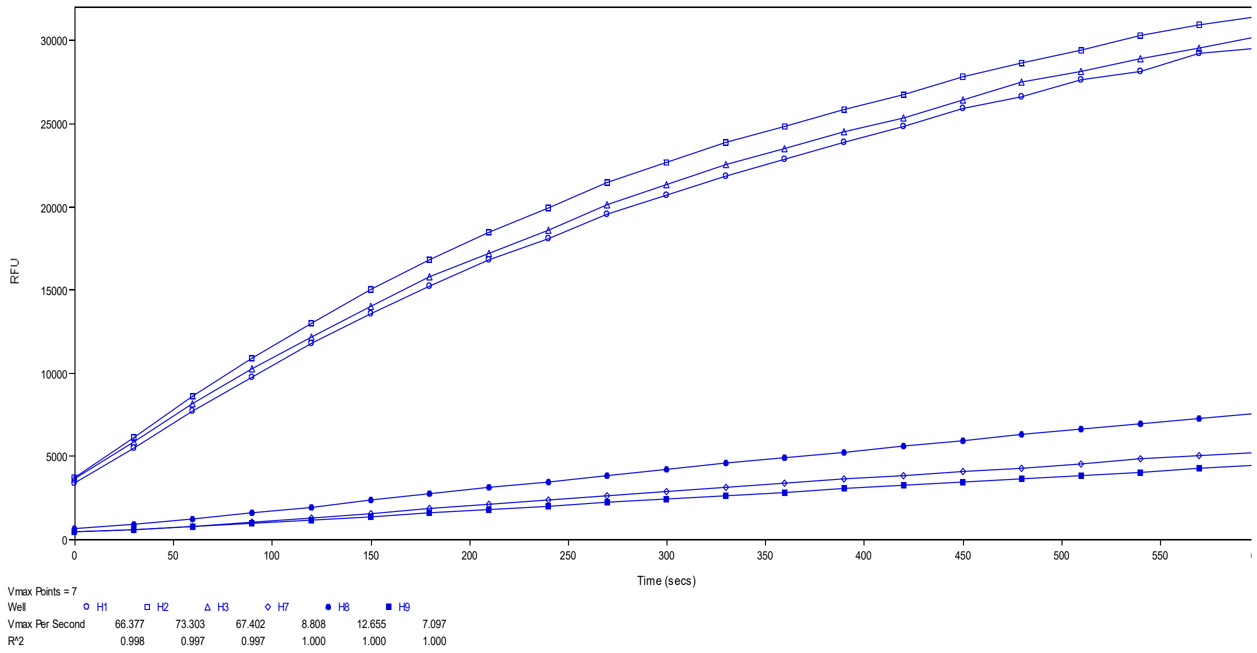

Uninhibited β-Gal activity (top curves) and inhibited β-Gal with 50 μM nPEP-1-40 (bottom curves).

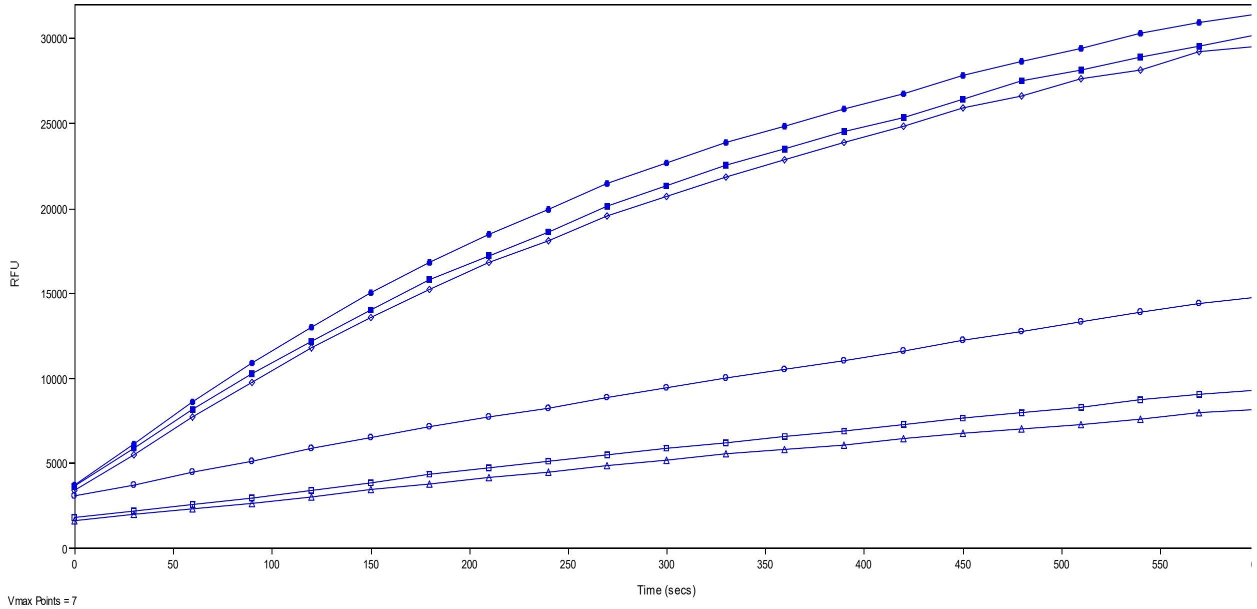

Vmax Points = 7

Well	○ A10	□ A11	△ A12	◇ H1	● H2	■ H3
Vmax Per Second	22.882	14.545	12.383	66.377	73.303	67.402
R^2	0.999	0.999	0.999	0.998	0.997	0.997

Uninhibited β-Gal activity (top curves) and inhibited β-Gal with 50 µM nPEP-1-41 (bottom curves).

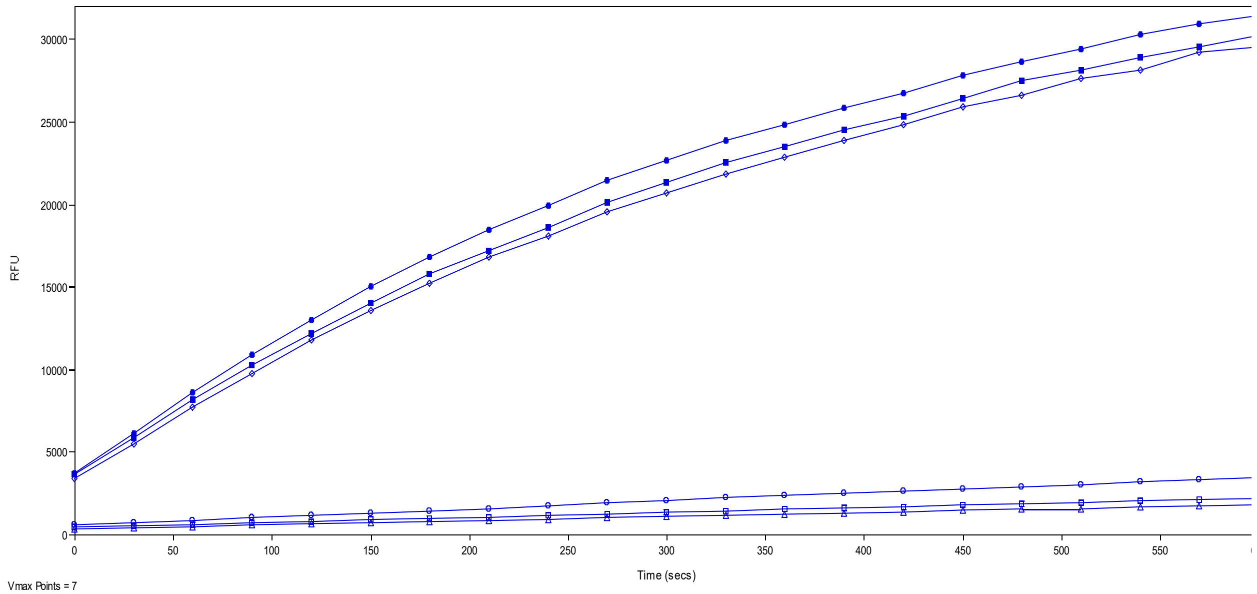

Vmax Points = 7

Well	○ B10	□ B11	△ B12	◇ H1	● H2	■ H3
Vmax Per Second	5.262	3.057	2.536	66.377	73.303	67.402
R^2	0.999	1.000	1.000	0.998	0.997	0.997

Uninhibited β-Gal activity (top curves) and inhibited β-Gal with 50 µM nPEP-1-42 (bottom curves).

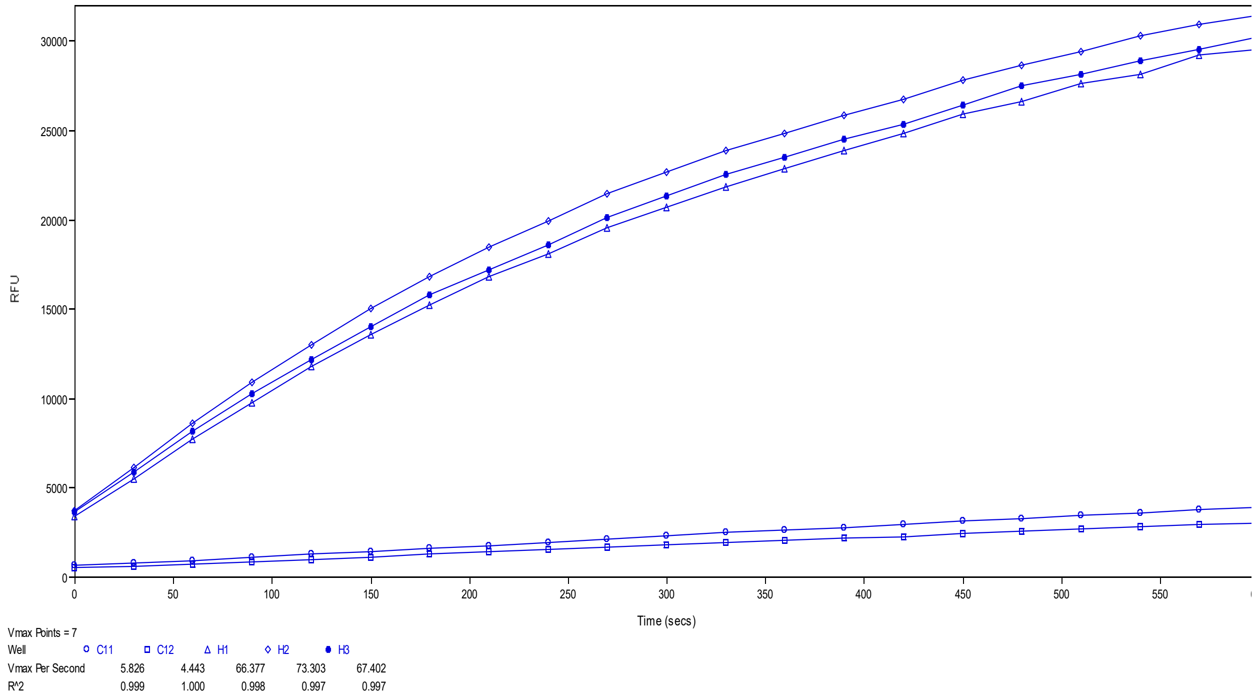

Uninhibited β-Gal activity (top curves) and inhibited β-Gal with 50 μM nPEP-1-43 (bottom curves).

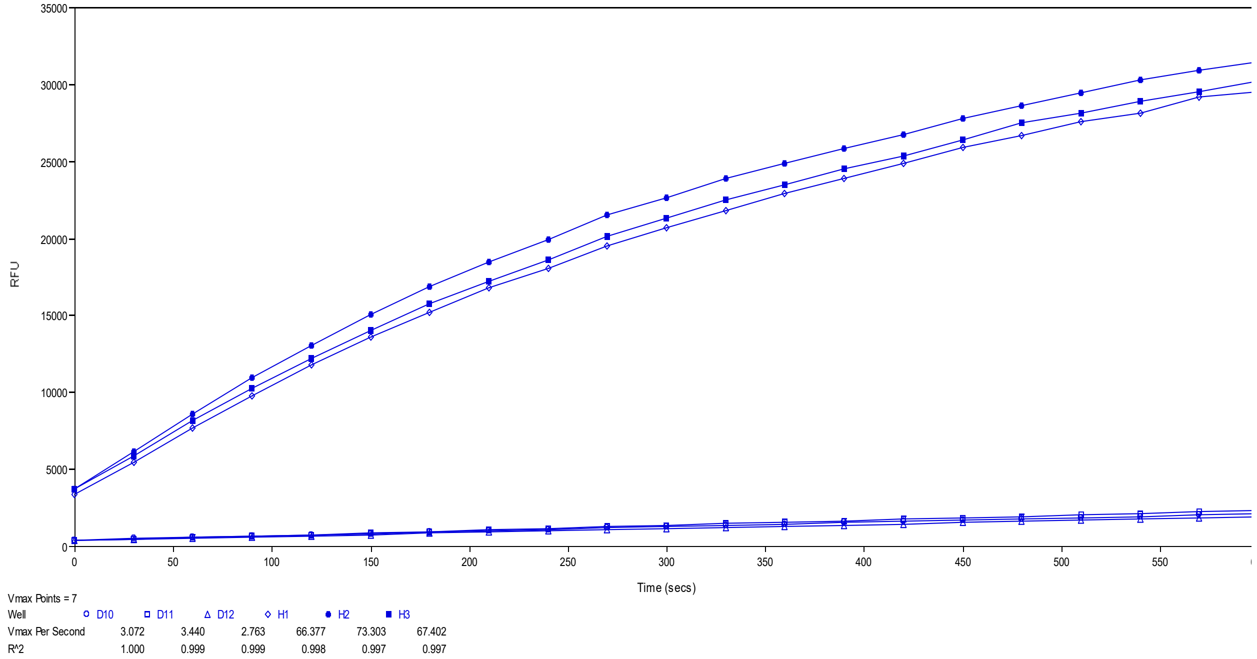

Uninhibited β-Gal activity (top curves) and inhibited β-Gal with 50 μM nPEP-1-44 (bottom curves).

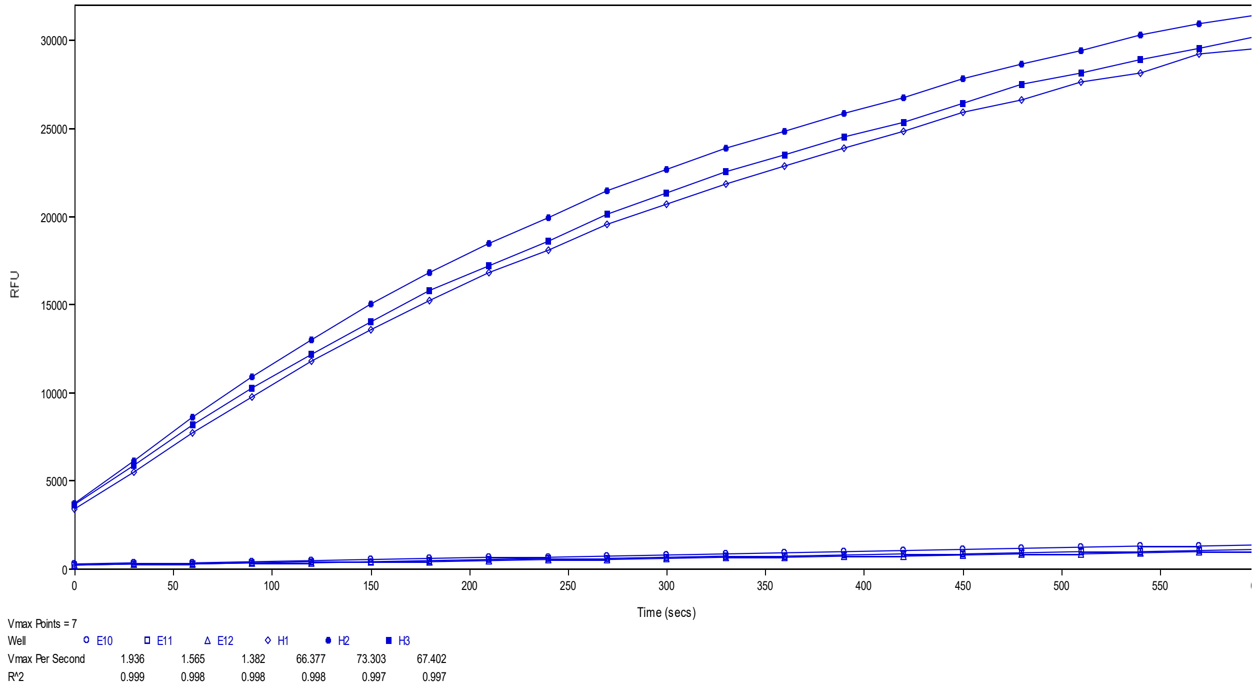

Uninhibited β-Gal activity (top curves) and inhibited β-Gal with 50 μM nPEP-1-45 (bottom curves).

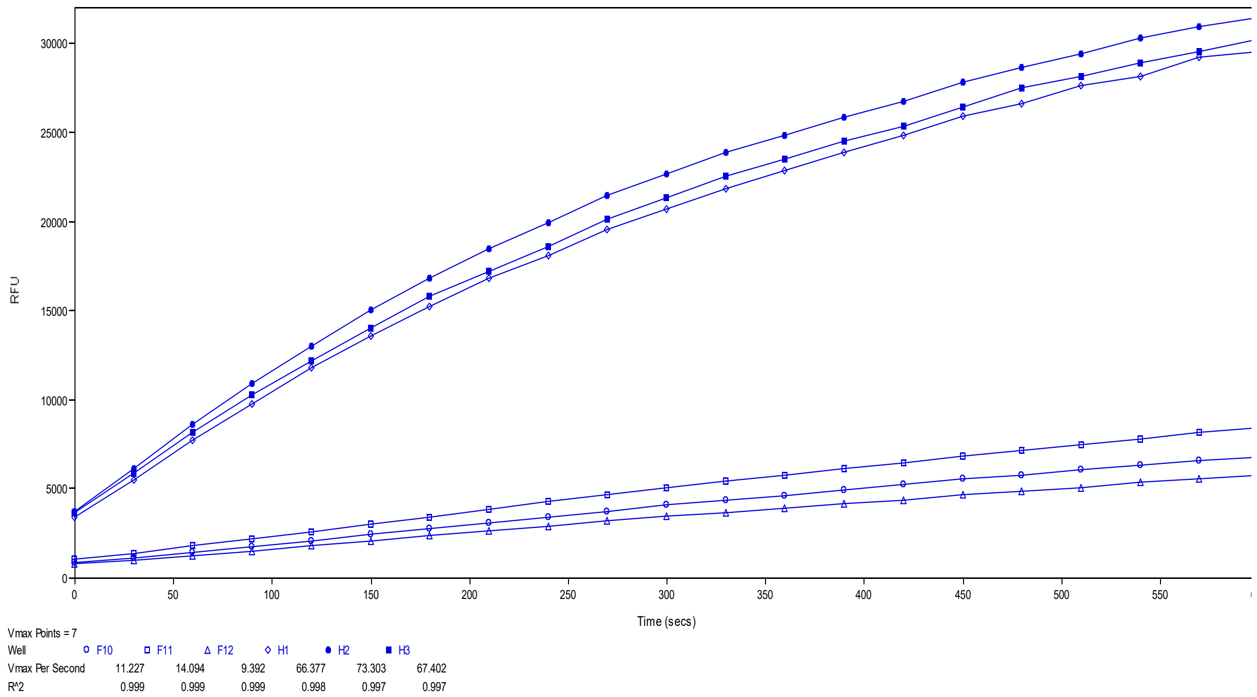

Uninhibited β-Gal activity (top curves) and inhibited β-Gal with 50 μM nPEP-1-46 (bottom).
