# Supplementary material for: Computational and experimental analysis of short peptide motifs for enzyme inhibition
Source: PLoS One. 2017 Aug 15;12(8):e0182847. doi: 10.1371/journal.pone.0182847 (PMC5557489; doi:10.1371/journal.pone.0182847)
Supplement: S1 Table — (PDF) [file pone.0182847.s009.pdf]

S1 Table. Truncation library of PEP-1.

PEP-1	RVFKRYKRWLHVSRYYFGSC
tPEP-1-1	FKRYKRWLHVSRYYFGSC
tPEP-1-2	RYKRWLHVSRYYFGSC
tPEP-1-3	KRWLHVSRYYFGSC
tPEP-1-4	WLHVSRYYFGSC
tPEP-1-5	HVSRYYFGSC
tPEP-1-6	SRYYFGSC
tPEP-1-7	RVFKRYKRWLHVSRYGSC
tPEP-1-8	RVFKRYKRWLHVSGSC
tPEP-1-9	RVFKRYKRWLHGSC
tPEP-1-10	RVFKRYKRWGSC
tPEP-1-11	RVFKRYKGSC
tPEP-1-12	RVFKRGSC
