# Supplementary material for: Computational and experimental analysis of short peptide motifs for enzyme inhibition
Source: PLoS One. 2017 Aug 15;12(8):e0182847. doi: 10.1371/journal.pone.0182847 (PMC5557489; doi:10.1371/journal.pone.0182847)
Supplement: S2 Table — (PDF) [file pone.0182847.s010.pdf]

S2 Table. Truncation library of PEP-2.

PEP-2	PASMFSYFKKQGYYYKLGSC
tPEP-2-1	SMFSYFKKQGYYYKLGSC
tPEP-2-2	FSYFKKQGYYYKLGSC
tPEP-2-3	YFKKQGYYYKLGSC
tPEP-2-4	KKQGYYYKLGSC
tPEP-2-5	QGYYYKLGSC
tPEP-2-6	YYYKLGSC
tPEP-2-7	PASMFSYFKKQGYYYGSC
tPEP-2-8	PASMFSYFKKQGYGSC
tPEP-2-9	PASMFSYFKKQGSC
tPEP-2-10	PASMFSYFKGSC
tPEP-2-11	PASMFSYGSC
tPEP-2-12	PASMFGSC
