# Supplementary material for: Computational and experimental analysis of short peptide motifs for enzyme inhibition
Source: PLoS One. 2017 Aug 15;12(8):e0182847. doi: 10.1371/journal.pone.0182847 (PMC5557489; doi:10.1371/journal.pone.0182847)
Supplement: S3 Table — (PDF) [file pone.0182847.s011.pdf]

S3 Table. Alanine Scan library of PEP-1.

PEP-1	RVFKRYKRWLHVSRYYFGSC
aPEP-1-1	AVFKRYKRWLHVSRYYFGSC
aPEP-1-2	RAFKRYKRWLHVSRYYFGSC
aPEP-1-3	RVAKRYKRWLHVSRYYFGSC
aPEP-1-4	RVFARYKRWLHVSRYYFGSC
aPEP-1-5	RVFKAYKRWLHVSRYYFGSC
aPEP-1-6	RVFKRAKRWLHVSRYYFGSC
aPEP-1-7	RVFKRYARWLHVSRYYFGSC
aPEP-1-8	RVFKRYKAWLHVSRYYFGSC
aPEP-1-9	RVFKRYKRALHVSRYYFGSC
aPEP-1-10	RVFKRYKRWAHVSRYYFGSC
aPEP-1-11	RVFKRYKRWLAVSRYYFGSC
aPEP-1-12	RVFKRYKRWLHASRYYFGSC
aPEP-1-13	RVFKRYKRWLHVARYYFGSC
aPEP-1-14	RVFKRYKRWLHVSAYYFGSC
aPEP-1-15	RVFKRYKRWLHVSRAYFGSC
aPEP-1-16	RVFKRYKRWLHVSRYAFGSC
aPEP-1-17	RVFKRYKRWLHVSRYYAGSC
