# Supplementary material for: Computational and experimental analysis of short peptide motifs for enzyme inhibition
Source: PLoS One. 2017 Aug 15;12(8):e0182847. doi: 10.1371/journal.pone.0182847 (PMC5557489; doi:10.1371/journal.pone.0182847)
Supplement: S4 Table — (PDF) [file pone.0182847.s012.pdf]

S4 Table. Alanine Scan library of PEP-2.

PEP-2	PASMFSYFKKQGYYYKLGSC
aPEP-2-1	AASMFSYFKKQGYYYKLGSC
aPEP-2-2	PAAMFSYFKKQGYYYKLGSC
aPEP-2-3	PASAFSYFKKQGYYYKLGSC
aPEP-2-4	PASMASYFKKQGYYYKLGSC
aPEP-2-5	PASMFAYFKKQGYYYKLGSC
aPEP-2-6	PASMFSAFKKQGYYYKLGSC
aPEP-2-7	PASMFSYAKKQGYYYKLGSC
aPEP-2-8	PASMFSYFAKQGYYYKLGSC
aPEP-2-9	PASMFSYFKAQGYYYKLGSC
aPEP-2-10	PASMFSYFKKAGYYYKLGSC
aPEP-2-11	PASMFSYFKKQAYYYKLGSC
aPEP-2-12	PASMFSYFKKQGAYYKLGSC
aPEP-2-13	PASMFSYFKKQGYAYKLGSC
aPEP-2-14	PASMFSYFKKQGYYAKLGSC
aPEP-2-15	PASMFSYFKKQGYYYALGSC
aPEP-2-16	PASMFSYFKKQGYYYKAGSC
