# Supplementary material for: Computational and experimental analysis of short peptide motifs for enzyme inhibition
Source: PLoS One. 2017 Aug 15;12(8):e0182847. doi: 10.1371/journal.pone.0182847 (PMC5557489; doi:10.1371/journal.pone.0182847)
Supplement: S5 Table — (PDF) [file pone.0182847.s013.pdf]

S5 Table. Point-variant library of nPEP-1 “FKRYKRWGSG” at each of the 7 N-terminal positions with a substitution set of S, Y, E, L, W, Q, and R.

nPEP-1-1	**FKRYKRWGSC**	nPEP-1-24	FKLYKRWGSC
nPEP-1-2	SKRYKRWGSC	nPEP-1-25	FKRLKRWGSC
nPEP-1-3	FSRYKRWGSC	nPEP-1-26	FKRYLRWGSC
nPEP-1-4	FKSYKRWGSC	nPEP-1-27	FKRYKLWGSC
nPEP-1-5	FKRSKRWGSC	nPEP-1-28	FKRYKRLGSC
nPEP-1-6	FKRYSRWGSC	nPEP-1-29	WKRYKRWGSC
nPEP-1-7	FKRYKSWGSC	nPEP-1-30	FWRYKRWGSC
nPEP-1-8	FKRYKRSGSC	nPEP-1-31	FKWYKRWGSC
nPEP-1-9	YKRYKRWGSC	nPEP-1-32	FKRWKRWGSC
nPEP-1-10	FYRYKRWGSC	nPEP-1-33	FKRYWRWGSC
nPEP-1-11	FKYYKRWGSC	nPEP-1-34	FKRYKWWGSC
nPEP-1-12	FKRYYRWGSC	nPEP-1-35	QKRYKRWGSC
nPEP-1-13	FKRYKYWGSC	nPEP-1-36	FQRYKRWGSC
nPEP-1-14	FKRYKRYGSC	nPEP-1-37	FKQYKRWGSC
nPEP-1-15	EKRYKRWGSC	nPEP-1-38	FKRQKRWGSC
nPEP-1-16	FERYKRWGSC	nPEP-1-39	FKRYQRWGSC
nPEP-1-17	FKEYKRWGSC	nPEP-1-40	FKRYKQWGSC
nPEP-1-18	FKREKRWGSC	nPEP-1-41	FKRYKRQGSC
nPEP-1-19	FKRYERWGSC	nPEP-1-42	RKRYKRWGSC
nPEP-1-20	FKRYKEWGSC	nPEP-1-43	FRRYKRWGSC
nPEP-1-21	FKRYKREGSC	nPEP-1-44	FKRRKRWGSC
nPEP-1-22	LKRYKRWGSC	nPEP-1-45	FKRYRRWGSC
nPEP-1-23	FLRYKRWGSC	nPEP-1-46	FKRYKRRGSC
